# Simple Zn-Mediated Seleno- and Thio-Functionalization of Steroids at C-1 Position

**DOI:** 10.3390/ijms23063022

**Published:** 2022-03-11

**Authors:** Paweł A. Grześ, Bonifacio Monti, Natalia Wawrusiewicz-Kurylonek, Luana Bagnoli, Luca Sancineto, Izabella Jastrzebska, Claudio Santi

**Affiliations:** 1 Faculty of Chemistry, University of Białystok, ul. Ciołkowskiego 1K, 15-245 Białystok, Poland; pawel.grzes1993@gmail.com; 2 Group of Catalysis, Synthesis and Organic Green Chemistry, Department of Pharmaceutical Sciences, University of Perugia, Via del Liceo 1, 06132 Perugia, Italy; bonifaciomonti@gmail.com (B.M.); luana.bagnoli@unipg.it (L.B.); luca.sancineto@unipg.it (L.S.); 3 Department of Clinical Genetics, Diabetology and Internal Medicine, Medical University of Białystok, Skłodowska–Curie 24A, 15-276 Białystok, Poland; natalia.wawrusiewicz-kurylonek@umb.edu.pl; 4 Department of Endocrinology, Diabetology and Internal Medicine, Medical University of Białystok, Skłodowska–Curie 24A, 15-276 Białystok, Poland

**Keywords:** selenium, sulfur, zinc, steroids, Michael additions

## Abstract

Here we report the reaction in the biphasic system of the in situ prepared selenols and thiols with 1,4-androstadiene-3,17-dione (**1**) or prednisone acetate (**2**) having α,β-unsaturated ketone as an electrophilic functionalization. The Michael-type addition reaction resulted to be chemo- and stereoselective, affording a series of novel steroidal selenides and sulfides. This is an example of a one-step, eco-friendly process that bypasses some of the main concerns connected with the bad smell and the toxicity of these seleno- and thio-reagents. Furthermore, we demonstrated that the proposed methodology offers the possibility to prepare libraries of steroids variously and selectively decorated with different organochalcogen moieties at the C1 position starting from 1,4-androstadienic skeletons and leaving unaltered the C4–C5 unsaturation. Based on the data reported in the literature the introduction of an organoselenium or an organosulfur moiety in a steroid could provide new interesting pharmaceutically active entities exerting anticancer and antimicrobial activities. In this optic, new synthetic strategies to efficiently prepare this class of compounds could be strongly desirable.

## 1. Introduction

In the last decades different classes of organoselenium compounds were investigated for biological purposes, evidencing, besides the antioxidant properties [1], some promising activities, such as antiviral, antibacterial and anticancer [2,3].

**Figure 1:**
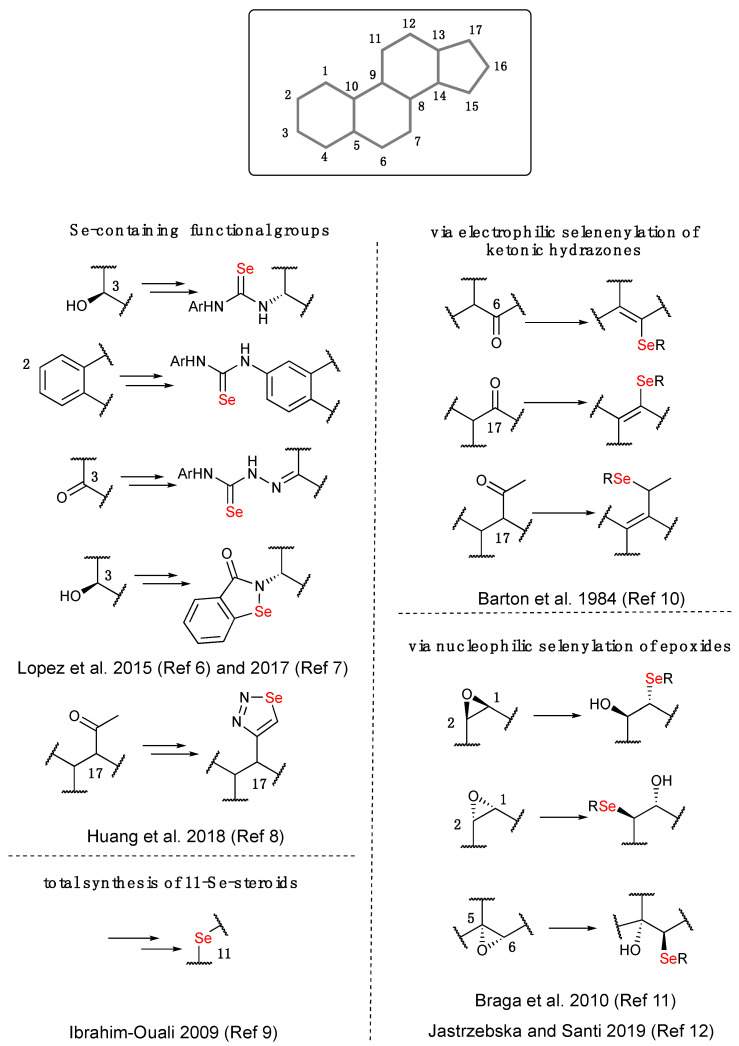
Examples of Se-functionalization on cyclopentanoperhydro-phenanthrene skeleton.

Similarly, the cyclopenta[a]phenanthrene skeleton is a privileged core structure present in several pharmacologically relevant molecules as well as in some commercially available drugs and/or hormones such as glucocorticoids, steroidal anti-inflammatories or cardiac steroids [4]. On the basis of these considerations, a hybrid formed by placing a Se- or a S-moiety to a steroidal structure may have enhanced biological properties when compared to the native fragments [5]. Consequently, improving synthetic tools in order to enable the chemo regio- and stereoselective preparation of novel selenium- and sulfur-containing libraries of steroids is particularly challenging for the exploration of the chemical space in the discovery of novel biologically active compounds. Right now, a small number of selenosteroids are reported in the literature, and some general examples of functionalization on different carbons of the cyclopentanoperhydro-phenanthrene skeleton are summarized in Figure 1. Selenium can be contained in functionalized selenoureas [6], or heterocycles such as *N*-linked selenoxazoles [7] or 1,2,3-selenodiazoles [8] that are generally introduced using multistep procedures. Ibrahim-Ouali in 2009 described the first total synthesis of 11-selenosteroids as the unique example in which a carbon of the steroidal skeleton is substituted with a selenium atom [9].

In other examples, electrophilic and or nucleophilic selenium reagents were directly introduced in the structures using the reactivity of the ketonic hydrazones [10] and of the epoxides, respectively [11].

More specifically, Barton reported the conversion of C-6 and C-17 keto groups into vinyl selenide steroidal systems [10], and Braga and coworkers prepared a series of selenocholestane derivatives by the stereoselective ring opening reaction of 5α,6α epoxide with selenolates, generated in situ by the NaBH_4_-mediated reduction of diphenyldiselenide (PhSe)_2_ and other, differently functionalized diselenides [11]. This chemistry was expanded at different positions within the steroidal core by some of us who recently reported the epoxide transformation exploiting the reactivity of PhSeZnCl, obtaining selenosteroids endowed with antibiofilm activity [12,13].

With the aim to develop a novel procedure to prepare hybrid derivatives [5], we report here the functionalization of the steroidal core of the biologically relevant androstadiene and prednisone bearing an α β-unsaturated keto system, which underwent Seleno-Michael or Thio-Michael addition by treatment with selenolates and thiolates generated in situ using a previously reported acidic biphasic system that was extensively used for the selenenylation of different classes of organic compounds [14,15,16,17,18,19,20].

## 2. Results and Discussion

**Figure 2:**
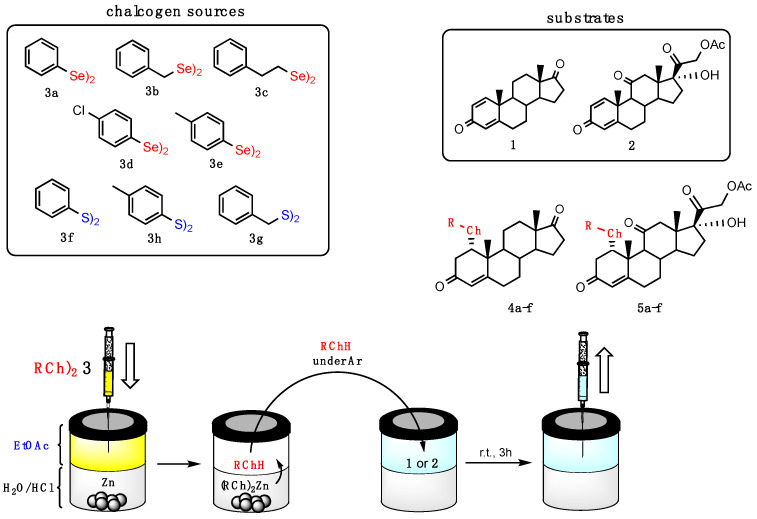
Addition reaction of nucleophilic reagents prepared in situ from the dichalcogenides **3a**–**g** to the Michael acceptors **1** and **2** affording the target compounds **4a**–**f** and **5a**–**g,** respectively (the scopes are reported in Table 1, Table 2 and Table 3).

For the current investigation we slightly modified the procedure recently reported by some of us for the conjugated nucleophilic addition of selenolates [20]. A biphasic system composed by the same volume of ethyl acetate and 10% HCl, containing PhSe_2_ (**3a**) and 10 equiv. of zinc shaves was stirred until complete discoloration of the organic layer. The liquid phase was transferred under argon atmosphere into a flask containing the substrate: 1,4-androstadiene-3,17-dione (**1**) or prednisone acetate (**2**). The resulting reaction mixture was stirred at room temperature for 3 h (Figure 2). When compound **1** was used as starting material, the formation of steroidal selenide **4a** was regio- and stereoselectively obtained and isolated in 70% yield after chromatographic purification, having a physical and spectroscopic date fully coherent with those reported in the literature [21]. The selenenylation afforded only the α diastereisomer at C1 carbon as a consequence of the steric hindrance at the electrophilic carbons.

The scope of the reaction was investigated by the use of commercially available diselenide (**3a**) or diselenides prepared according to the literature (**3b**–**e**) [22]. Diphenyldiselenide (**3a**) and diaryldiselenides bearing both electron withdrawing (**3d**) or donating (**3e**) substituents afforded the corresponding selenenylated steroids **4a**, **4d** and **4e**, in good yields (Table 1 entries 1, 4 and 5). On the contrary, dibenzyldiselenide (**3b**) and bis(2-phenylethyl)diselenide (**3c**) gave the target compounds only in moderate yields (Table 1, entries 2 and 3). In all the cases the reactions resulted in being regio- and stereoselective, as described for the conversion of **1** into **4a**.

The same panel of diselenides (**3a**–**e**) were reacted with prednisone acetate (**2**), which is the prodrug of prednisolone, a widely used steroidal anti-inflammatory drug [23]. As depicted in Table 2, the reactivity resulted to be very similar to that observed for 1,4-androstadiene-3,17-dione (**1**).

The C1-α-selenenylated derivatives **5a**–**e** were obtained in isolated yields ranging from 52% to 96% (Table 2). Interestingly the ester functionality resulted in being compatible with the applied conditions, and it was not affected by the aqueous acidic conditions.

By using the same protocol, the substrates **1** and **2**, and disulfides **3f**–**h** as chalcogenate sources, the scope of the Thio-Michael **addition** was explored. The results reported in Table 3 were obtained by reducing the commercially available, colorless disulfides **3f**–**h** for 15 min in the zinc-containing, biphasic acidic system [20]. Then, organic and aqueous layers were transferred under argon into a flask containing 1,4-androstadiene-3,17-dione (**1**) or prednisone acetate (**2**), and the resulting mixture was stirred for an additional 3 h at room temperature. As a result of the reduced nucleophilicity of the sulfur atom, thioderivatives **4f** and **5f**–**h** were obtained in lower yields when compared to the selenium analogues, but with the same regio- and stereoselectivity, indicating a lower reactivity of sulfur when compared to selenium in the tested conditions.

## 3. Conclusions

In conclusion, we developed a new methodology for the regio- and stereoselective synthesis of seleno- and thiosteroids using chalcogenating reagents generated in situ by the Zn-mediated reduction of diselenides or disulfides in a biphasic acidic medium. The resulting chalcogen cantered nucleophiles were reacted with model steroids having a Michael acceptor functionalization, affording the target compounds in poor to excellent yields after chromatographic purification.

## 4. Experimental Methods

### 4.1. General Information

Solvent reagents and commercially available starting materials were purchased from Sigma-Aldrich (St. Louis, MO, USA), Alfa Aesar (Kandel, Germany), and VWR (Milano, Italy), and used as received unless otherwise noted. Diselenides **3b**–**e** were synthesized as reported in the literature [22]; the physical and spectral data of **4a**–**f** and **5a**–**h** are reported below and all the spectra are reported in the Supporting Information. Reactions were conducted in round-bottom flasks and were stirred with Teflon coated magnetic stirring bars (Sigma-Aldrich, St. Louis, MO, USA). Flash chromatography was performed with silica gel, pore size 40A (70–230 mesh) unless otherwise stated. All reactions were monitored by TLC on silica gel plates 60 F254 (Merck, Darmstadt, Germany). NMR experiments were performed in a Bruker Advance 400 spectrometer (Bruker, Fällanden, Switzerland). Only selected signals in the ^1^H NMR spectra are reported. The ^1^H and ^13^C NMR chemical shifts (δ) are reported in parts per million (ppm), and they are relative to TMS (0.0 ppm) and the residual solvent peak (CDCl_3_, 7.27 for ^1^H NMR, and 77.0 ppm for ^13^C NMR). The ^77^Se chemical shifts (δ) are reported in parts per million (ppm), and they are relative to diphenyl diselenide (464 ppm) in CDCl_3_. Data are reported as follows: chemical shift, multiplicity, coupling constants, where applicable, and the number of hydrogen atoms. Abbreviations are as follows: s (singlet), d (doublet), t (triplet), q (quartet), dd (doublet of doublet), dt (doublet of triplet), tt (triplet of triplet), m (multiplet), br.s. (broad signal). Coupling constant (J) is quoted in Hz to the nearest 0.1 Hz. High-resolution mass spectrometry (HRMS) measurements were performed using an Agilent 6520 QTOF instrument (Agilent, Santa Clara, CA, USA). IR spectra were obtained in a CHCl_3_ solution with a Thermo Scientific, Nicolet 6700 FT-IR spectrometer (Thermo Scientific, Waltham, MA, USA) and data are reported in reciprocal centimeters. Melting points were determined by a Kofler bench (Boetius type) apparatus and are uncorrected (Wagner & Munz GmbH, Munchen, Germany).

### 4.2. General Procedure for the Michael-Type Addition

Diselenide or disulfide (1.3 equiv.) was added to a flask with 2 mL of 10% HCl, 2 mL of ethyl acetate, then 13 equiv. of zinc shaves (or turnings) were added. The reaction was stirred vigorously (800 rpm) until the discoloration of the organic layer occurred (15–20 min), in the case of colorless disulfides, the reaction was kept for 15 min. Then, the biphasic mixture was separated by the unreacted zinc and transferred under inert conditions (Ar) into a vial containing the steroid 1 or 2 (1 equiv) using the double-ended cannula technique. The reaction mixture was stirred for 3 h at room temperature, poured into water and extracted with ethyl acetate (3 × 20 mL). The organic layer was dried with Na_2_SO_4_, filtered and the solvent removed under vacuum. The products were purified by flash chromatography (Figures S1–S64).

1α-phenylselenylandrost-4-en-3,17-dione (**4a**) [21]

Isolated as a white solid after flash chromatography, eluent petroleum ether/ethyl acetate (7:3). Yield 70%. m.p. (CH_2_Cl_2_/hexane): 172–174 °C [21]: 192.1–193.8 °C); IR,υ_max_ (cm^−1^) 1736, 1677, 1479; ^1^H NMR (400 MHz, CDCl_3_, 298 K, TMS): δ 7.49–7.47 (m, 2H, *o*-C*H*-Ar), 7.24–7.20 (m, 3H, C*H*-Ar), 5.77 (s, 1H, C*H*=C), 3.55 (m, 1H, C*H*-Se), 2.94 (d, 1H, *J* = 17 Hz, C*H*H), 2.60 (d, 1H, *J* = 17 Hz, CH*H*), 1.31 (s, 3H, C*H*_3_), 0.87 (s, 3H, C*H*_3_) ppm; ^13^C NMR (100.6 MHz, CDCl_3_, 298 K, TMS): δ 220.3, 196.4, 166.2, 135.9, 129.4, 128.6, 128.4, 124.7, 51.4, 50.7, 50.2, 47.5, 43.0, 40.7, 35.8, 35.3, 32.4, 31.0, 29.7, 21.8, 19.7, 19.1, 13.8 ppm; ^77^Se NMR (76.3 MHz, CDCl_3_, 298 K, TMS): δ 345.9 ppm. HRMS calculated for C_25_H_31_O_2_Se 443.1484, found 443.1494.

1α-benzylselenylandrost-4-en-3,17-dione (**4b**)

Isolated as a white solid after flash chromatography using petroleum ether/ethyl acetate (7:3); 48% of yield. m.p. (CH_2_Cl_2_/hexane): 153–155 °C; IR,υ_max_ (cm^−1^) 2958, 1736, 1654, 1157; ^1^H NMR (400 MHz, CDCl_3_, 298 K, TMS): δ 7.23–7.14 (m, 5H, C*H*-Ar), 5.69 (s, 1H, C*H*=C), 3.76 (d, 1H, *J* = 12.3Hz), 3.56 (d, 1H, *J* = 12.3 Hz), 3.09 (dd, 1H, *J* = 3.3 and 16.9 Hz), 2.93 (m, 1H, C*H*Se), 2.76 (dd, 1H, *J* = 2.2 and 16.9 Hz), 1.18 (s, 3H, C*H*_3_), 0.77 (s, 3H, CH_3_) ppm; ^13^C NMR (100.6 MHz, CDCl_3_, 298 K, TMS): δ 220.6, 196.6, 167.4, 138.6, 129.0, 128.5, 127.0, 124.6, 50.7, 50.0, 47.5, 45.3, 42.5, 41.2, 35.8, 35.2, 32.3, 31.0, 29.6, 27.1, 21.7, 18.9, 18.6, 13.7 ppm; ^77^Se NMR (76.3 MHz, CDCl_3_, 298 K, TMS): δ 311.6 ppm. HRMS calculated for C_26_H_33_O_2_Se 457.1640, found 457.1652.

1α-phenylethylselenylandrost-4-en-3,17-dione (**4c**)

Isolated as a white solid after flash chromatography using petroleum ether/ethyl acetate (6:4); 51% yield. m.p. (CH_2_Cl_2_/hexane): 163–165 °C; IR,υ_max_ (cm^−1^), 1744, 1243, 1187; ^1^H NMR (400 MHz, CDCl_3_, 298 K, TMS): δ 7.23–7.20 (m, 2H, C*H*-Ar), 7.16–7.10 (m, 3H, C*H*-Ar), 5.71 (s, 1H, C*H*=C), 3.25 (m, 1H, C*H*-Se), 3.07 (dd, 1H, *J* = 3.5 and 17 Hz), 2.95–2.75 (m, 2H), 1.32 (s, 3H, CH_3_), 0.88 (s, 3H, CH_3_) ppm; ^13^C NMR (CDCl_3_, 100.6 MHz, 298 K, TMS): δ 220.4, 196.5, 166.6, 155.4, 140.8, 128.5, 128.4, 127.7, 126.5, 124.5, 50.6, 50.0, 47.5, 45.8, 42.8, 41.0, 36.7, 35.8, 35.3, 32.4, 31.2, 31.0, 25.0, 21.8, 19.6, 18.9, 13.7 ppm; ^77^Se NMR (76.3 MHz, CDCl_3_, 298 K, TMS): δ 215.4 ppm. HRMS calculated for C_27_H_35_O_2_Se 471.1797, found 471.1808.

1α-(4-chlorophenylselenyl)-androst-4-en-3,17-dione (**4d**)

Isolated as a white solid after flash chromatography using petroleum ether/ethyl acetate (6:4); 81% of yield. m.p. (CH_2_Cl_2_/hexane): 175–177 °C; IR,υ_max_ (cm^−1^) 2847, 1738, 1663, 1471; ^1^H NMR (400 MHz, CDCl_3_, 298 K, TMS): δ 7.45 (d, *J* = 8.4 Hz, 2H), 7.24 (d, *J* = 8.4 Hz, 2H), 5.81 (s, 1H), 3.60 (m, 1H), 2.99 (dd, *J* = 3.4 and 17.2 Hz, 1H), 2.61–2.40 (m, 4H), 2.16–2.07 (m, 1H), 2.04–1.30 (m, 11H), 1.24–1.10 (m, 2H), 0.93–0.87 (m, 4H) ppm; ^13^C NMR (CDCl_3_, 100.6 MHz, 298 K, TMS): δ 220.2, 196.1, 166.0, 137.3, 134.9, 129.6, 124.6, 51.6, 50.6, 50.2, 47.4, 43.0, 40.5, 35.8, 35.3, 32.3, 31.0, 29.7, 21.8, 19.7, 19.1, 13.8 ppm; ^77^Se NMR (76.3 MHz, CDCl_3_, 298 K, TMS): δ 339.4 ppm. HRMS calculated for C_22_H_30_ClO_2_Se 477.1094, found 477.1084.

1α-(4-methylophenylselenyl)-androst-4-en-3,17-dione (**4e**)

Isolated as a white solid after flash chromatography using petroleum ether/ethyl acetate (6:4); 64% of yield. m.p. (CH_2_Cl_2_/hexane): 182–184 °C; IR,υ_max_ (cm^−1^) 2920, 1729, 1673, 1187; ^1^H NMR (400 MHz, CDCl_3_, 298 K, TMS): δ 7.43 (d, *J* = 7.9 Hz, 2H), 7.09 (d, *J* = 7.8 Hz, 2H), 5.82 (s, 1H), 3.55 (m, 1H), 2.97 (dd, *J* = 17.2 and 3.5 Hz, 1H), 2.65 (dd, *J* = 17.1 and 2.5 Hz, 1H), 2.33 (s, 3H, CH3), 1.36 (s, 3H, CH3), 0.93 (s, 3H, CH3) ppm; ^13^C NMR (CDCl_3_, 100.6 MHz, 298 K, TMS): δ 220.2, 196.4, 166.1, 138.4, 136.1, 130.1, 124.8, 124.6, 51.4, 50.6, 50.0, 47.4, 42.9, 40.5, 35.7, 35.3, 32.3, 30.9, 29.6, 21.7, 21.2, 19.6, 19.0, 13.7 ppm; ^77^Se NMR (76.3 MHz, CDCl_3_, 298 K, TMS): δ 335.7 ppm. HRMS calculated for C_26_H_33_O_2_Se 457.1640, found 457.1627.

1α-phenyltioandrost-4-en-3,17-dione (**4f**) [21]

Isolated as a white solid after flash chromatography using petroleum ether/ethyl acetate (6:4); 35% of yield. m.p. (CH_2_Cl_2_/hexane): 186–190 °C (ref^21^ 188.1–189.3 °C); IR,υ_max_ (cm^−1^): 1740, 1685, 1613, 1475. ^1^H NMR (400 MHz, CDCl_3_, 298 K, TMS): δ 7.41–7.39 (m, 2H), 7.31–7.27 (m, 3H), 5.84 (s, 1H, C*H*=C), 3.55 (m, 1H, C*H*-S), 2.77 (dd, 1H, *J* = 3.0 and 16.9 Hz), 1.38 (s, 3H, CH_3_), 0.94 (s, 3H, CH_3_) ppm; ^13^C NMR (CDCl_3_, 100.6 MHz, 298 K, TMS): δ 220.4, 196.2, 165.7, 133.9, 133.7, 129.3, 128.0, 124.6, 54.4, 50.7, 47.9, 47.5, 42.7, 39.7, 35.8, 35.2, 32.4, 31.0, 29.7, 21.8, 19.9, 19.6, 13.8 ppm. HRMS calculated for C_25_H_31_O_2_S 395.2039, found 395.2056.

1α-phenylselenyl-17,21-dihydroxy-pregn-4-eno-3,12,20–trioxo–21–acetate (**5a**)

Isolated as a white solid after flash chromatography, eluent petroleum ether/ethyl acetate (6:4); 70% of yield. m.p. (CH_2_Cl_2_/hexane): 204–206 °C; IR,υ_max_ (cm^−1^) 2973, 1692, 1654, 1433; ^1^H NMR (400 MHz, CDCl_3_, 298 K, TMS): δ 7.42–7.40 (m, 2H, C*H*-Ar), 7.22–7.18 (m, 3H, C*H*-Ar), 5.72 (s, 1H, C*H*=C), 5.04 (d, 1H, *J* = 17.6 Hz), 4.66 (d, 1H, *J* = 17.6 Hz), 4.45–4.44 (m, 1H, C*H*Se), 3.10–2.90 (m, 2H), 2.80–2.20 (m, 8H), 2.10 (s, 3H, C*H*_3_), 1.95–1.55 (m, 5H), 1.48 (s, 3H, C*H*_3_), 1.45–1.20 (m, 3H), 0.62 (s, 3H, C*H*_3_) ppm; ^13^C NMR (100.6 MHz, CDCl_3_, 298 K, TMS): δ 208.9, 204.6, 197.2, 170.6, 165.1, 135.4, 129.2, 128.5, 128.1, 124.8, 89.0, 67.7, 60.2, 51.8, 51.3, 49.6, 49.4, 42.7, 40.7, 36.8, 35.0, 32.0, 31.6, 23.2, 20.5, 18.4, 15.5 ppm. HRMS calcd for C_29_H_35_O_6_Se 559.1593, found 559.1599.

1α-benzylselenyl-17,21-dihydroxy-pregn-4-eno-3,12,20-trioxo-21-acetate (**5b**)

Isolated as a white solid, eluent petroleum ether/ethyl acetate (6:4); 52% of yield. m.p. (CH_2_Cl_2_/hexane): 175–177 °C; IR,υ_max_ (cm^−1^) 2853, 1751, 1695, 1598; ^1^H NMR (400 MHz, CDCl_3_, 298 K, TMS): δ 7.29–7.22 (m, 5H, C*H*-Ar), 5.74 (s, 1H, C*H*=C), 5.12 (d, 1H, *J* = 17.7 Hz), 4.71 (d, 1H, *J* = 17.6 Hz), 4.18 (m, 1H, C*H*Se), 3.75 (d, 1H, *J* = 11.5 Hz), 3.65 (d, 1H, *J* = 11.5 Hz), 2.20 (s, 3H, CH_3_), 1.52 (s, 3H, CH_3_), 0.67 (s, 3H, CH_3_) ppm. ^13^C-NMR (CDCl_3_, 100.6 MHz, 298 K, TMS): δ 209.1, 204.6, 197.2, 170.7, 166.1, 138.3, 128.9, 128.6, 126.9, 124.9, 89.1, 67.7, 60.0, 59.9, 51.5, 51.2, 49.6, 49.4, 47.0, 42.7, 42.4, 41.9, 36.8, 36.1, 32.6, 31.5, 28.8, 23.3, 20.6, 18.4, 15.5 ppm; ^77^Se NMR (76.3 MHz, CDCl_3_, 298 K, TMS): δ 295.7 ppm. HRMS calculated for C_30_H_37_O_6_Se 573.1750, found 573.1758.

*1α-phenylethylselenyl-17,21-dihydroxy-pregn-4-eno-3,12,20-trioxo-21-acetate* (**5c**)

Isolated as a white solid after flash chromatography using petroleum ether/ethyl acetate (6:4); 69% yield. m.p. (CH_2_Cl_2_/hexane): 203–205 °C; ^1^H NMR (400 MHz, CDCl_3_, 298 K, TMS): 7.31–7.29 (m, 2H, C*H*-Ar), 7.22–7.16 (m, 3H, C*H*-Ar), 5.74 (s 1H C*H*=C), 5.12 (d, 1H, *J* = 17.5 Hz), 4.71 (d, 1H, *J* = 17.5 Hz), 4.26 (m, 1H, C*H*Se), 3.27 (dd 1H *J* = 3.8 and 13.2 Hz), 2.17 (s 3H), 0.68 (s 3H); ^13^C-NMR (CDCl_3_, 100.6 MHz, 298 K, TMS): δ 209.2, 204.4, 196.5, 170.5, 165.4, 140.9, 128.4, 126.3, 124.9, 89.0, 67.5, 60.0, 51.2, 49.6, 49.5, 46.7, 42.8, 42.0, 36.8, 36.7, 35.1, 32.1, 31.4, 29.7, 26.1, 23.3, 20.4, 18.4, 15.5; ^77^Se NMR (76.3 MHz, CDCl_3_, 298 K, TMS): δ 222.7; HRMS calculated for C_30_H_37_O_6_Se 587.1906, found 587.1910.

*1α-(4-chlorophenylselenyl)-17,21-dihydroxy-pregn-4-eno-3,12,20-trioxo-21-acetate* (**5d**)

Isolated as a white solid after flash chromatography using petroleum ether/ethyl acetate (6:4); 69% yield. m.p. (CH_2_Cl_2_/hexane): 207–209 °C; IR,υ_max_ (cm^−1^) 2950, 1751, 1662, 1467; ^1^H NMR (400 MHz, CDCl_3_, 298 K, TMS): δ 7.40 (d, *J* = 8.4 Hz, 2H, C*H*-Ar), 7.22 (d, *J* = 8.4 Hz, 2H, C*H*-Ar), 5.78 (s, 1H, C*H*=C), 5.10 (d, *J* = 17.6 Hz, 1H), 4.74 (d, *J* = 17.6 Hz, 1H), 4.50 (m, 1H, C*H*Se), 3.50 (br s, 1H, OH), 3.10 (dd, *J* = 3.6 and 17.2 Hz, 1H), 2.17 (s, 3H, CH_3_), 1.54 (s, 3H, CH_3_), 0.67 (s, 3H, CH_3_) ppm; ^13^C-NMR (CDCl_3_, 100.6 MHz, 298 K, TMS): δ 209.2, 204.7, 197.3, 170.7, 165.2, 136.7, 134.6, 129.4, 126.6, 124.7, 88.9, 67.8, 60.2, 52.2, 51.2, 49.6, 49.4, 42.7, 40.6, 36.8, 34.9, 32.0, 31.6, 23.2, 20.5, 18.3, 15.4 ppm; ^77^Se NMR (76.3 MHz, CDCl_3_, 298 K, TMS): δ 339.2 ppm. HRMS calculated for C_28_H_34_ClO_6_Se 593.1204, found 593.1183.

*1α-(4-methylophenylselenyl)-17,21-dihydroxy-pregn-4-eno-3,12,20–trioxo-21-acetate* (**5e**)

Isolated as a white solid after flash chromatography using petroleum ether/ethyl acetate (8:2); 96% of yield. m.p. (CH_2_Cl_2_/hexane): 188–190 °C; IR,υ_max_ (cm^−1^) 2923, 1738, 1663, 1621; ^1^H NMR (400 MHz, CDCl_3_, 298 K, TMS): δ 7.36 (d, *J* = 7.8 Hz, 2H), 7.06 (d, *J* = 7.8 Hz, 2H), 5.78 (s, 1H), 4.75 (d, *J* = 17.6 Hz, 1H), 4.44 (m, 1H), 2.31 (s, 3H, CH_3_), 2.17 (s, 3H, CH_3_), 1.53 (s, 3H, CH_3_), 0.68 (s, 3H, CH_3_) ppm; ^13^C-NMR (CDCl_3_, 100.6 MHz, 298 K, TMS): δ 209.0, 204.7, 197.6, 170.6, 165.3, 138.2, 135.6, 130.0, 124.8, 124.7, 89.0, 67.8, 60.2, 51.7, 51.3, 49.6, 49.5, 42.7, 40.6, 36.8, 34.9, 32.0, 31.6, 23.2, 21.1, 20.5, 18.4, 15.4 ppm; ^77^Se NMR (76.3 MHz, CDCl_3_, 298 K, TMS): δ 333.6 ppm. HRMS calculated for C_30_H_37_O_6_Se 573.1750, found 573.1731.

*1α-phenyltio-17,21-dihydroxy-pregn-4-eno-3,12,20-trioxo-21-acetate* (**5f**)

Isolated as a white solid after flash chromatography, using petroleum ether/ethyl acetate (8:2); 33% of yield. m.p. (CH_2_Cl_2_/hexane): 220–221 °C; IR,υ_max_ (cm^−1^): 1744, 1695, 1613, 1221; ^1^H NMR (400 MHz, CDCl_3_, 298 K, TMS): δ 7.36–7.26 (m, 5H), 5.80 (s, 1H, C*H*=C), 5.11 (d, 1H, *J* = 17.6 Hz), 4.74 (d, 1H, *J* = 17.6 Hz), 4.43 (t, 1H, *J* = 2.8 Hz), 3.33 (br s, 1H), 3.03–2.98 (m, 2H), 2.87–2.78 (m, 2H), 2.17 (s, 3H, C*H*_3_), 1.55 (s, 3H, C*H*_3_), 0.67 (s, 3H, C*H*_3_) ppm; ^13^C NMR (CDCl_3_, 100.6 MHz, 298 K, TMS): δ 209.4, 204.8, 197.5, 170.8, 164.8, 133.8, 133.6, 129.2, 127.9, 124.9, 89.11, 67.9, 57.9, 54.6, 51.4, 49.7, 49.5, 42.4, 39.6, 36.7, 35.1, 32.2, 31.6, 23.3, 20.6, 19.2, 15.5 ppm. HRMS calculated for C_29_H_35_O_6_S 511.2149, found 511.2169.

*1α-benzyltio-17,21-dihydroxy-pregn-4-eno-3,12,20-trioxo-21-acetate* (**5g**)

Isolated as a white solid after flash chromatography, using petroleum ether/ethyl acetate (8:2); 11% of yield. m.p. (CH_2_Cl_2_/hexane): 192–194 °C; IR,υ_max_ (cm^−1^): 2920, 2845, 1695, 1650; ^1^H NMR (400 MHz, CDCl_3_, 298 K, TMS): δ 7.32–7.22 (m, 5H, C*H*-Ar), 5.74 (s, 1H, C*H*=C), 5.14 (d, 1H, *J* = 17.5 Hz), 4.71 (d, 1H, *J* = 17.5 Hz), 3.96 (t, 1H, *J* = 2 Hz), 3.65 (d, 1H, *J* = 12.9 Hz), 3.54 (d, 1H, *J* = 12.9 Hz), 2.19 (s, 3H, C*H*_3_), 1.49 (s, 3H, C*H*_3_), 0.65 (s, 3H, C*H*_3_) ppm; ^13^C NMR (CDCl_3_, 100.6 MHz, 298 K, TMS): δ 209.1, 204.5, 197.1, 170.7, 165.2, 137.4, 129.0, 128.6, 127.2, 124.9, 89.1, 67.6, 57.5, 49.6, 49.3, 42.4, 40.5, 36.9, 36.6, 35.2, 32.2, 23.3, 20.6, 19.3, 15.5 ppm. HRMS calculated for C_30_H_37_O_6_S 525.2305, found 525.2329.

*1α-(4-methylophenyltio-17,21-dihydroxy-pregn-4-eno-3,12,20-trioxo-21-acetate* (**5h**)

Isolated as a white solid after flash chromatography, using petroleum ether/ethyl acetate (8:2); 33% of yield. m.p. (CH_2_Cl_2_/hexane): 193–195 °C; IR,υ_max_ (cm^−1^) 2943, 1748, 1658, 1217; ^1^H NMR (400 MHz, CDCl_3_, 298 K, TMS): δ 7.23 (d, 2H, *J* = 8.0 Hz), 7.07 (d, 2H, *J* = 8.0 Hz), 5.79 (s, 1H, C*H*=C), 5.11 (d, 1H, *J* = 17.6 Hz), 4.74 (d, 1H, *J* = 17.6 Hz), 4.33 (t, 1H, *J* = 3.2 Hz, C*H*-S), 3.33 (s, 1H), 3.04 (d, 1H, *J* = 12.6 Hz), 2.99 (d, 1H, 11.2 Hz), 2.83–2.78 (m, 2H), 2.30 (s, 3H, CH_3_), 2.17 (s, 3H, CH_3_), 1.53 (s, 3H, CH_3_), 0.68 (s, 3H, CH_3_) ppm; ^13^C NMR (CDCl_3_, 100.6 MHz, 298 K, TMS): δ 209.4, 204.8, 197.6, 170.7, 164.7, 138.2, 134.2, 129.9, 124.9, 89.1, 67.9, 57.9, 54.8, 51.4, 49.7, 49.5, 42.4, 39.5, 36.7, 35.1, 32.2, 31.6, 23.3, 21.2, 20.6, 19.2, 15.5 ppm. HRMS calculated for C_30_H_37_O_6_S 525.2305, found 525.2328.

## Figures and Tables

**Table 1:** Seleno-Michael reactions on substrate (**1**).

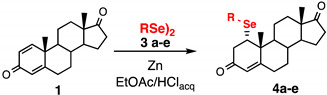			
Entry	(RSe)_2_ (3)	Product (4)	Yield %
1	**3a**	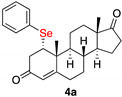	70
2	**3b**	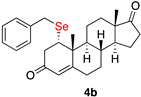	48
3	**3c**	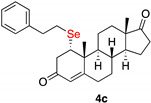	51
4	**3d**	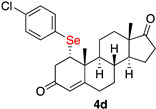	81
5	**3e**	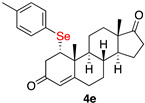	64

**Table 2:** Seleno-Michael reactions on substrate (**2**).

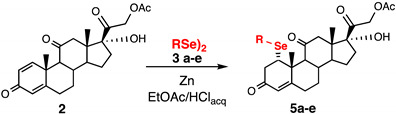			
Entry	(RSe)_2_ (3)	Product (5)	Yield %
1	**3a**	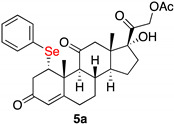	70
2	**3b**	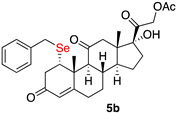	52
3	**3c**	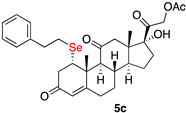	69
4	**3d**	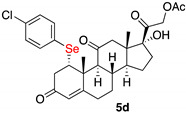	69
5	**3e**	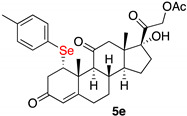	96

**Table 3:** Thio-Michael reactions.

Entry	Substrate	(RS)_2_ (3)	Product (4 from 1 or 5 from 2)	Yield %
1	**1**	**3f**	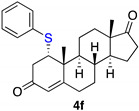	35
2	**2**	**3f**	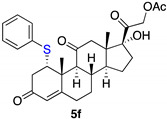	33
3	**2**	**3g**	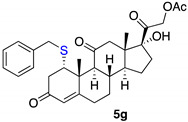	11
4	**2**	**3h**	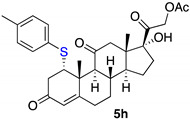	33

## Data Availability

All the data are available within the manuscript and its Supporting Information.

## Supplementary Materials

The following supporting information can be downloaded at: https://www.mdpi.com/article/10.3390/ijms23063022/s1, Spectroscopic data of all the synthetized compounds.

## References

[1] Pacuła A.J., Mangiavacchi F., Sancineto L.J., Lenardão E., Ścianowski J., Santi C. (2016). An Update on “Selenium Containing Compounds from Poison to Drug Candidates: A Review on the GPx-like Activity”. Curr. Chem. Biol..

[2] Nogueira C.W., Zeni G., Rocha J.B.T. (2004). Organoselenium and Organotellurium Compounds: Toxicology and Pharmacology. Chem. Rev..

[3] Mangiavacchi F., Botwina P., Menichetti E., Bagnoli L., Rosati O., Marini F., Fonseca S.F., Abenante L., Alves D., Dabrowska A. (2021). Seleno-Functionalization of Quercetin Improves the Non-Covalent Inhibition of Mpro and Its Antiviral Activity in Cells against SARS-CoV-2. IJMS.

[4] Takafuta T., Fujimura K. (2017). Steroids. Autoimmune Thrombocytopenia.

[5] Jastrzebska I., Grzes P.A., Niemirowicz-Laskowska K., Car H. (2021). Selenosteroids—Promising Hybrid Compounds with Pleiotropic Biological Activity: Synthesis and Biological Aspects. J. Steroid Biochem. Mol. Biol..

[6] Romero-Hernández L.L., Merino-Montiel P., Montiel-Smith S., Meza-Reyes S., Vega-Báez J.L., Abasolo I., Schwartz S., López Ó., Fernández-Bolaños J.G. (2015). Diosgenin-based thio(seleno)ureas and triazolyl glycoconjugates as hybrid drugs. Antioxidant and antiproliferative profile. Eur. J. Med. Chem..

[7] Fuentes-Aguilar A., Romero-Hernández L.L., Arenas-González A., Merino-Montiel P., Montiel-Smith S., Meza-Reyes S., Vega-Báez J.L., Plata G.B., Padrón J.M., López Ó. (2017). New selenosteroids as antiproliferative agents. Org. Biomol. Chem..

[8] Cui J., Pang L., Wei M., Gan C., Liu D., Yuan H., Huang Y. (2018). Synthesis and antiproliferative activity of 17-[1′,2′,3′]-selenadiazolylpregnenolone compounds. Steroids.

[9] Ibrahim-Ouali M. (2009). First total synthesis of 11-selena steroids. Tetrahedron Lett..

[10] Barton D.H.R., Bashiardes G., Fourrey J.-L. (1984). A new synthesis of phenylvinylselenides. Tetrahedron Lett..

[11] Rodrigues O.E.D., de Souza D., Soares L.C., Dornelles L., Burrow R.A., Appelt H.R., Alves C.F., Alves D., Braga A.L. (2010). Stereoselective synthesis of selenosteroids. Tetrahedron Lett..

[12] Jastrzebska I., Mellea S., Salerno V., Grzes P.A., Siergiejczyk L., Niemirowicz-Laskowska K., Bucki R., Monti B., Santi C. (2019). Santi PhSeZnCl in the Synthesis of Steroidal β-Hydroxy-Phenylselenides Having Antibacterial Activity. Int. J. Mol. Sci..

[13] Santi C., Capoccia L., Monti B. (2018). Zinc-Selenium reagents in organic synthesis. Phys. Sci. Rev..

[14] Santi C., Santoro S., Testaferri L., Tiecco M. (2008). A Simple Zinc-Mediated Preparation of Selenols. Synlett.

[15] Salman S., Schwab R., Alberto E., Vargas J., Dornelles L., Rodrigues O., Braga A. (2011). Efficient Ring Opening of Protected and Unprotected Aziridines Promoted by Stable Zinc Selenolate in Ionic Liquid. Synlett.

[16] Bellino G., Scisciani M., Vargas J.P., Sancineto L., Bagnoli L., Marini F., Lüdtke D.S., Lenardao E.J., Santi C. (2016). Reaction of Acyl Chlorides with in Situ Formed Zinc Selenolates: Synthesis of Selenoesters versus Ring-Opening Reaction of Tetrahydrofuran. J. Chem..

[17] Flemer S. (2014). A comprehensive one-pot synthesis of protected cysteine and selenocysteine SPPS derivatives. Protein Pept. Lett..

[18] Flemer S. (2015). Fmoc-Sec(Xan)-OH: Synthesis and utility of Fmoc selenocysteine SPPS derivatives with acid-labile sidechain protection. J. Pept. Sci..

[19] Tidei C., Sancineto L., Bagnoli L., Battistelli B., Marini F., Santi C. (2014). A Recyclable Biphasic System for Stereoselective and Easily Handled Hydrochalcogenations. Eur. J. Org. Chem..

[20] Nacca F.G., Monti B., Lenardão E.J., Evans P., Santi C. (2020). A Simple Zinc-Mediated Method for Selenium Addition to Michael Acceptors. Molecules.

[21] Berthonneau C., Nun P., Rivière M., Pauvert M., Dénès F., Lebreton J. (2018). Hemisynthesis of 2,3,4- 13 C3-1,4-Androstadien-3,17-dione: A Key Precursor for the Synthesis of 13 C3-Androstanes and 13 C3-Estranes. J. Org. Chem..

[22] Bhasin K., Singh J. (2002). A novel and convenient synthesis towards 2-pyridylselenium compounds: X-ray crystal structure of 4,4′-dimethyl-2,2′-dipyridyl diselenide and tris(2-pyridylseleno)methane. J. Organomet. Chem..

[23] Lu J., Cheng P., Zhang Y., Jia X., Zhang N. (2019). The effect of prednisone acetate combined with cyclophosphamide on systemic lupus erythematosus and serum il-4, il-6, and il-10 expressions. Int. J. Clin. Exp. Med..

