# Supplementary material for: Simple Zn-Mediated Seleno- and Thio-Functionalization of Steroids at C-1 Position
Source: Int J Mol Sci. 2022 Mar 11;23(6):3022. doi: 10.3390/ijms23063022 (PMC8952209; doi:10.3390/ijms23063022)

Supporting Information

Simple Zn-Mediated Seleno- and Thio-Functionalization of Steroids at C-1 Position

Paweł A. Grześ,[a,b] Bonifacio Monti,[b] Natalia Wawrusiewicz-Kurylonek,[c] Luana Bagnoli,[b] Luca Sancineto,[b] Izabella Jastrzebska[a*] and Claudio Santi*[b]

a. Department of Chemistry, University of Białystok, ul. Ciołkowskiego 1K, 15-245 Białystok, Poland.

b. Department of Pharmaceutical Sciences, Group of Catalysis, Synthesis and Organic Green Chemistry, University of Perugia, Via del Liceo 1, 06132 Perugia, Italy.

c. Department of Clinical Genetics, Department of Endocrinology, Diabetology and Internal Medicine, Medical University of Białystok, Skłodowska – Curie 24A, 15-276, Białystok, Poland.

Copies of ^{1}H, ^{13}C, ^{77}Se, IR spectra and HRMS analysis for compounds **4a-f** and **5a-h** S2

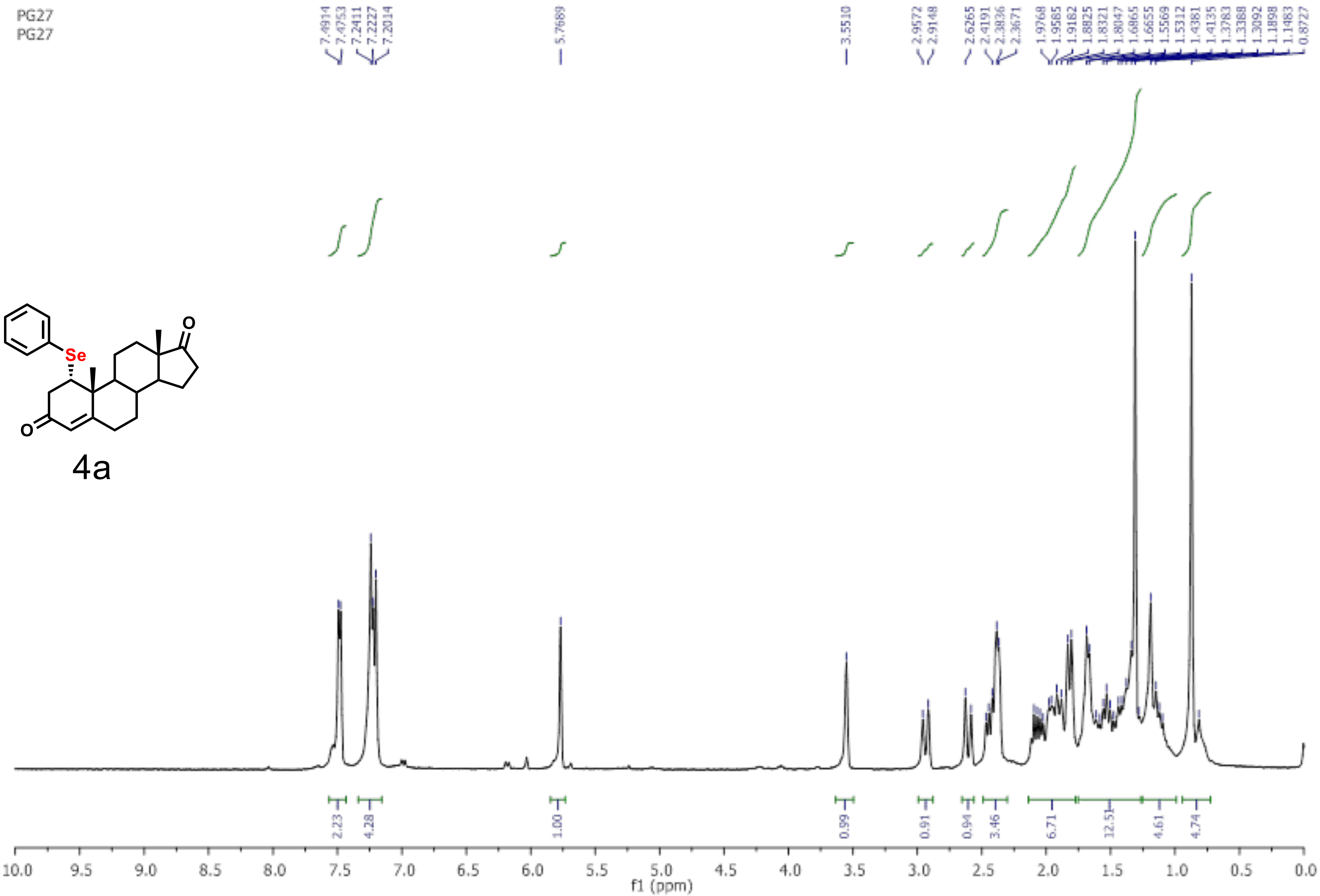
PG27
PG27
7.4914
7.4753
7.2411
7.2227
7.2014
5.7689
3.5510
2.9572
2.9148
2.6265
2.4191
2.3836
2.3671
1.9768
1.9585
1.9182
1.8825
1.8321
1.8047
1.6865
1.6655
1.5569
1.5312
1.4381
1.4135
1.3783
1.3388
1.3092
1.1898
1.1483
0.8727
2.23
4.28
1.00
0.99
0.91
0.94
3.46
6.71
12.51
4.61
4.74
10.0 9.5 9.0 8.5 8.0 7.5 7.0 6.5 6.0 5.5 5.0 4.5 4.0 3.5 3.0 2.5 2.0 1.5 1.0 0.5 0.0
f1 (ppm)
Se
O
O

4a

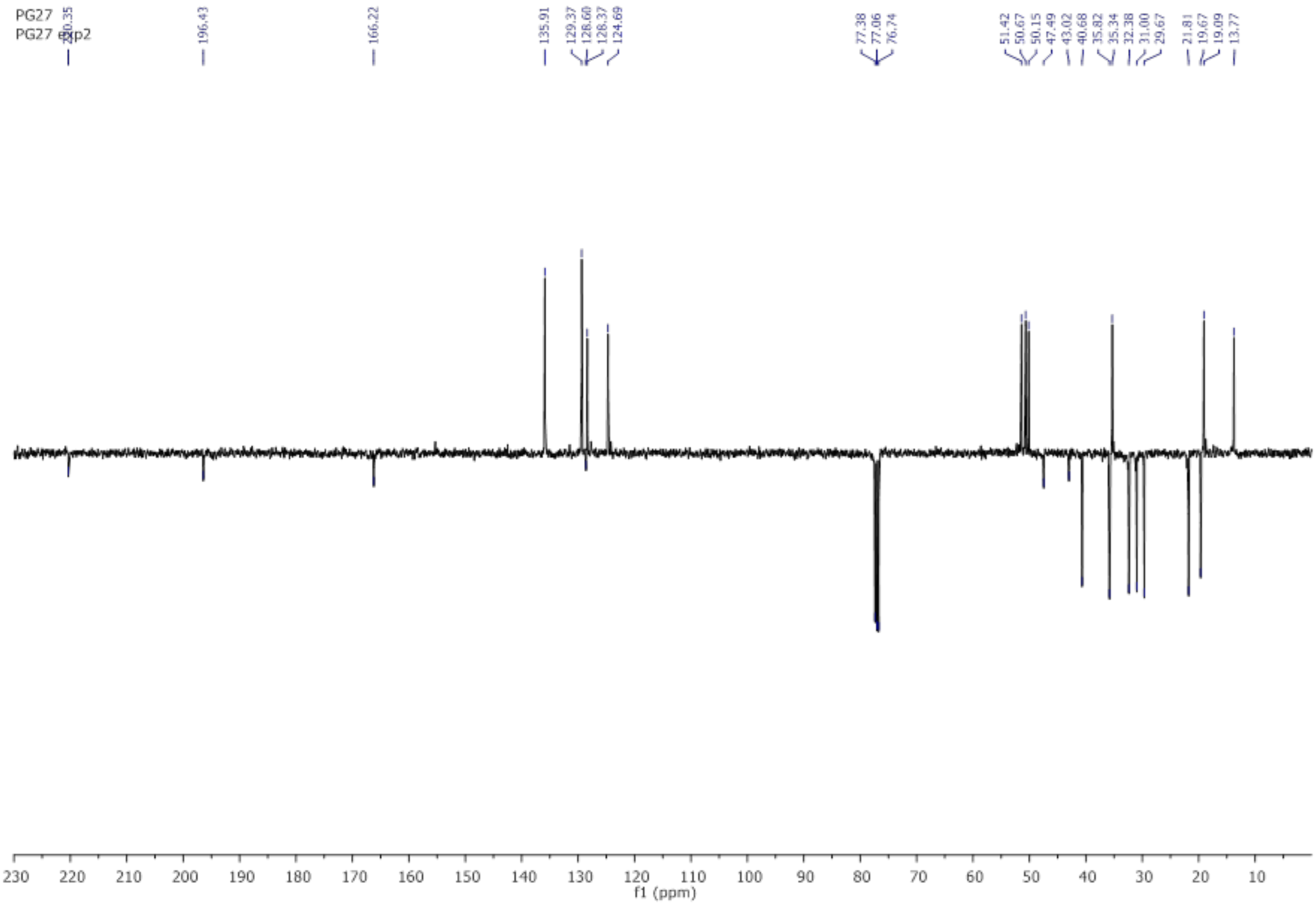
PG27
PG27 exp2
220.35
196.43
166.22
135.91
129.37
128.60
128.37
124.69
77.38
77.06
76.74
51.42
50.67
50.15
47.49
43.02
40.68
35.82
35.34
32.38
31.00
29.67
21.81
19.67
19.09
13.77
230 220 210 200 190 180 170 160 150 140 130 120 110 100 90 80 70 60 50 40 30 20 10
f1 (ppm)

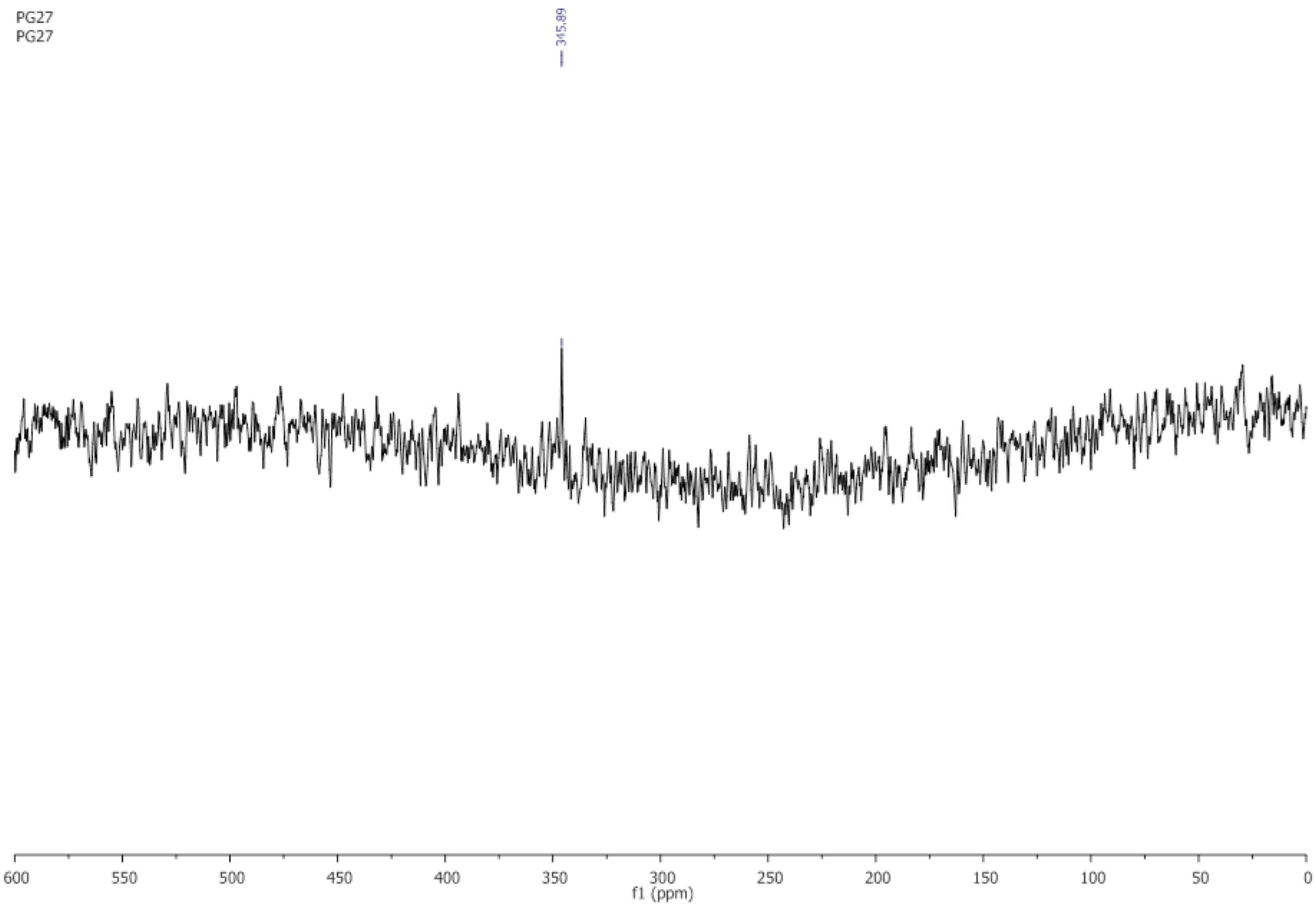
PG27
PG27
345.89
600 550 500 450 400 350 300 250 200 150 100 50 0
f1 (ppm)

**Sample Name**	PG26	**Position**	P1-B1	**Instrument Name**	Instrument 1
**User Name**		**Inj Vol**	0.1	**InjPosition**	
**Sample Type**	Sample	**IRM Calibration Status**	Success	**Data Filename**	6min_MS 2-6minMeOH.m PG26 V=0.1.d
**ACQ Method**	6min_MS 2-6minMeOH.m	**Comment**		**Acquired Time**	7/8/2019 5:40:06 PM

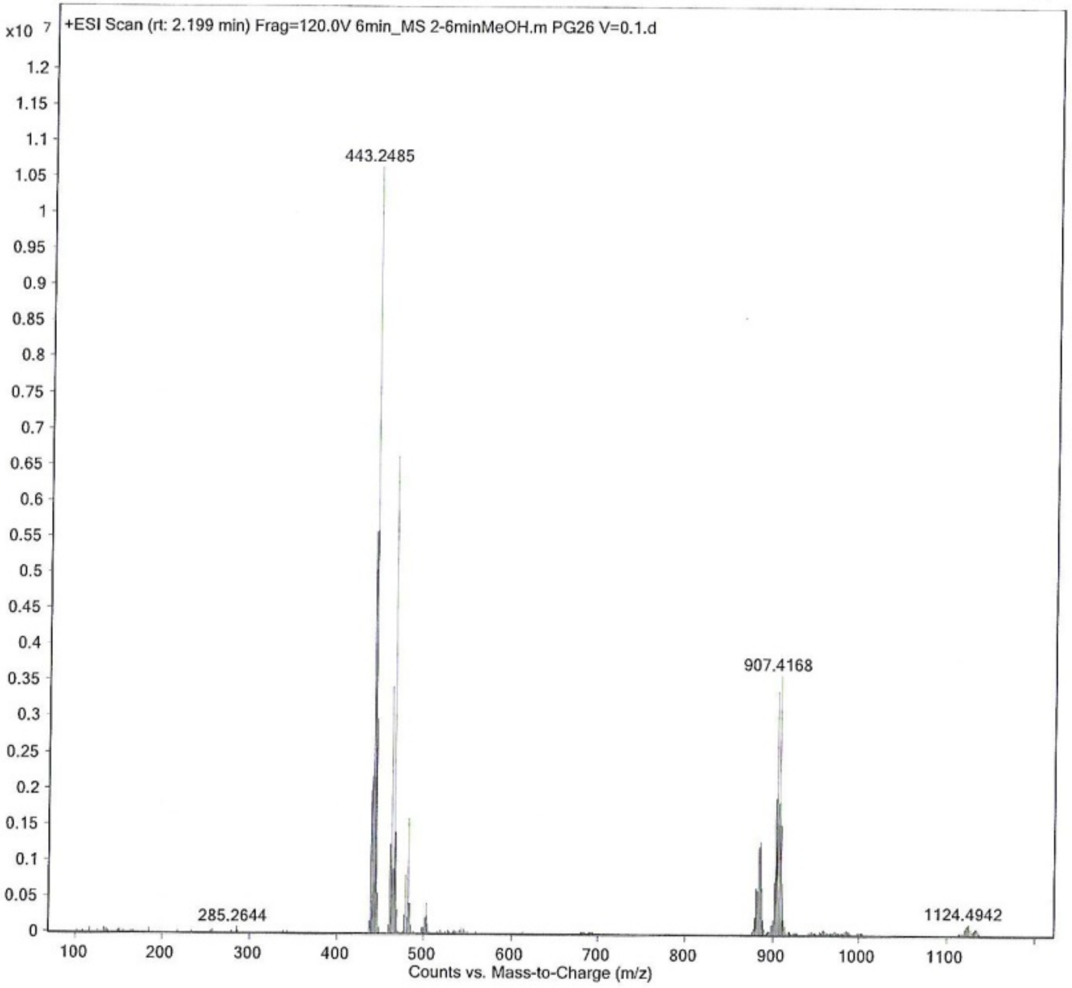

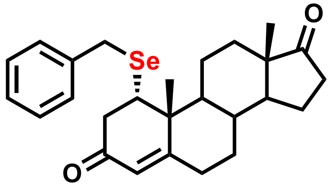

4b

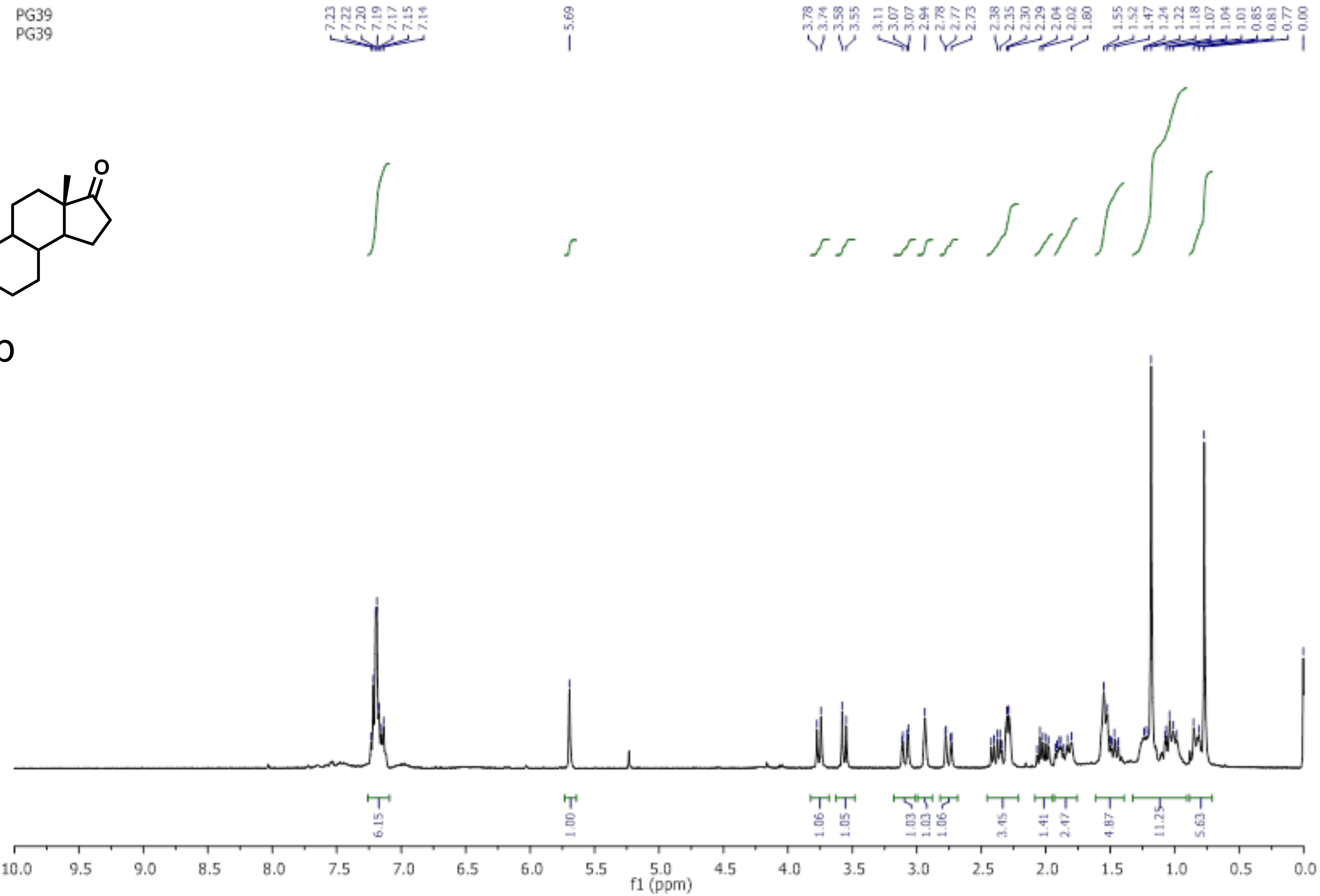

PG39
PG39
7.23
7.22
7.20
7.19
7.17
7.15
7.14
5.69
3.78
3.74
3.58
3.55
3.11
3.07
3.07
2.94
2.78
2.77
2.73
2.38
2.35
2.30
2.29
2.04
2.02
1.80
1.55
1.52
1.47
1.24
1.22
1.18
1.07
1.04
1.01
0.85
0.81
0.77
0.00
6.15
1.00
1.06
1.05
1.03
1.03
1.06
3.45
1.41
2.47
4.87
11.25
5.63
10.0
9.5
9.0
8.5
8.0
7.5
7.0
6.5
6.0
5.5
5.0
4.5
4.0
3.5
3.0
2.5
2.0
1.5
1.0
0.5
0.0
f1 (ppm)

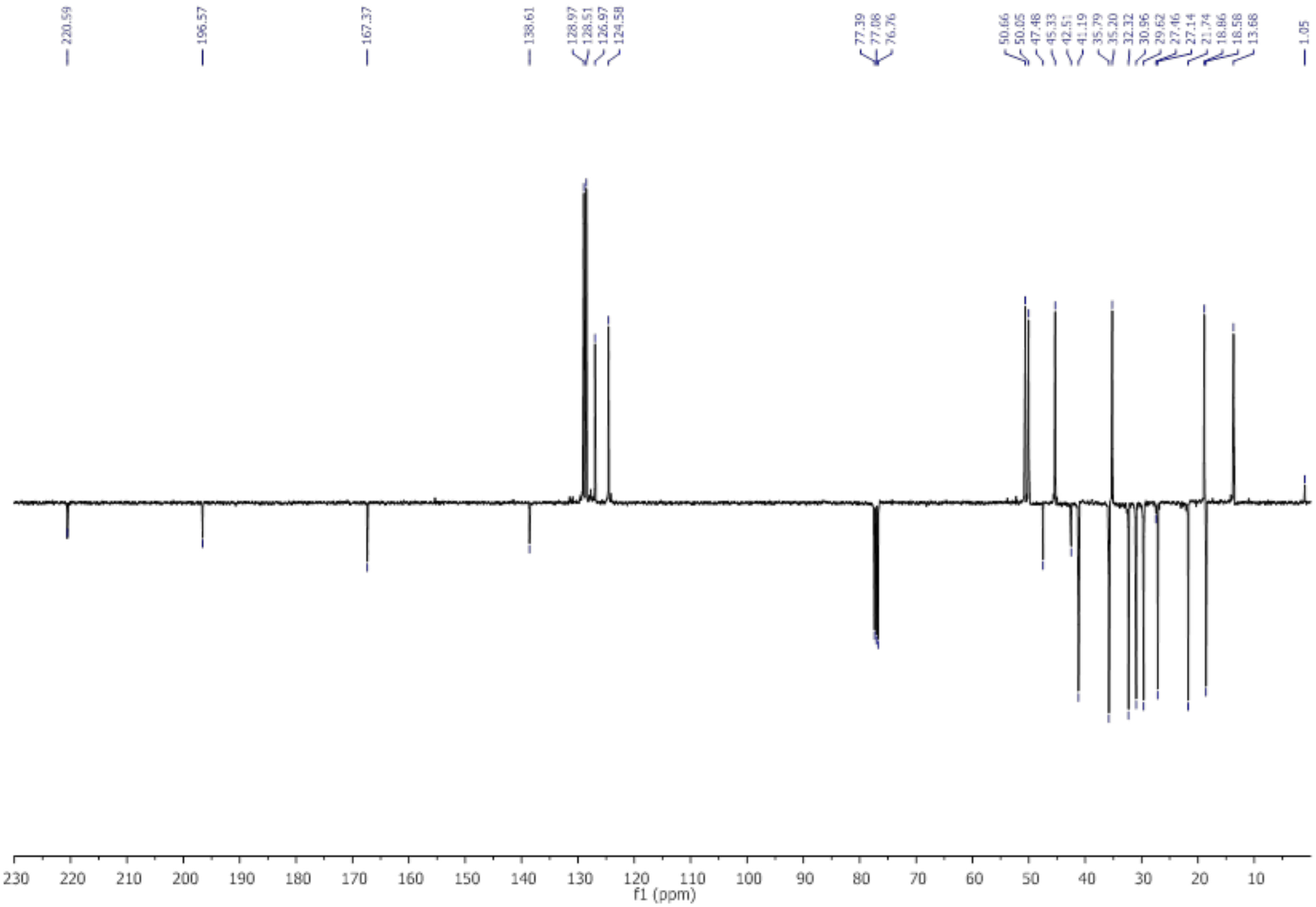
220.59
196.57
167.37
138.61
128.97
128.51
126.97
124.58
77.39
77.08
76.76
50.66
50.05
47.48
45.33
42.51
41.19
35.79
35.20
32.32
30.96
29.62
27.46
27.14
21.74
18.86
18.58
13.68
1.05
230 220 210 200 190 180 170 160 150 140 130 120 110 100 90 80 70 60 50 40 30 20 10
f1 (ppm)

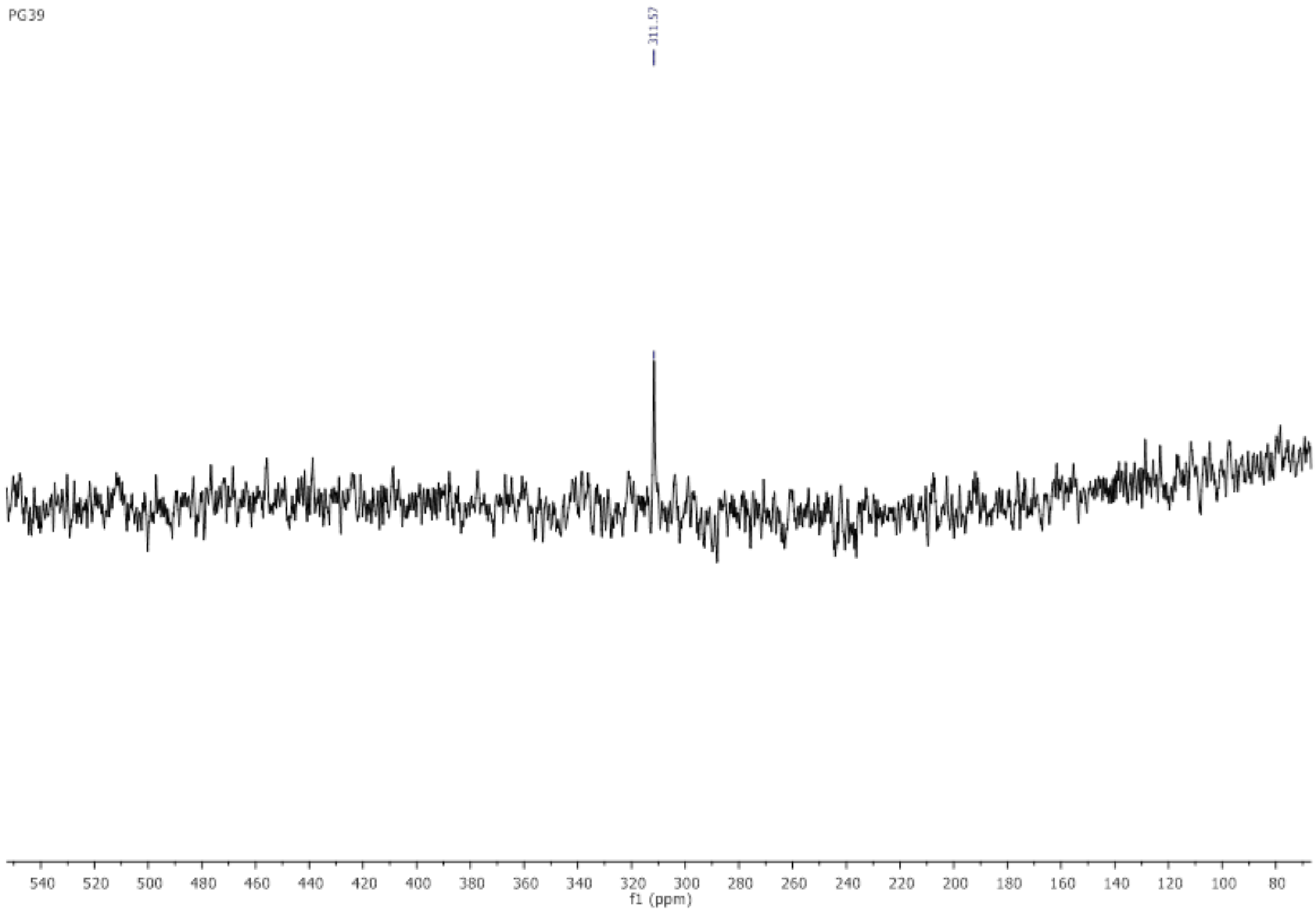
PG39
311.57
540 520 500 480 460 440 420 400 380 360 340 320 300 280 260 240 220 200 180 160 140 120 100 80
f1 (ppm)

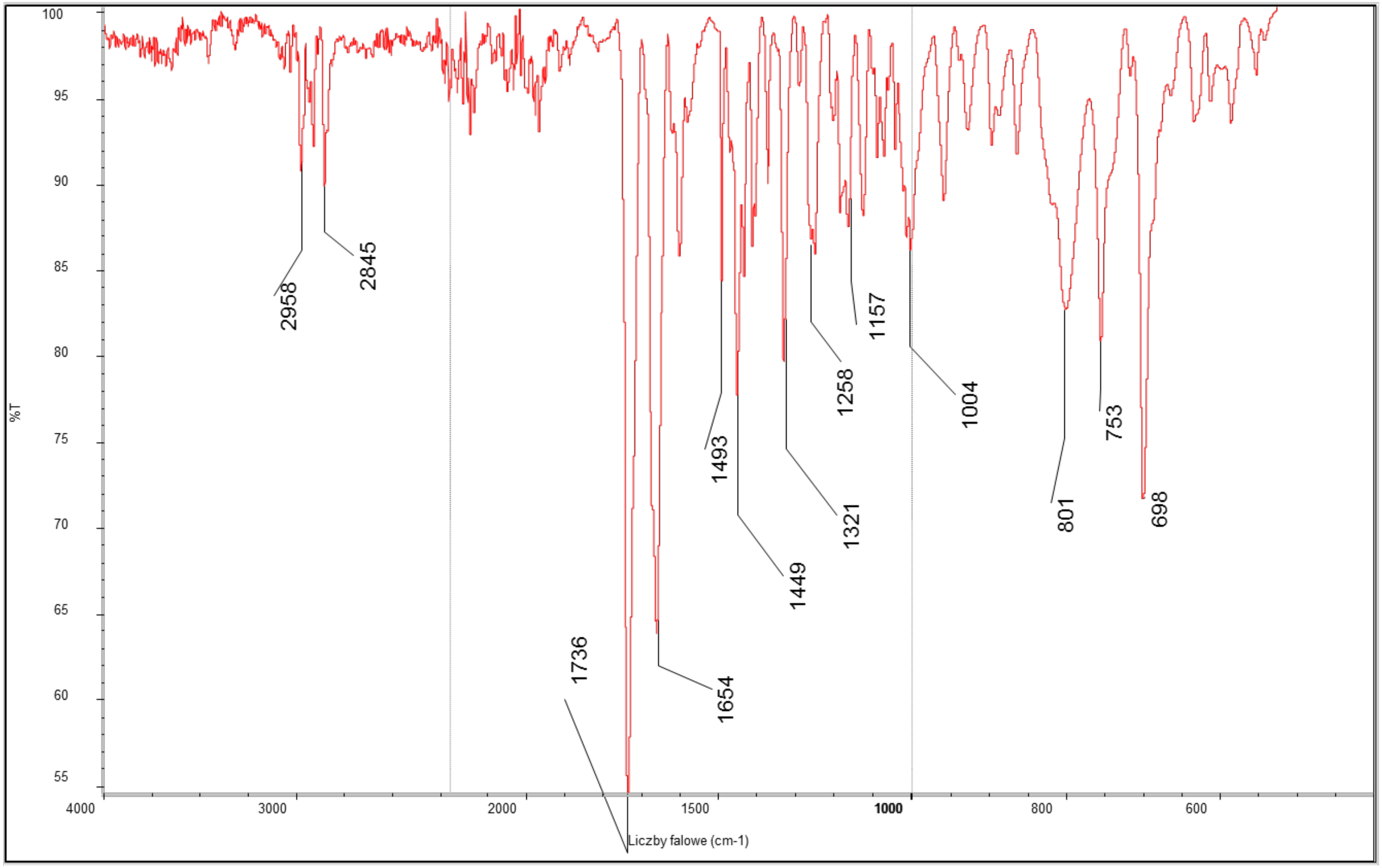

100
95
90
85
80
75
70
65
60
55
%T
2958
2845
1736
1654
1493
1449
1321
1258
1157
1004
801
753
698
4000
3000
2000
1500
1000
800
600
Liczby falowe (cm-1)

**Sample Name**	Unavailable	**Position**	Unavailable	**Instrument Name**	Unavailable
**User Name**	Unavailable	**Inj Vol**	Unavailable	**InjPosition**	Unavailable
**Sample Type**	Unavailable	**IRM Calibration Status**	Success	**Data Filename**	6min_MS 2-6minMeOH.m PG35 V=0.1.d
**ACQ Method**		**Comment**	Sample information is unavailable	**Acquired Time**	Unavailable

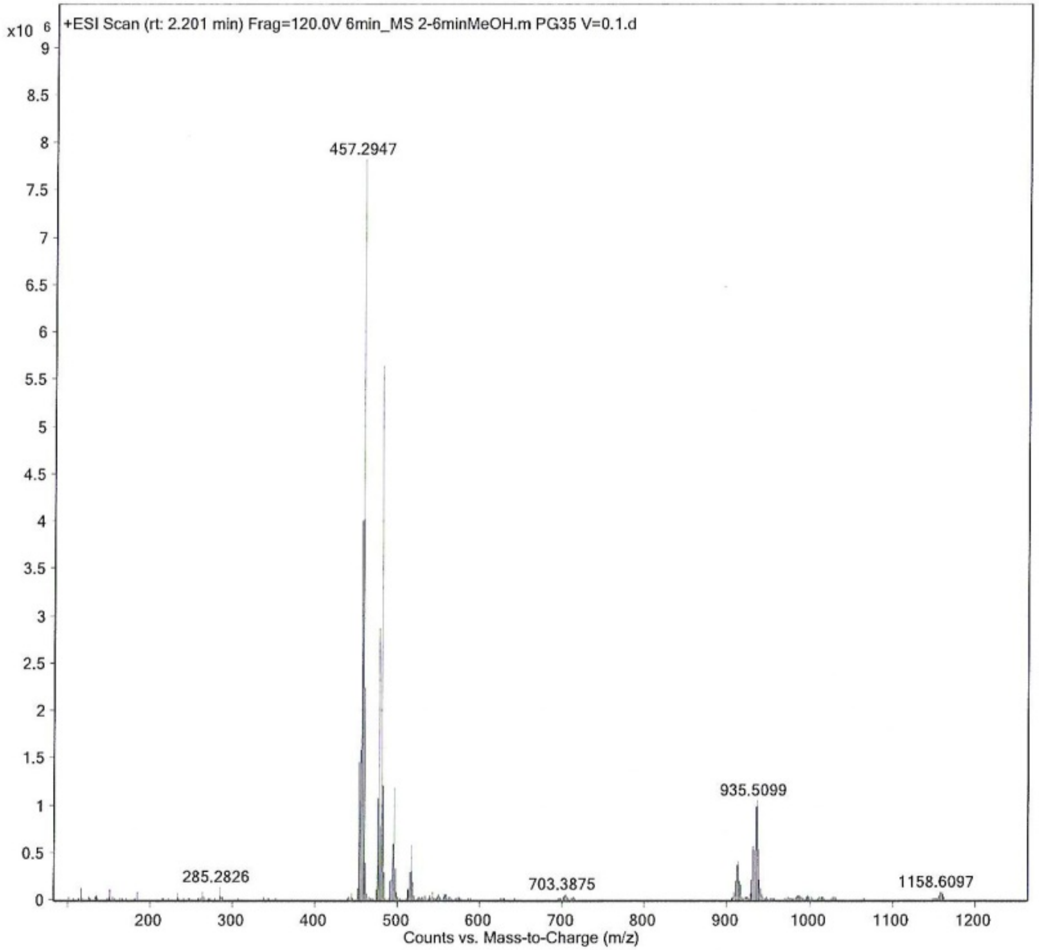

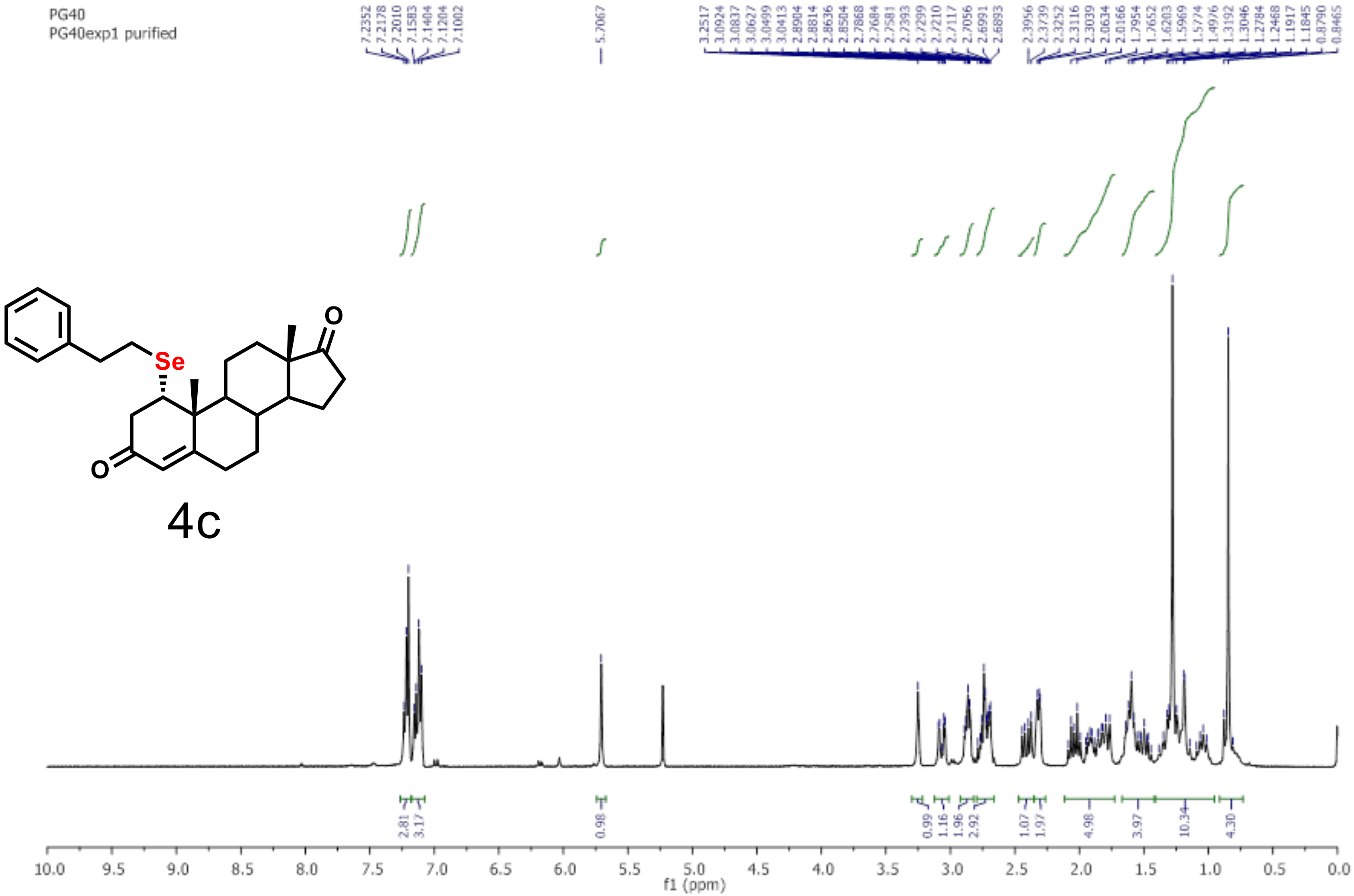

Se
O
O
4c
PG40
PG40exp1 purified
7.2352
7.2178
7.2010
7.1583
7.1404
7.1204
7.1002
5.7067
3.2517
3.0924
3.0837
3.0627
3.0499
3.0413
2.8904
2.8814
2.8636
2.8504
2.7868
2.7684
2.7581
2.7393
2.7299
2.7210
2.7117
2.7056
2.6991
2.6893
2.3956
2.3739
2.3252
2.3116
2.3039
2.0634
2.0166
1.7954
1.7652
1.6203
1.5969
1.5774
1.4976
1.3192
1.3046
1.2784
1.2468
1.1917
1.1845
0.8790
0.8465
2.81
3.17
0.98
0.99
1.16
1.96
2.92
1.07
1.97
4.98
3.97
10.34
4.30
10.0
9.5
9.0
8.5
8.0
7.5
7.0
6.5
6.0
5.5
5.0
4.5
4.0
3.5
3.0
2.5
2.0
1.5
1.0
0.5
0.0
f1 (ppm)

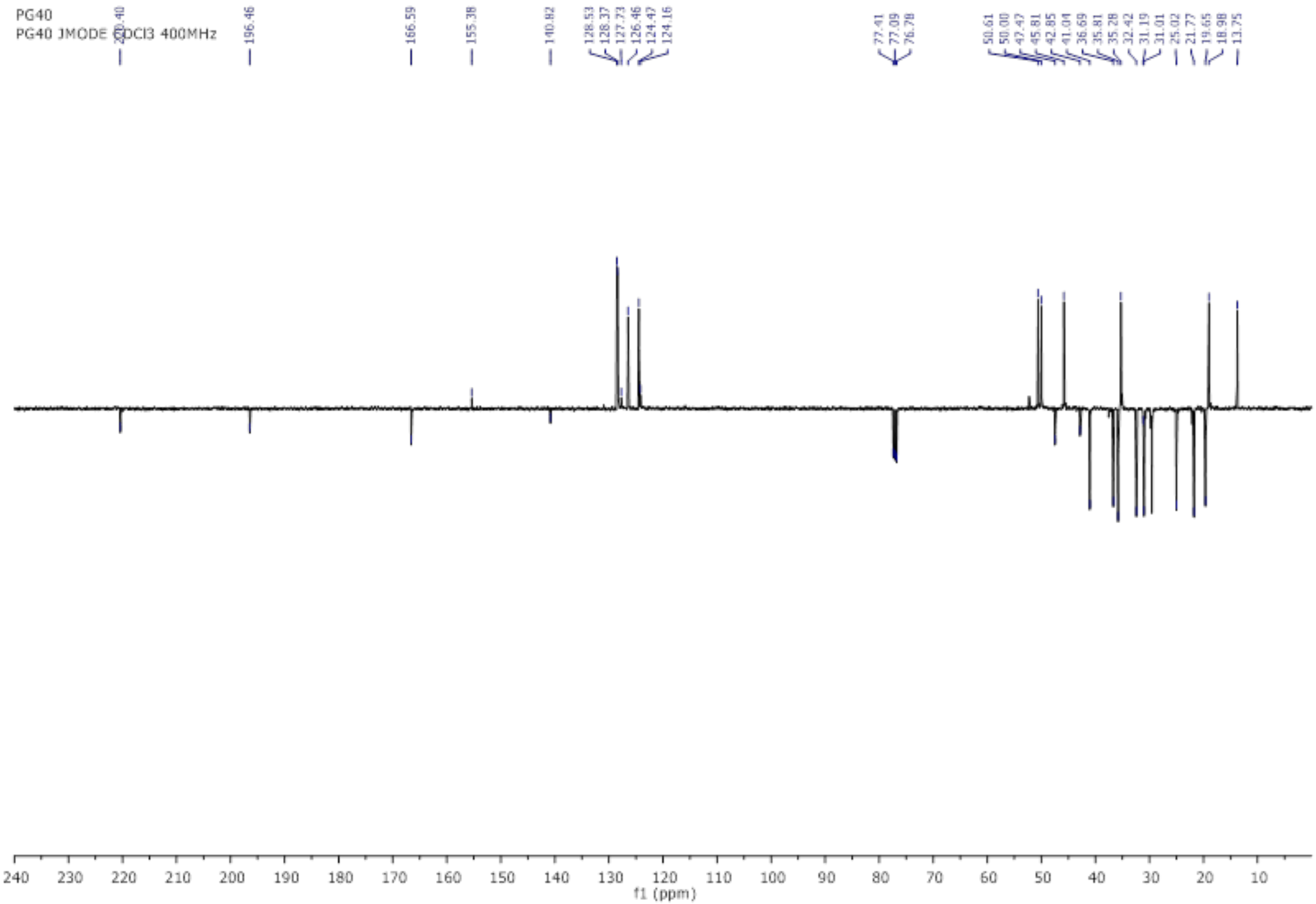
PG40
PG40 JMODE CDCl3 400MHz
220.40
196.46
166.59
155.38
140.82
128.53
128.37
127.73
126.46
124.47
124.16
77.41
77.09
76.78
50.61
50.00
47.47
45.81
42.85
41.04
36.69
35.81
35.28
32.42
31.19
31.01
25.02
21.77
19.65
18.98
13.75
240 230 220 210 200 190 180 170 160 150 140 130 120 110 100 90 80 70 60 50 40 30 20 10
f1 (ppm)

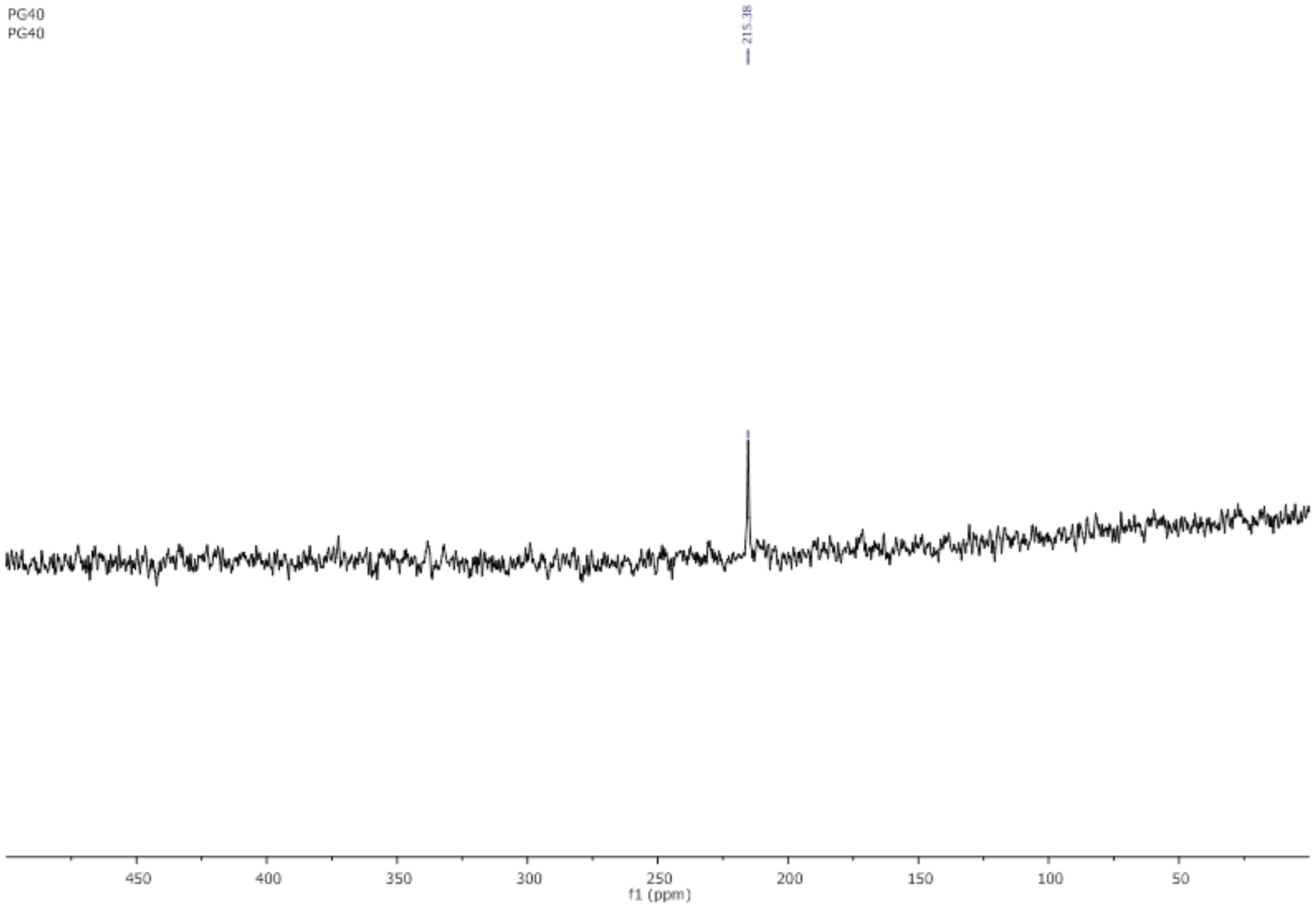
PG40
PG40
215.38
450
400
350
300
250
200
150
100
50
f1 (ppm)

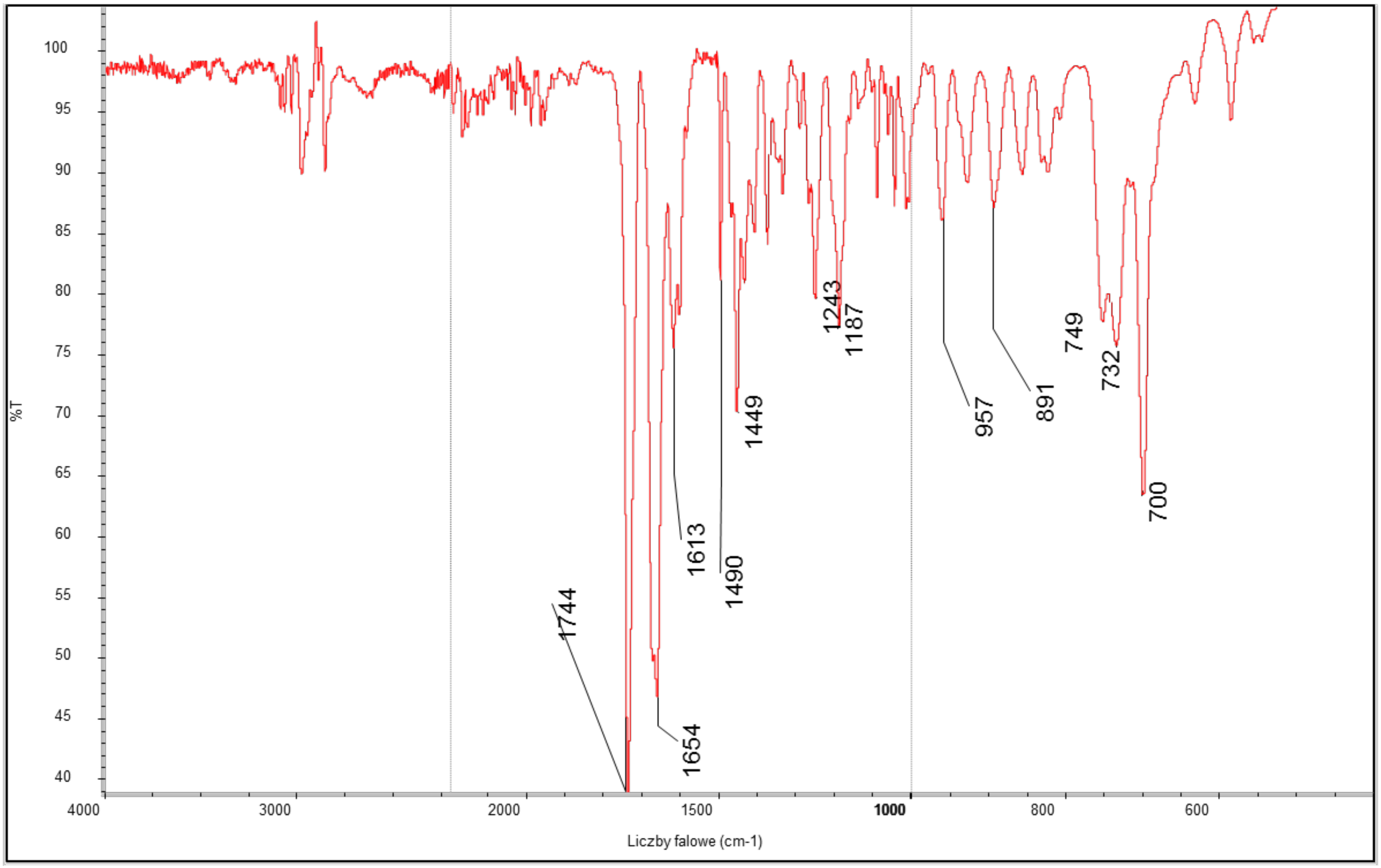
%T
100
95
90
85
80
75
70
65
60
55
50
45
40
4000
3000
2000
1500
1000
800
600
Liczby falowe (cm-1)
1744
1654
1613
1490
1449
1243
1187
957
891
749
732
700

**Sample Name**	PG40	**Position**	P1-B1	**Instrument Name**	Instrument 1
**User Name**		**Inj Vol**	0.1	**InjPosition**	
**Sample Type**	Sample	**IRM Calibration Status**	Success	**Data Filename**	6min_MS 2-6minMeOH.m PG40 V=0.1.d
**ACQ Method**	6min_MS 2-6minMeOH.m	**Comment**		**Acquired Time**	7/8/2019 6:19:36 PM

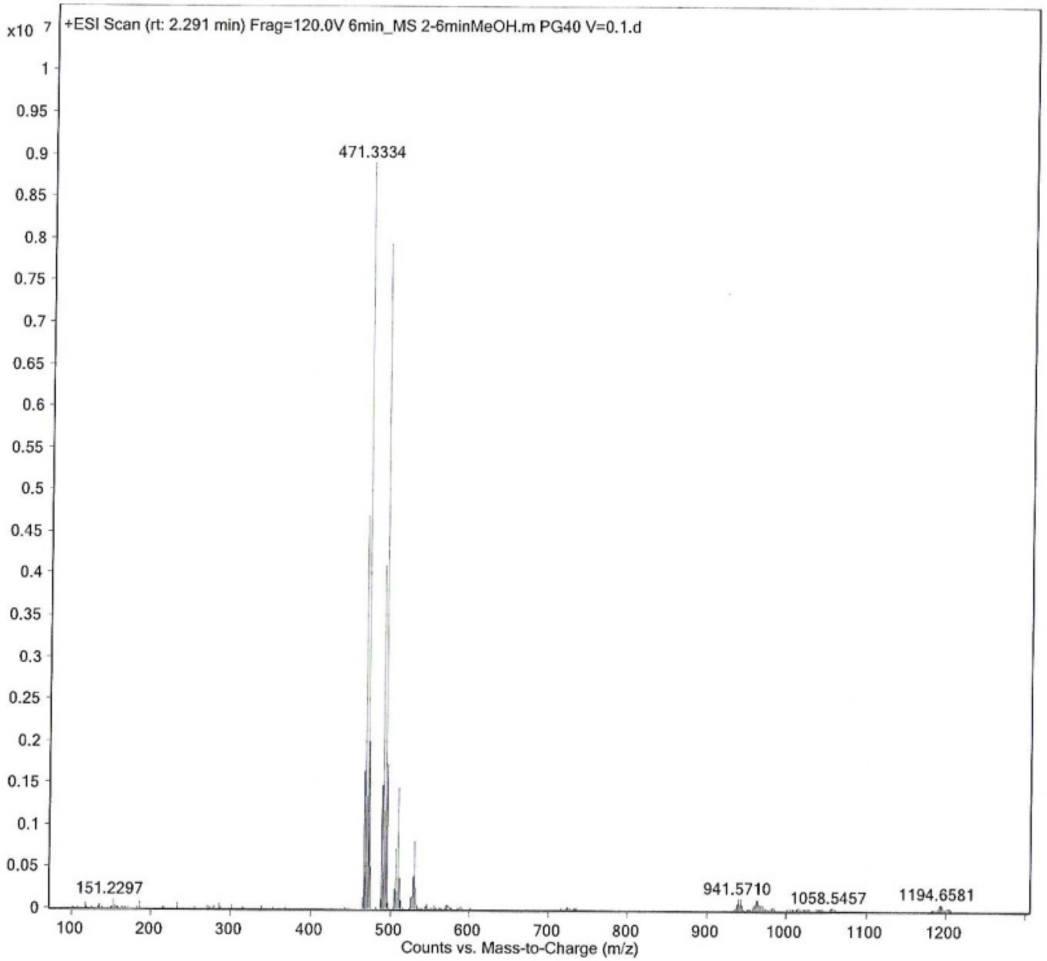

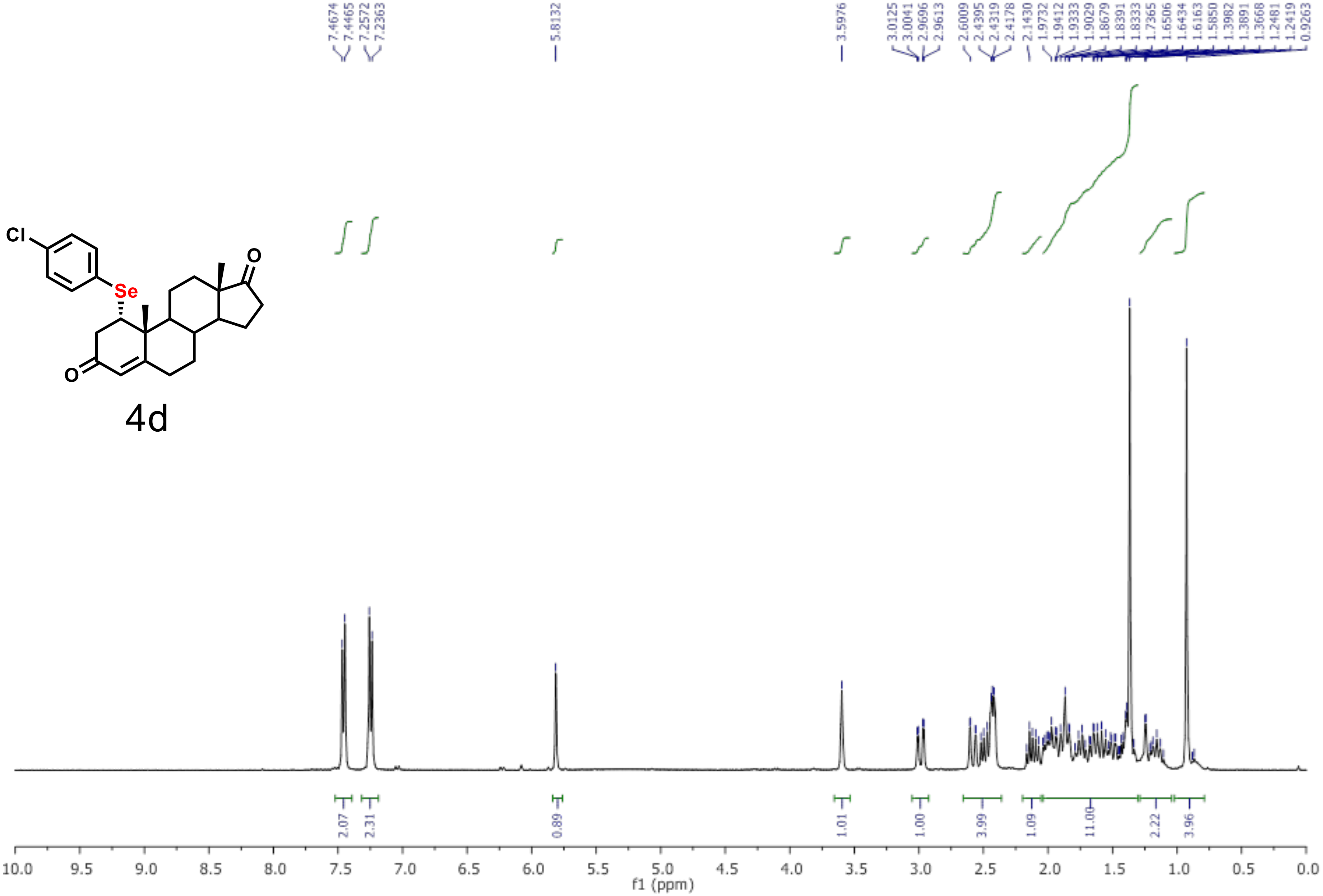

4d
7.4674
7.4465
7.2572
7.2363
5.8132
3.5976
3.0125
3.0041
2.9696
2.9613
2.6009
2.4395
2.4319
2.4178
2.1430
1.9732
1.9412
1.9333
1.9029
1.8679
1.8391
1.8333
1.7365
1.6506
1.6434
1.6163
1.5850
1.3982
1.3891
1.3668
1.2481
1.2419
0.9263
2.07
2.31
0.89
1.01
1.00
3.99
1.09
11.00
2.22
3.96
10.0 9.5 9.0 8.5 8.0 7.5 7.0 6.5 6.0 5.5 5.0 4.5 4.0 3.5 3.0 2.5 2.0 1.5 1.0 0.5 0.0
f1 (ppm)

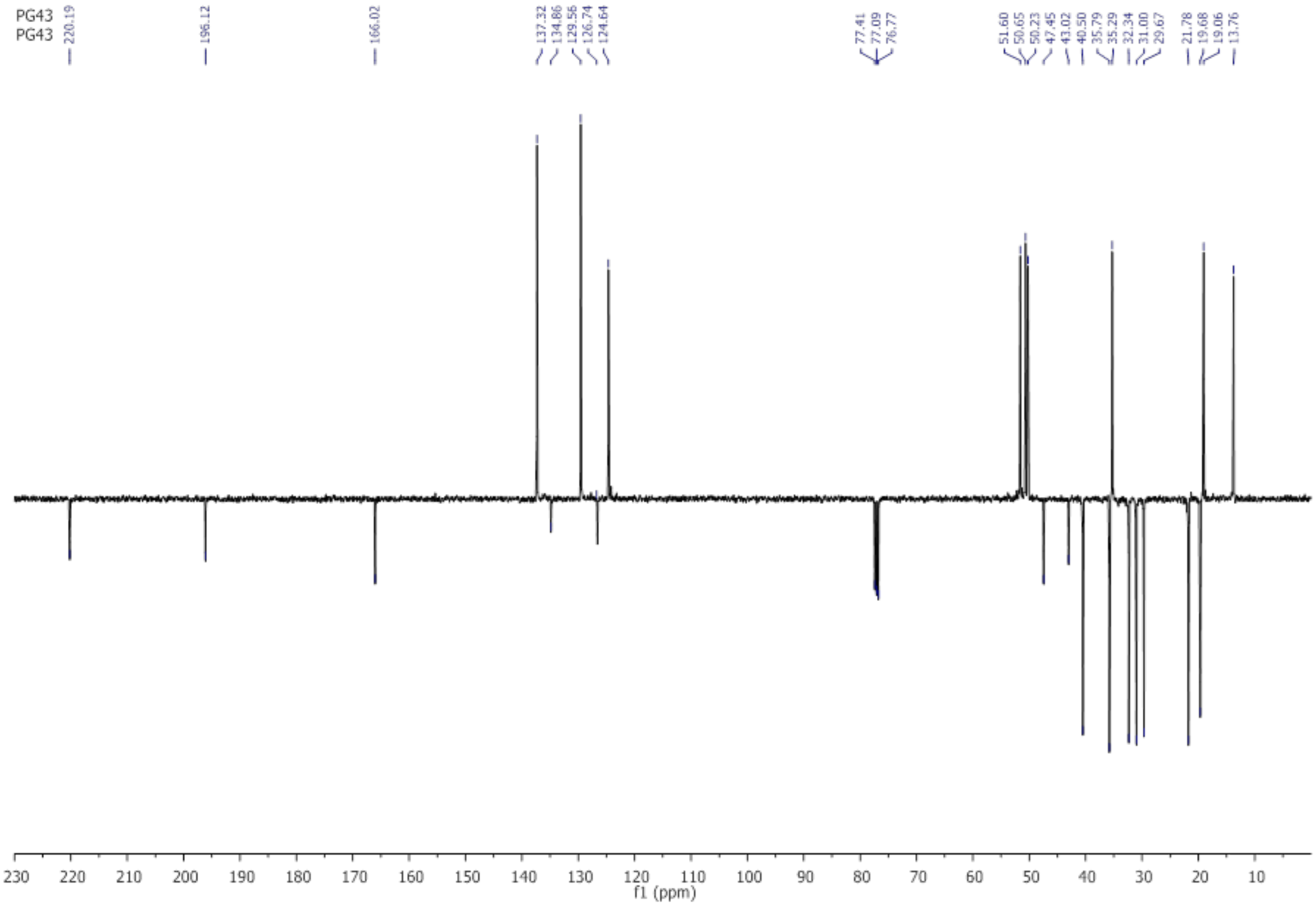
PG43
PG43
220.19
196.12
166.02
137.32
134.86
129.56
126.74
124.64
77.41
77.09
76.77
51.60
50.65
50.23
47.45
43.02
40.50
35.79
35.29
32.34
31.00
29.67
21.78
19.68
19.06
13.76
230 220 210 200 190 180 170 160 150 140 130 120 110 100 90 80 70 60 50 40 30 20 10
f1 (ppm)

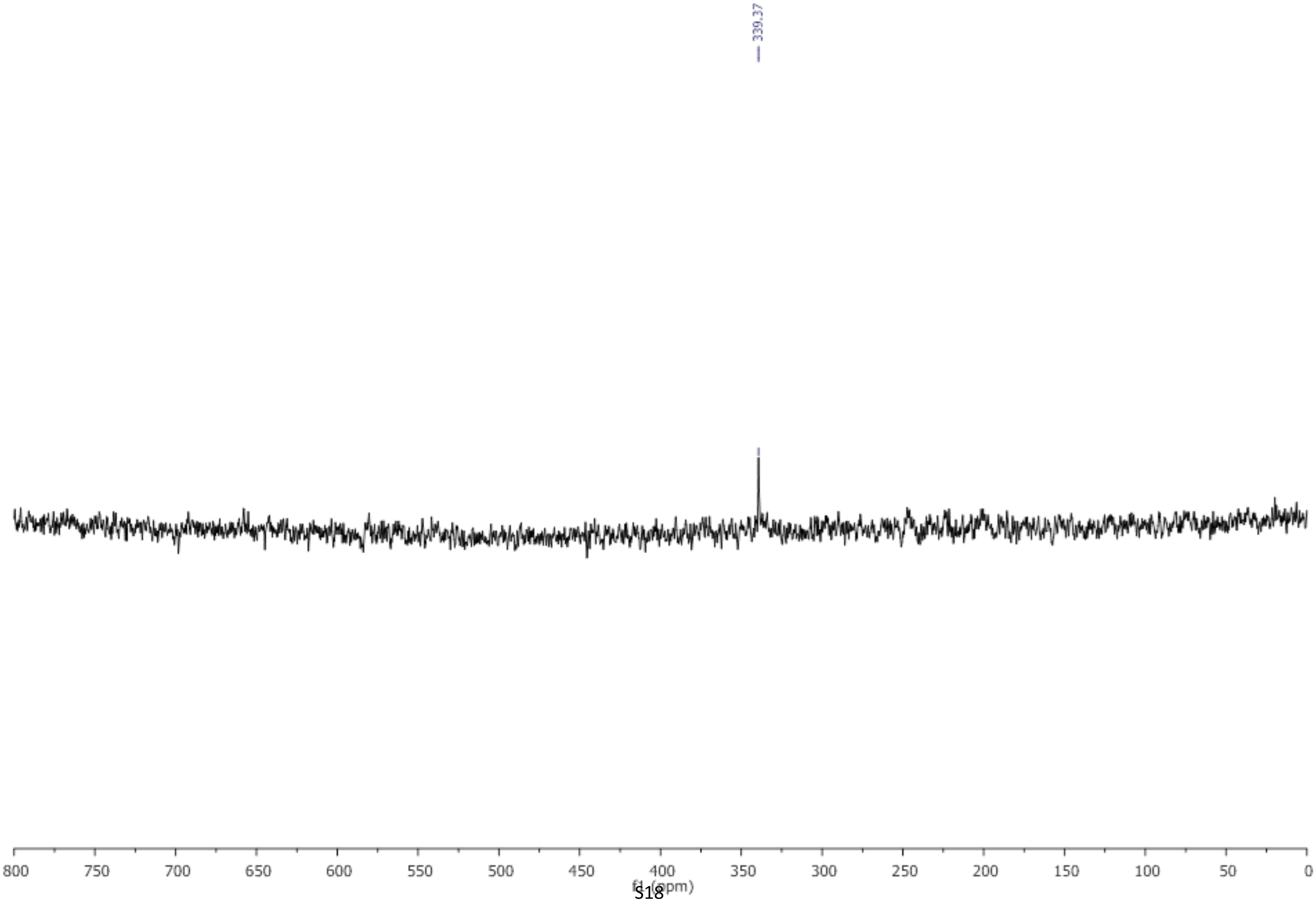
339.37
800 750 700 650 600 550 500 450 400 350 300 250 200 150 100 50 0
f1 (ppm)

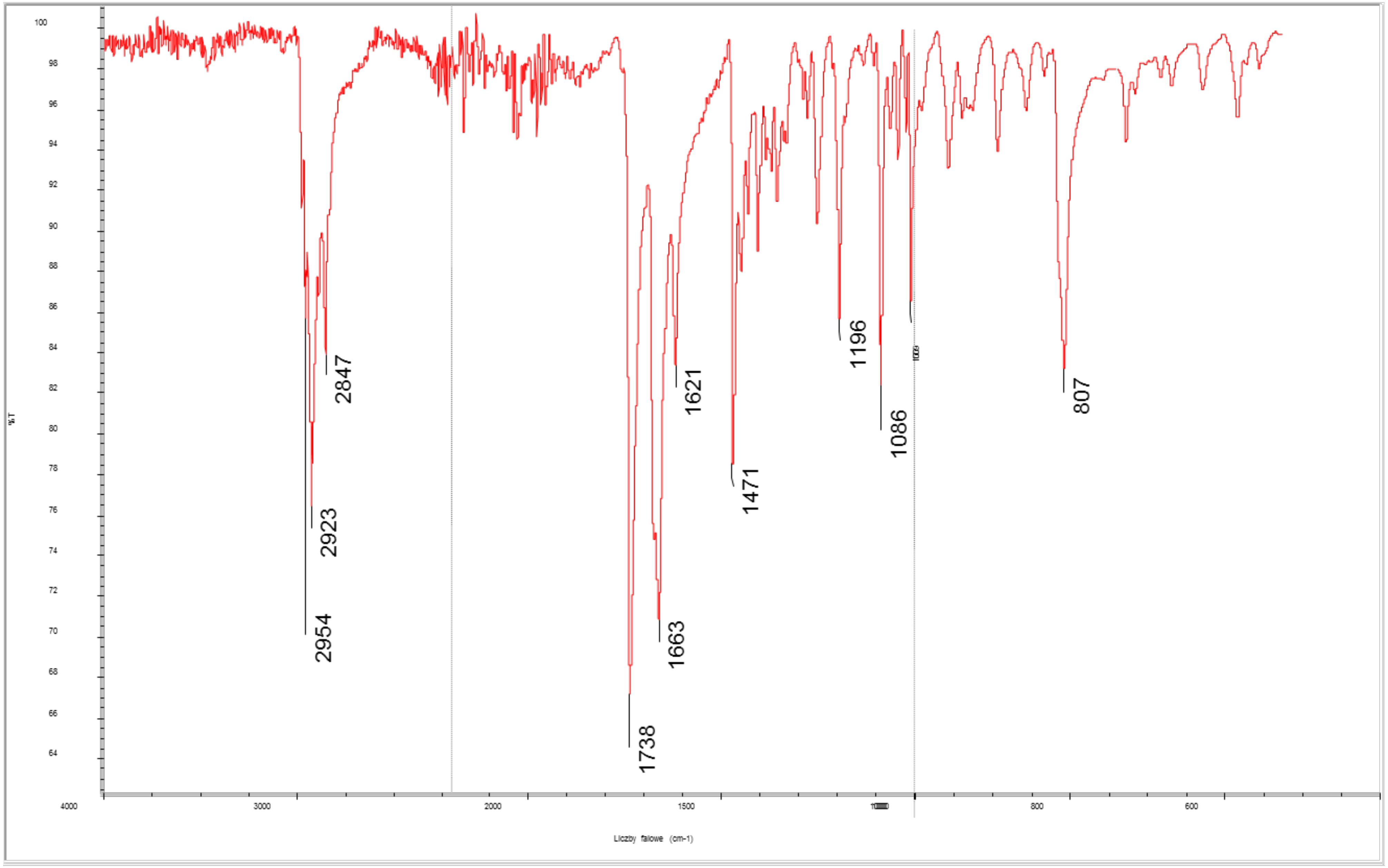

%T
Liczby falowe (cm-1)
2954
2923
2847
1738
1663
1621
1471
1196
1086
807

**Sample Name**	PG43	**Position**	P1-B1	**Instrument Name**	Instrument 1
**User Name**		**Inj Vol**	0.1	**InjPosition**	
**Sample Type**	Sample	**IRM Calibration Status**	Success	**Data Filename**	6min_MS 2-6minMeOH.m PG43.d
**ACQ Method**	6min_MS 2-6minMeOH.m	**Comment**		**Acquired Time**	7/17/2019 1:11:30 PM

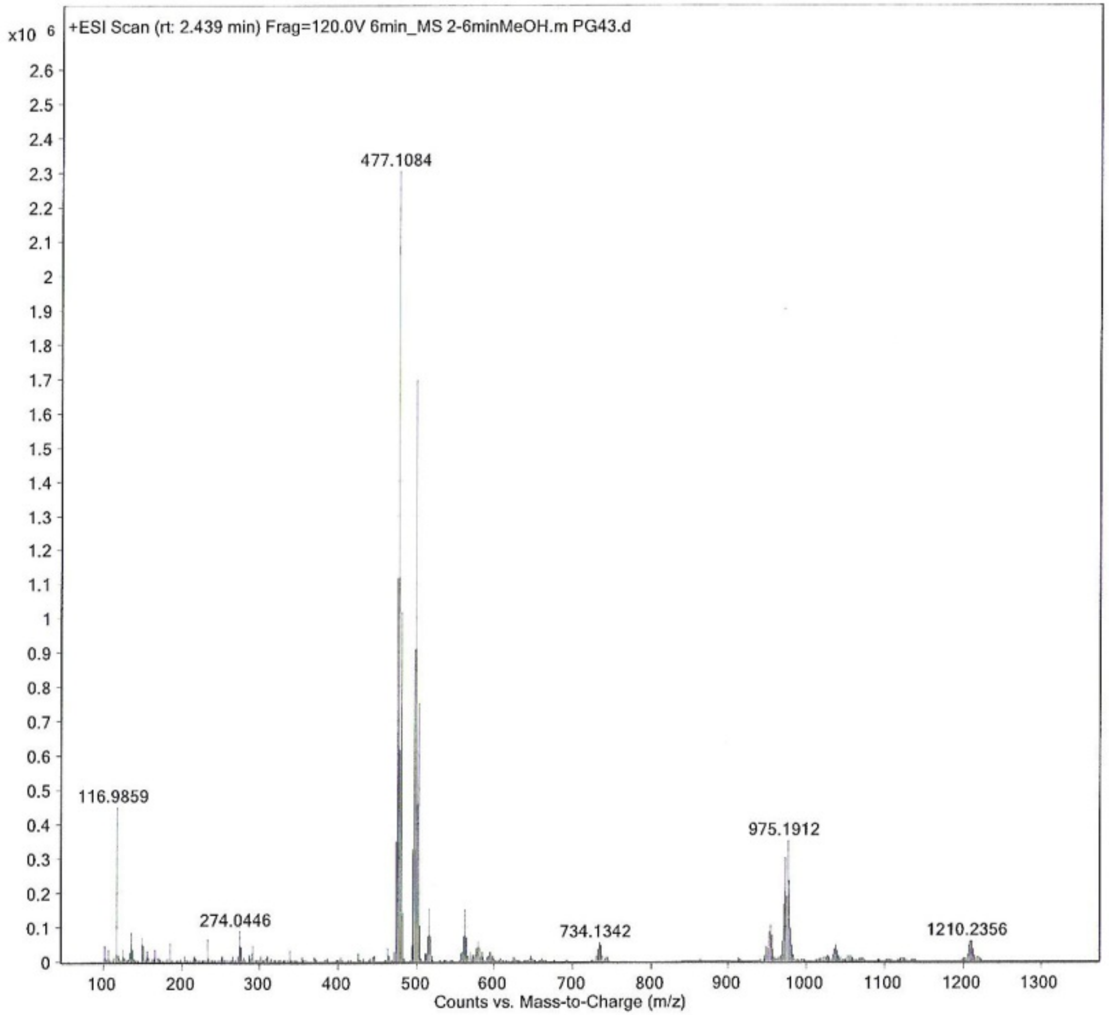

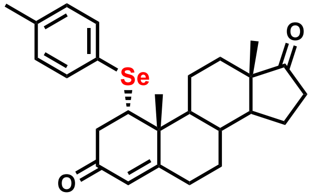

4e

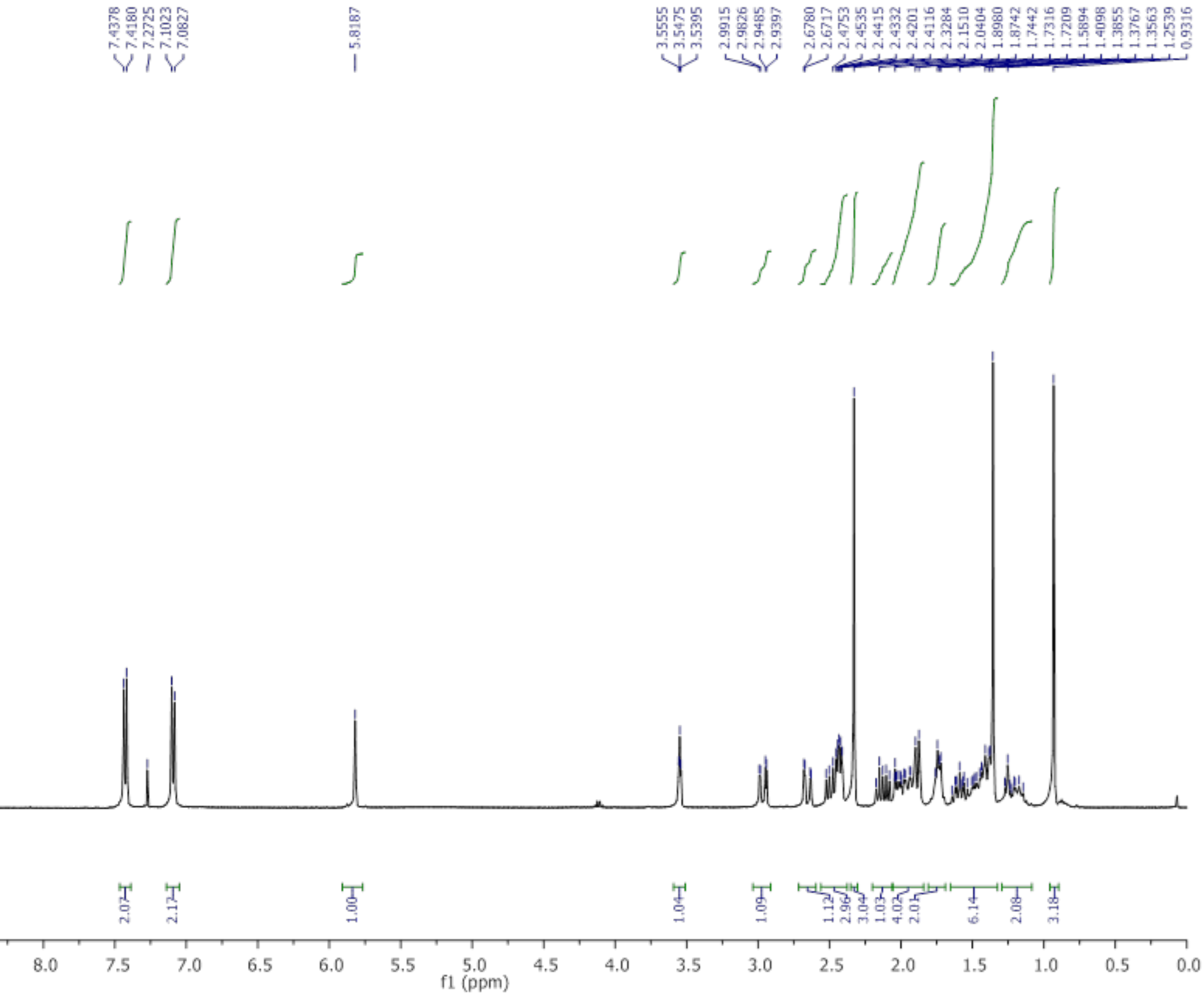

7.4378
7.4180
7.2725
7.1023
7.0827
5.8187
3.5555
3.5475
3.5395
2.9915
2.9826
2.9485
2.9397
2.6780
2.6717
2.4753
2.4535
2.4415
2.4332
2.4201
2.4116
2.3284
2.1510
2.0404
1.8980
1.8742
1.7442
1.7316
1.7209
1.5894
1.4098
1.3855
1.3767
1.3563
1.2539
0.9316
2.07
2.17
1.00
1.04
1.09
1.12
2.96
3.04
1.03
4.02
2.01
6.14
2.08
3.18
10.0
9.5
9.0
8.5
8.0
7.5
7.0
6.5
6.0
5.5
5.0
4.5
4.0
3.5
3.0
2.5
2.0
1.5
1.0
0.5
0.0
f1 (ppm)

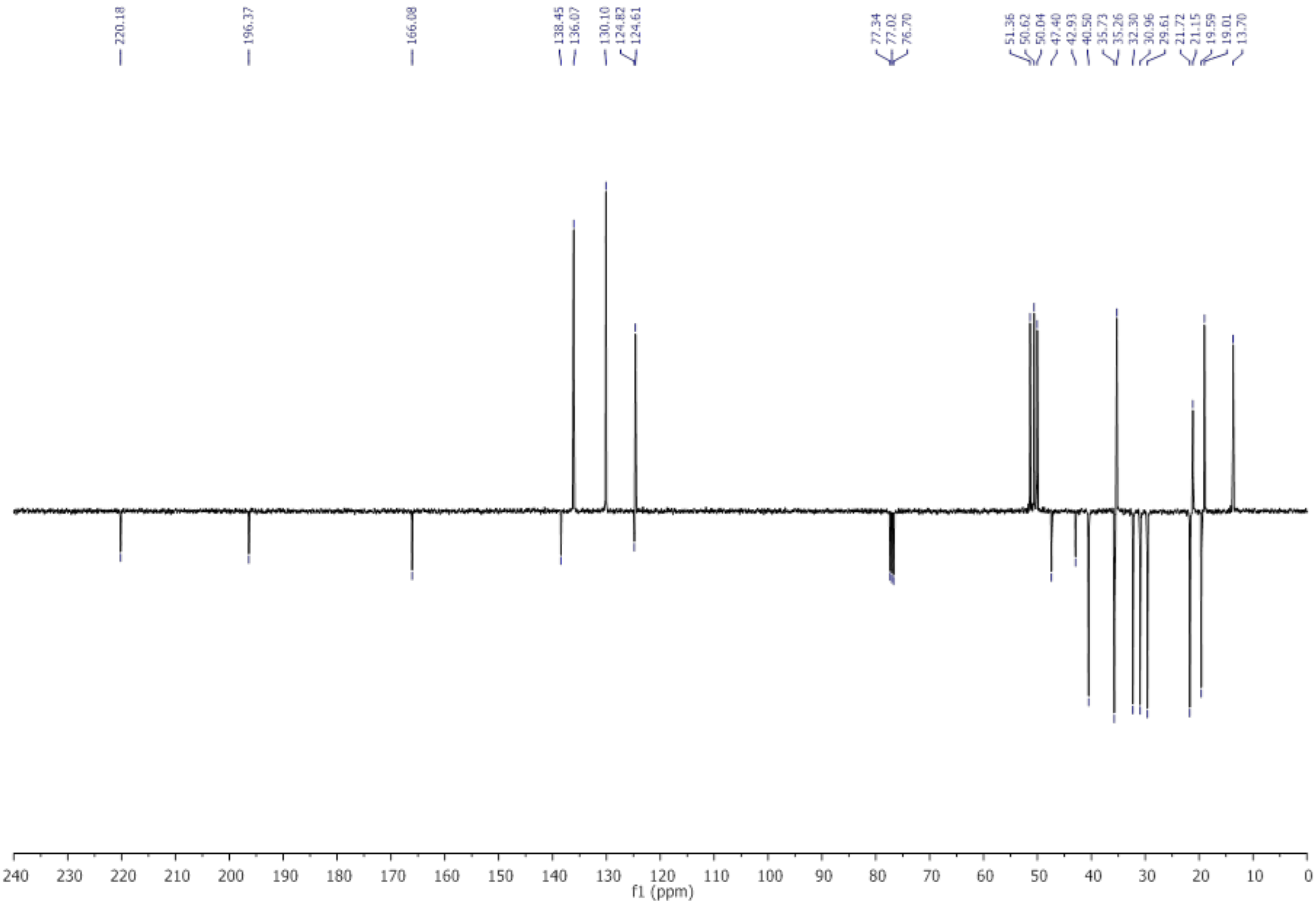
220.18
196.37
166.08
138.45
136.07
130.10
124.82
124.61
77.34
77.02
76.70
51.36
50.62
50.04
47.40
42.93
40.50
35.73
35.26
32.30
30.96
29.61
21.72
21.15
19.59
19.01
13.70
240 230 220 210 200 190 180 170 160 150 140 130 120 110 100 90 80 70 60 50 40 30 20 10 0
f1 (ppm)

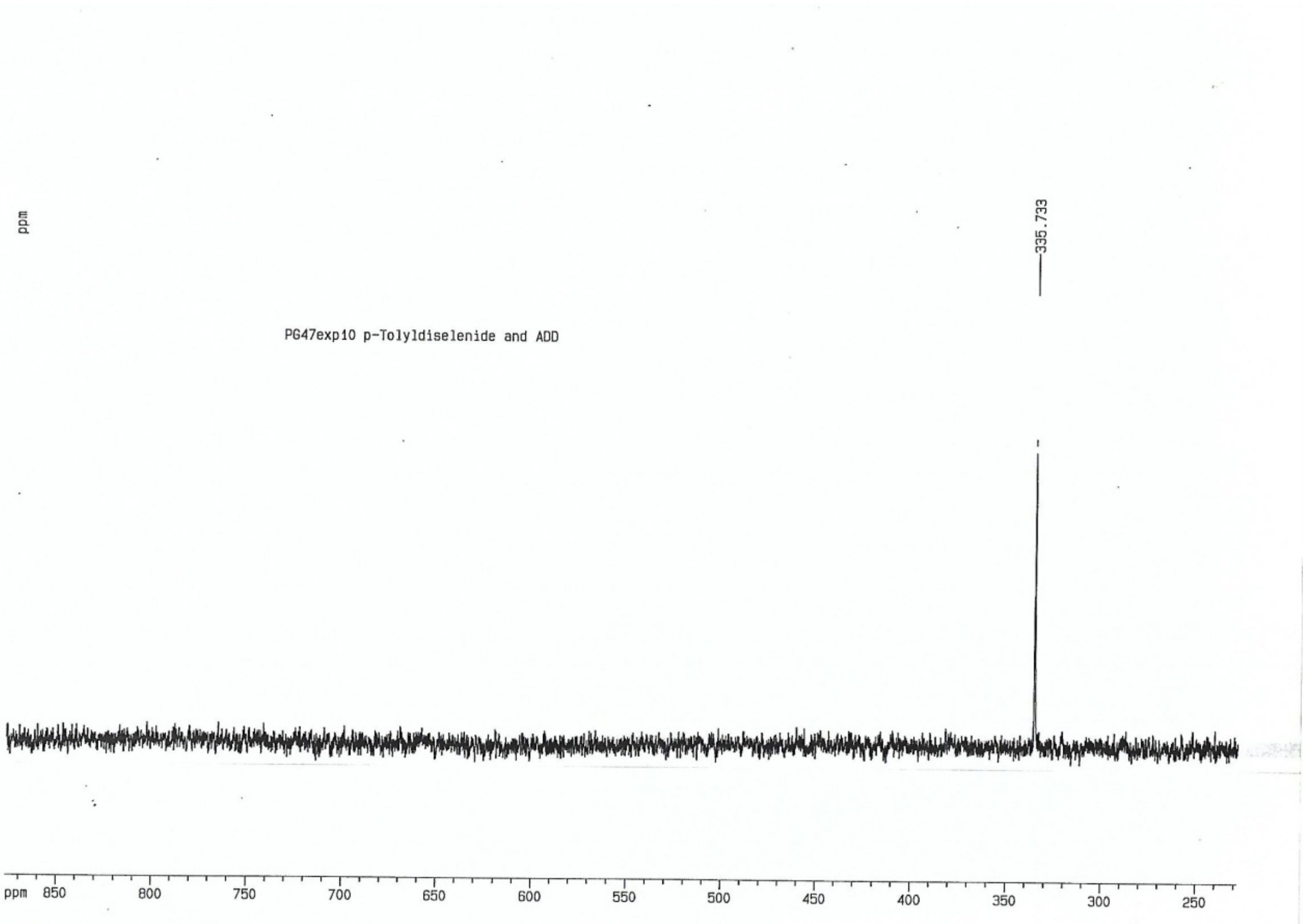
ppm
335.733
PG47exp10 p-Tolyldiselenide and ADD
ppm 850 800 750 700 650 600 550 500 450 400 350 300 250

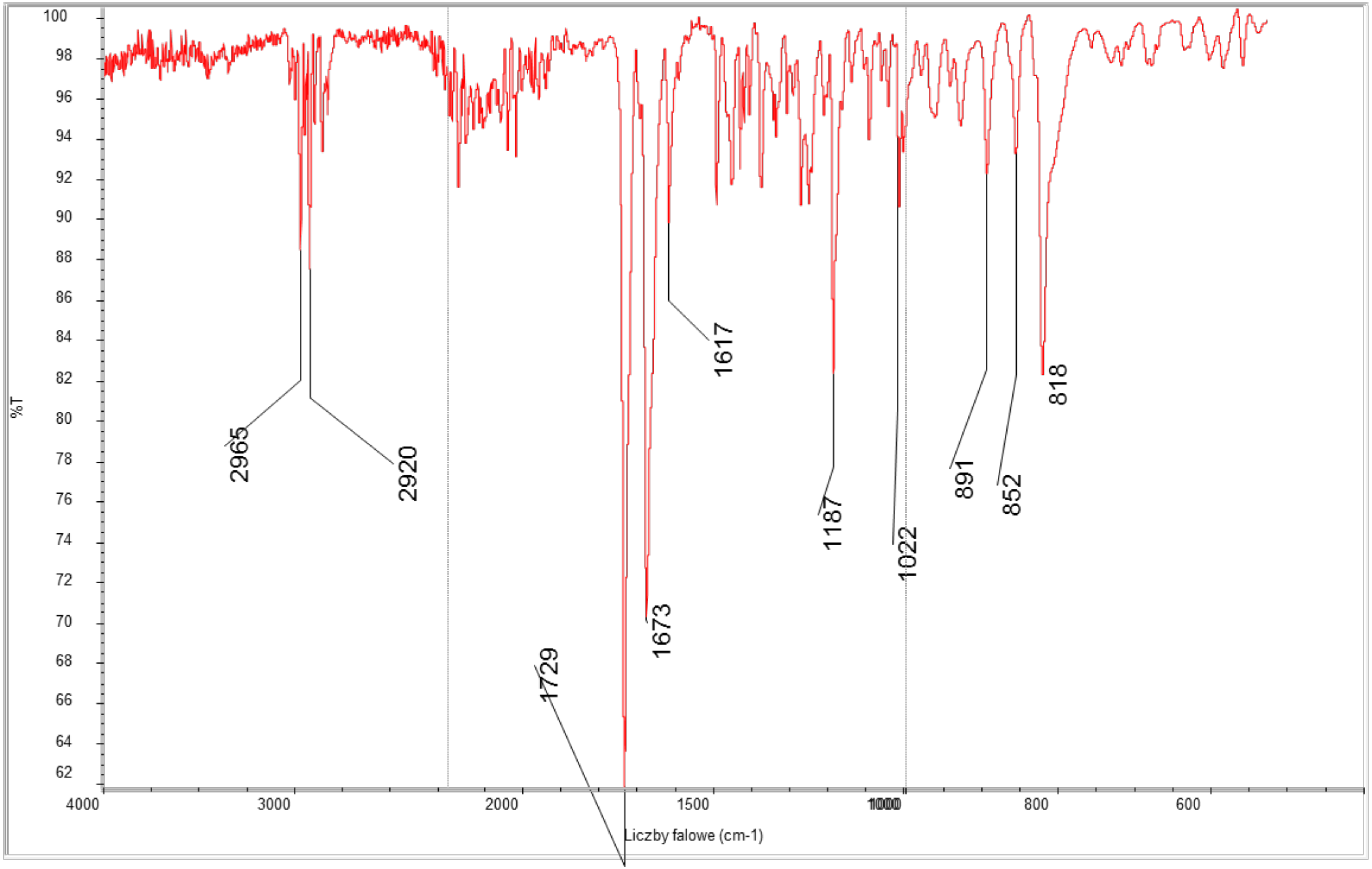

100
98
96
94
92
90
88
86
84
82
80
78
76
74
72
70
68
66
64
62
%T
4000
3000
2000
1500
1000
800
600
Liczby falowe (cm-1)
2965
2920
1729
1673
1617
1187
1022
891
852
818

**Sample Name**	PG47	**Position**	P1-B1	**Instrument Name**	Instrument 1
**User Name**		**Inj Vol**	0.1	**InjPosition**	
**Sample Type**	Sample	**IRM Calibration Status**	Success	**Data Filename**	6min_MS 2-6minMeOH.m PG47.d
**ACQ Method**	6min_MS 2-6minMeOH.m	**Comment**		**Acquired Time**	7/17/2019 1:49:48 PM

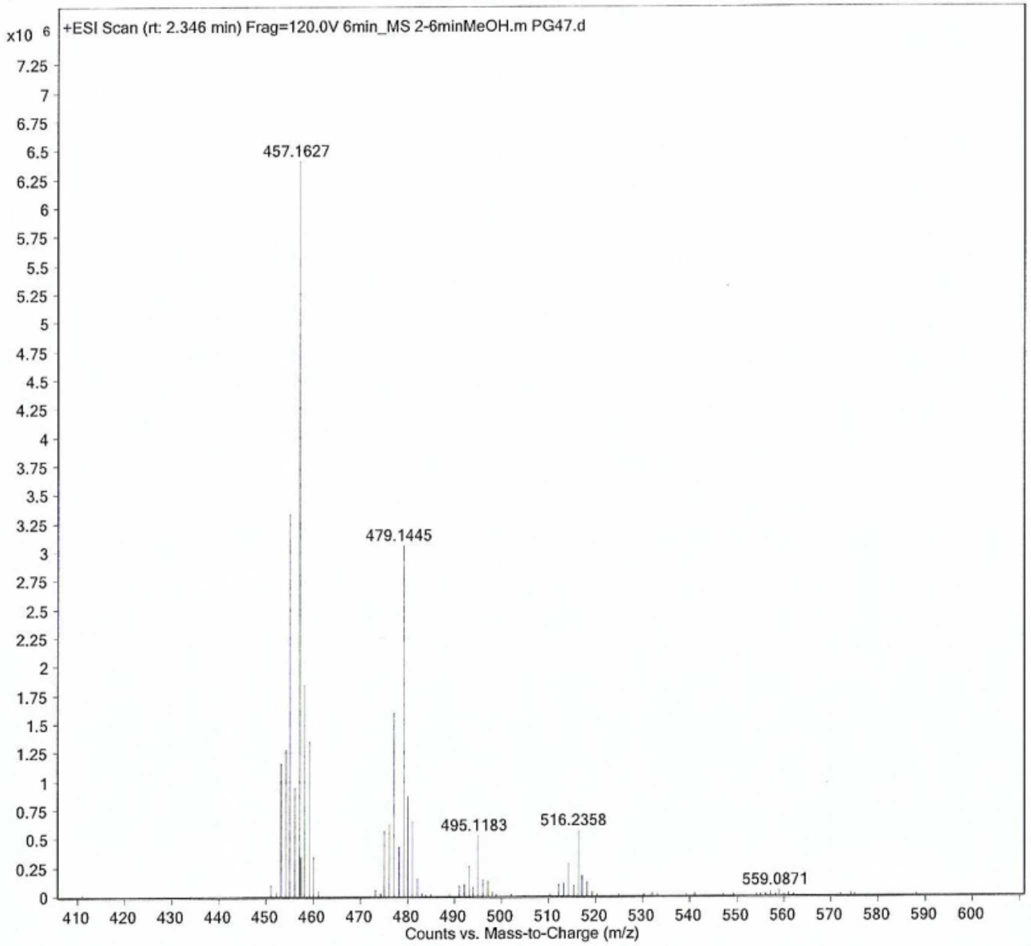

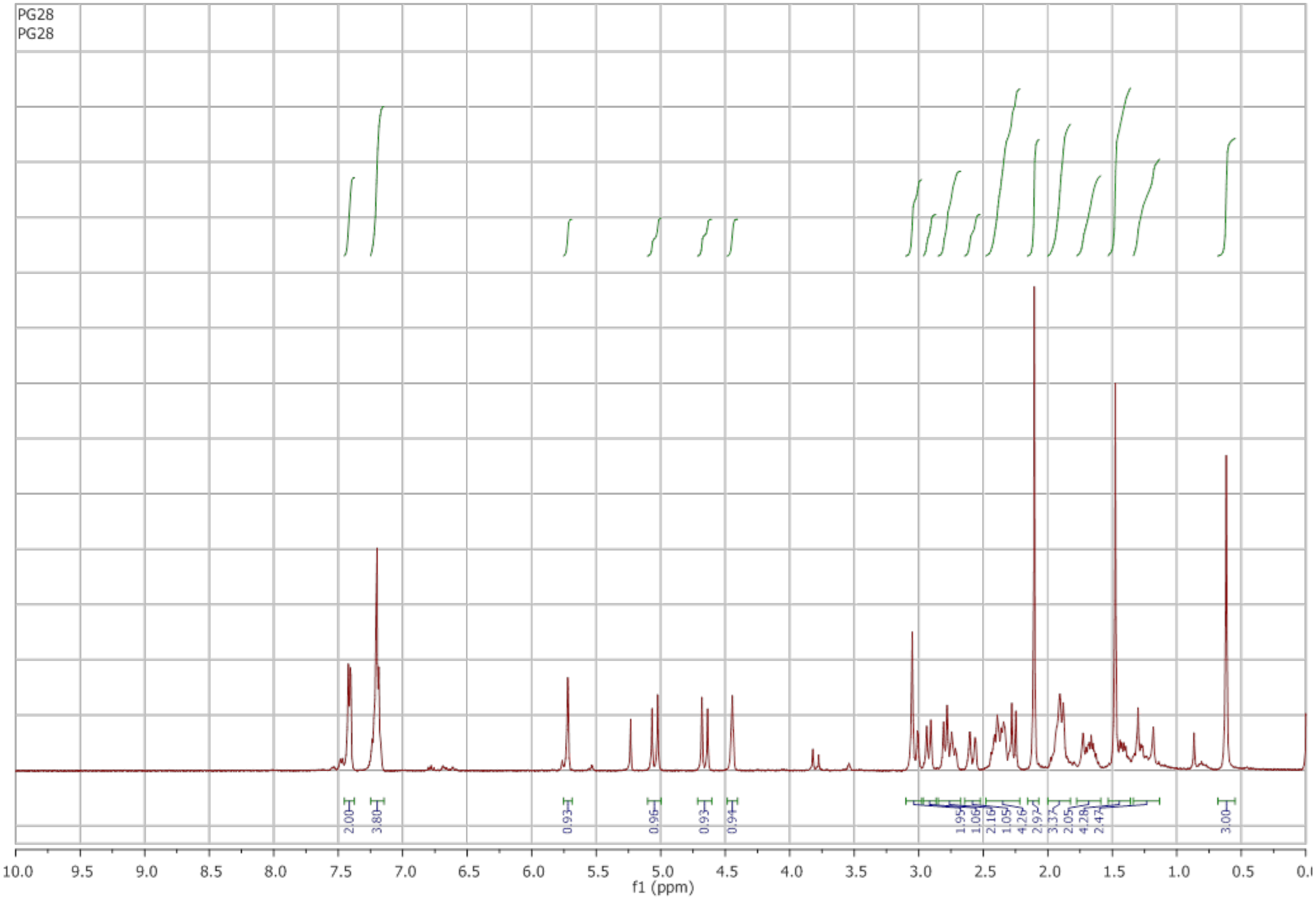

PG28
PG28
2.00
3.80
0.93
0.96
0.93
0.94
1.95
1.06
2.16
1.05
4.26
2.92
3.37
2.05
4.28
2.47
3.00
10.0
9.5
9.0
8.5
8.0
7.5
7.0
6.5
6.0
5.5
5.0
4.5
4.0
3.5
3.0
2.5
2.0
1.5
1.0
0.5
f1 (ppm)

PG 28 DEPTy

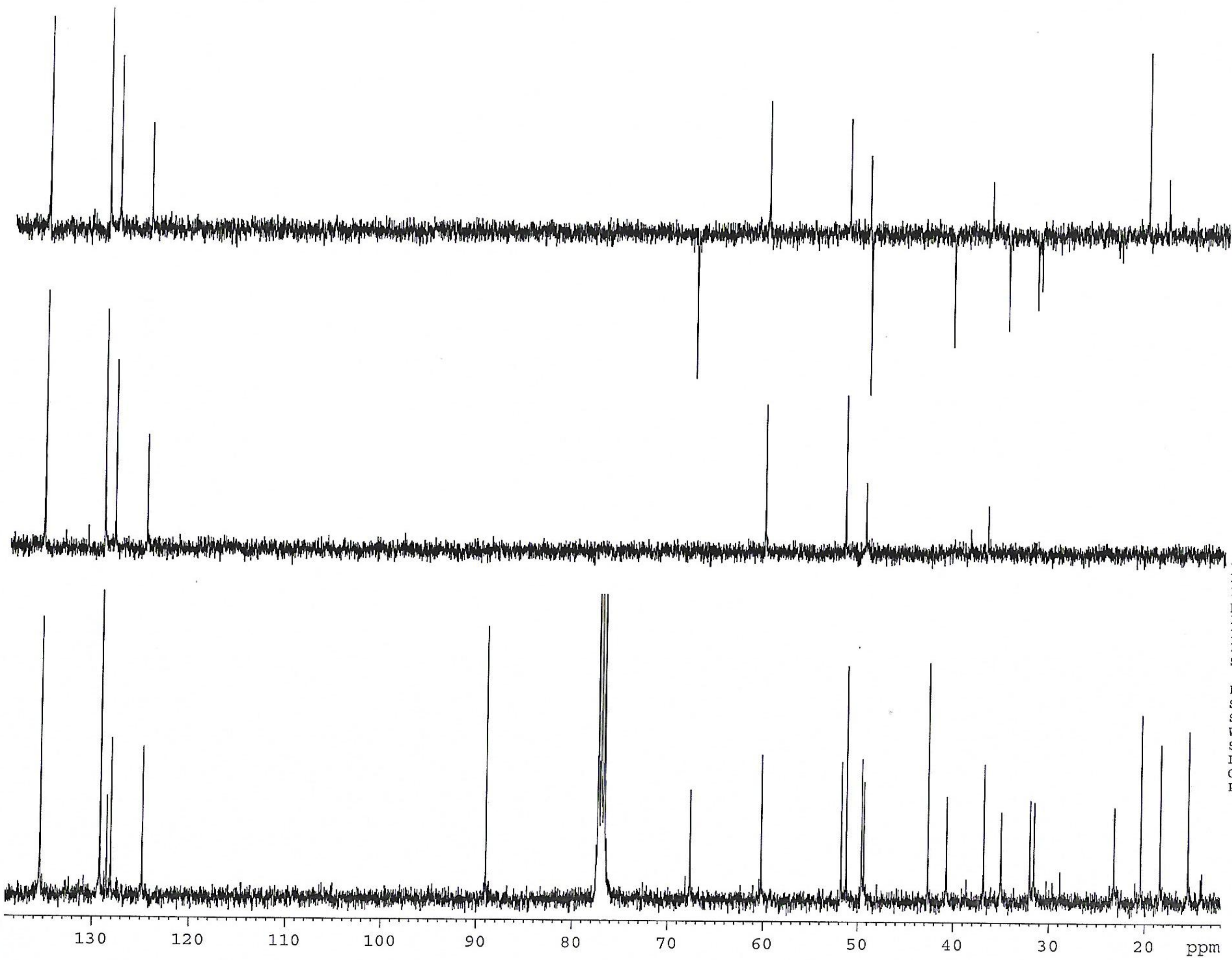

Current Data Parameters
NAME PG 28
EXPNO 4
PROCNO 1

F2 - Acquisition Parameters
Date_ 20190926
Time 11.30
INSTRUM spect
PROBHD 5 mm PABBO BB-
PULPROG dept135
TD 65536
SOLVENT CDCl3
NS 320
DS 4
SWH 36231.883 Hz
FIDRES 0.552855 Hz
AQ 0.9044468 sec
RG 2050
DW 13.800 usec
DE 6.00 usec
TE 999.9 K
CNST2 145.0000000
D1 2.00000000 sec
d2 0.00344828 sec
d12 0.00002000 sec
DELTA 0.00003501 sec
TD0 1

======== CHANNEL f1 ========
NUC1 13C
P1 27.50 usec
p2 55.00 usec
PL1 -1.00 dB
SFO1 100.6253443 MHz

======== CHANNEL f2 ========
CPDPRG2 waltz16
NUC2 1H
P3 25.00 usec
p4 50.00 usec
PCPD2 100.00 usec
PL2 -3.00 dB
PL12 13.65 dB
SFO2 400.1516006 MHz

F2 - Processing parameters
SI 32768
SF 100.6178017 MHz
WDW EM
SSB 0
LB 1.00 Hz
GB 0
PC 0.20

PG 28 77Se

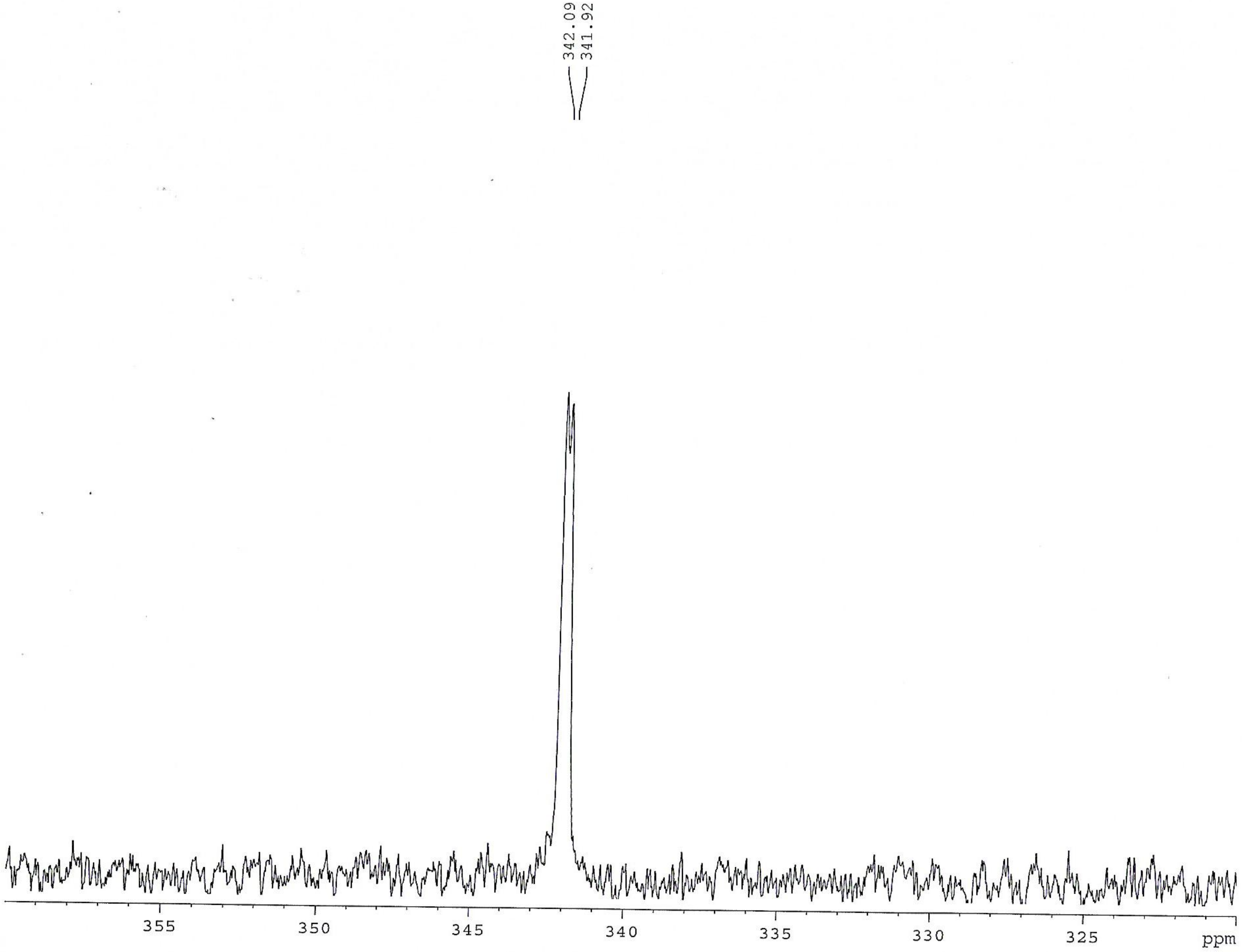

Current Data Parameters
NAME PG 28
EXPNO 8
PROCNO 1

F2 - Acquisition Parameters
Date_ 20190926
Time 9.15
INSTRUM spect
PROBHD 5 mm PABBO BB-
PULPROG zg
TD 65536
SOLVENT CDCl3
NS 14000
DS 4
SWH 62500.000 Hz
FIDRES 0.953674 Hz
AQ 0.5243380 sec
RG 2050
DW 8.000 usec
DE 6.00 usec
TE 999.9 K
D1 3.00000000 sec
TD0 1

======== CHANNEL f1 ========
NUC1 77Se
P1 10.00 usec
PL1 1.00 dB
SFO1 76.3528163 MHz

F2 - Processing parameters
SI 32768
SF 76.3146808 MHz
WDW EM
SSB 0
LB 3.00 Hz
GB 0
PC 0.20

**Sample Name**	Unavailable	**Position**	Unavailable	**Instrument Name**	Unavailable
**User Name**	Unavailable	**Inj Vol**	Unavailable	**InjPosition**	Unavailable
**Sample Type**	Unavailable	**IRM Calibration Status**	Success	**Data Filename**	6min_MS 2-6minMeOH.m PG28 V=0.1-2.d
**ACQ Method**		**Comment**	Sample information is unavailable	**Acquired Time**	Unavailable

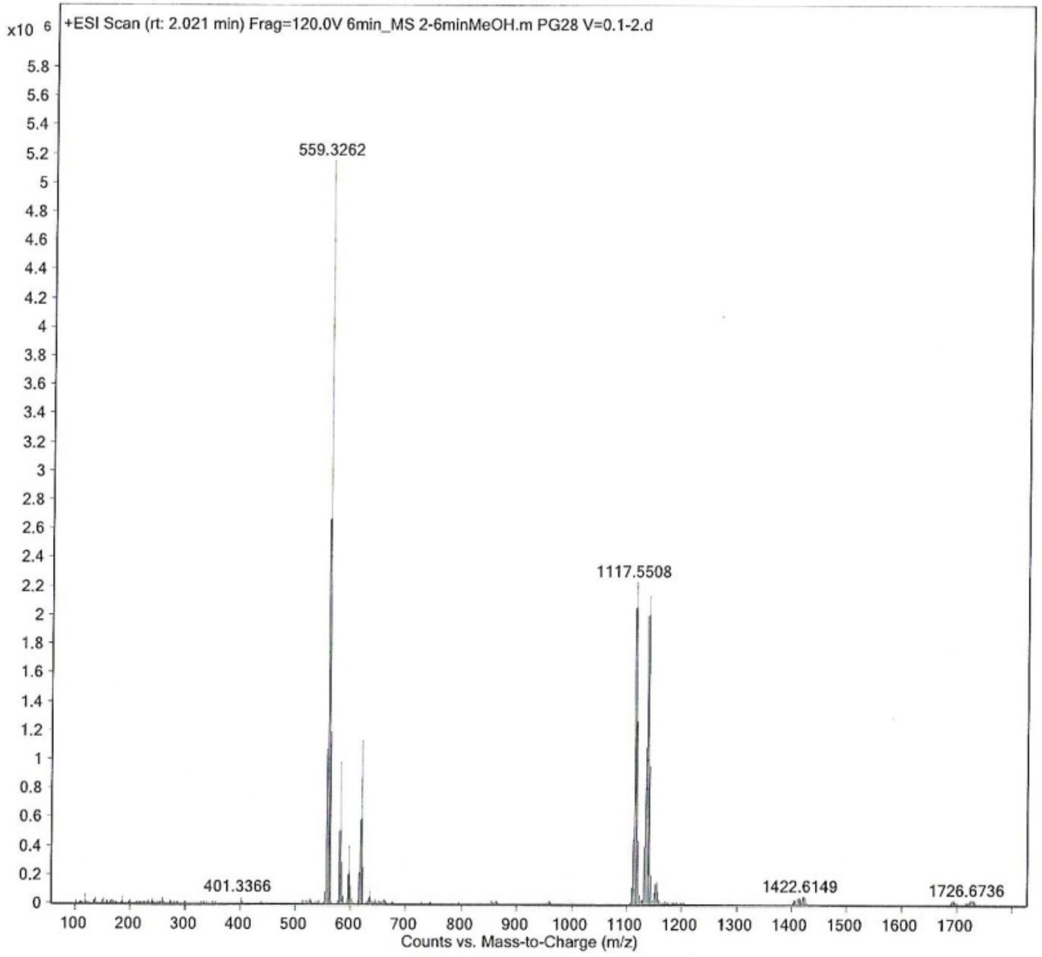

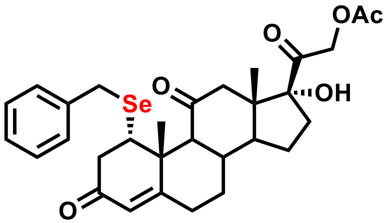

OAc
O
O
Se
OH
O

5b

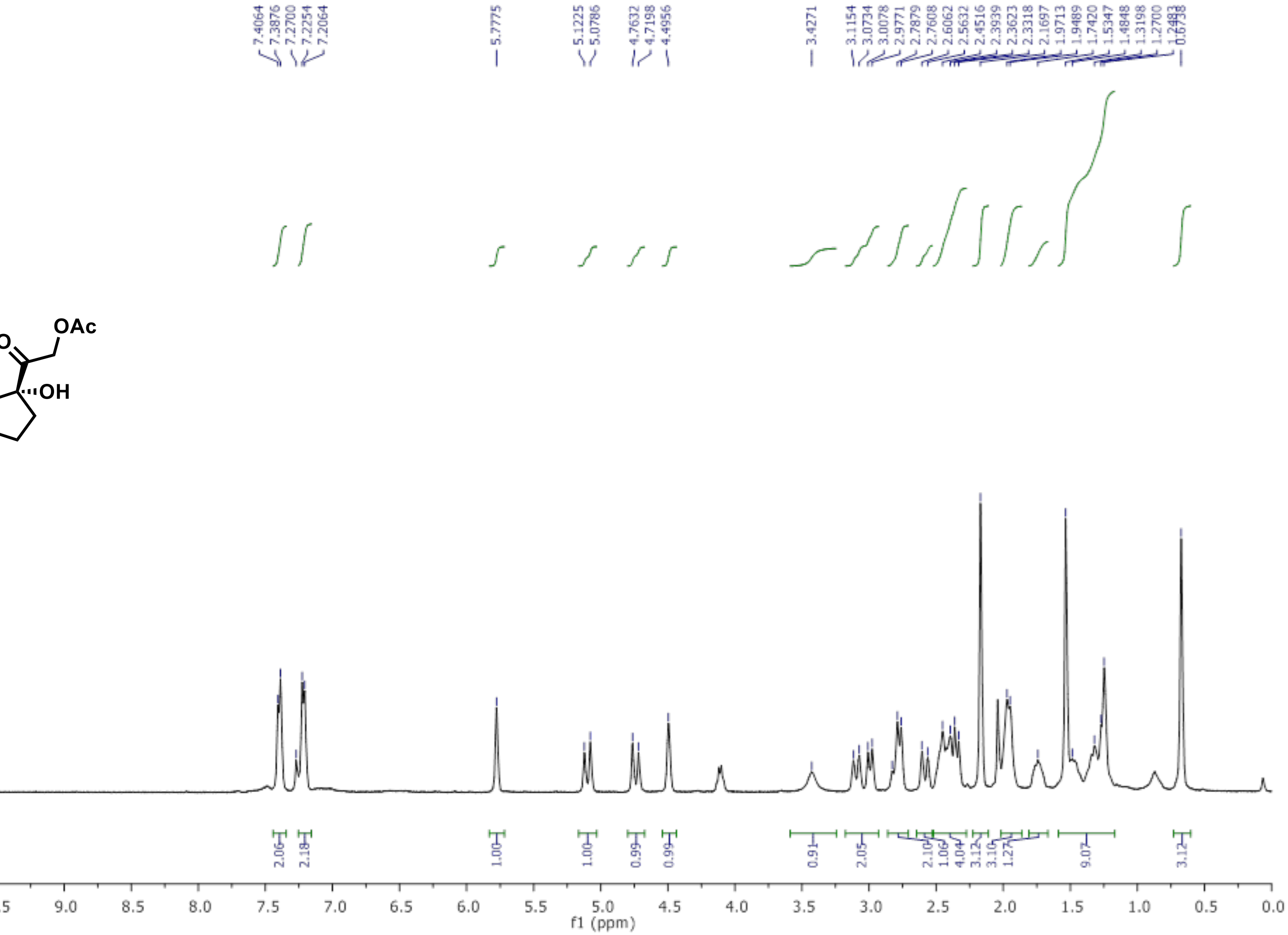

7.4064
7.3876
7.2700
7.2254
7.2064
5.7775
5.1225
5.0786
4.7632
4.7198
4.4956
3.4271
3.1154
3.0734
3.0078
2.9771
2.7879
2.7608
2.6062
2.5632
2.4516
2.3939
2.3623
2.3318
2.1697
1.9713
1.9489
1.7420
1.5347
1.4848
1.3198
1.2700
2.06
2.18
1.00
1.00
0.99
0.99
0.91
2.05
2.10
1.06
4.04
3.12
3.10
1.27
9.07
3.12
10.0
9.5
9.0
8.5
8.0
7.5
7.0
6.5
6.0
5.5
5.0
4.5
4.0
3.5
3.0
2.5
2.0
1.5
1.0
0.5
0.0
f1 (ppm)

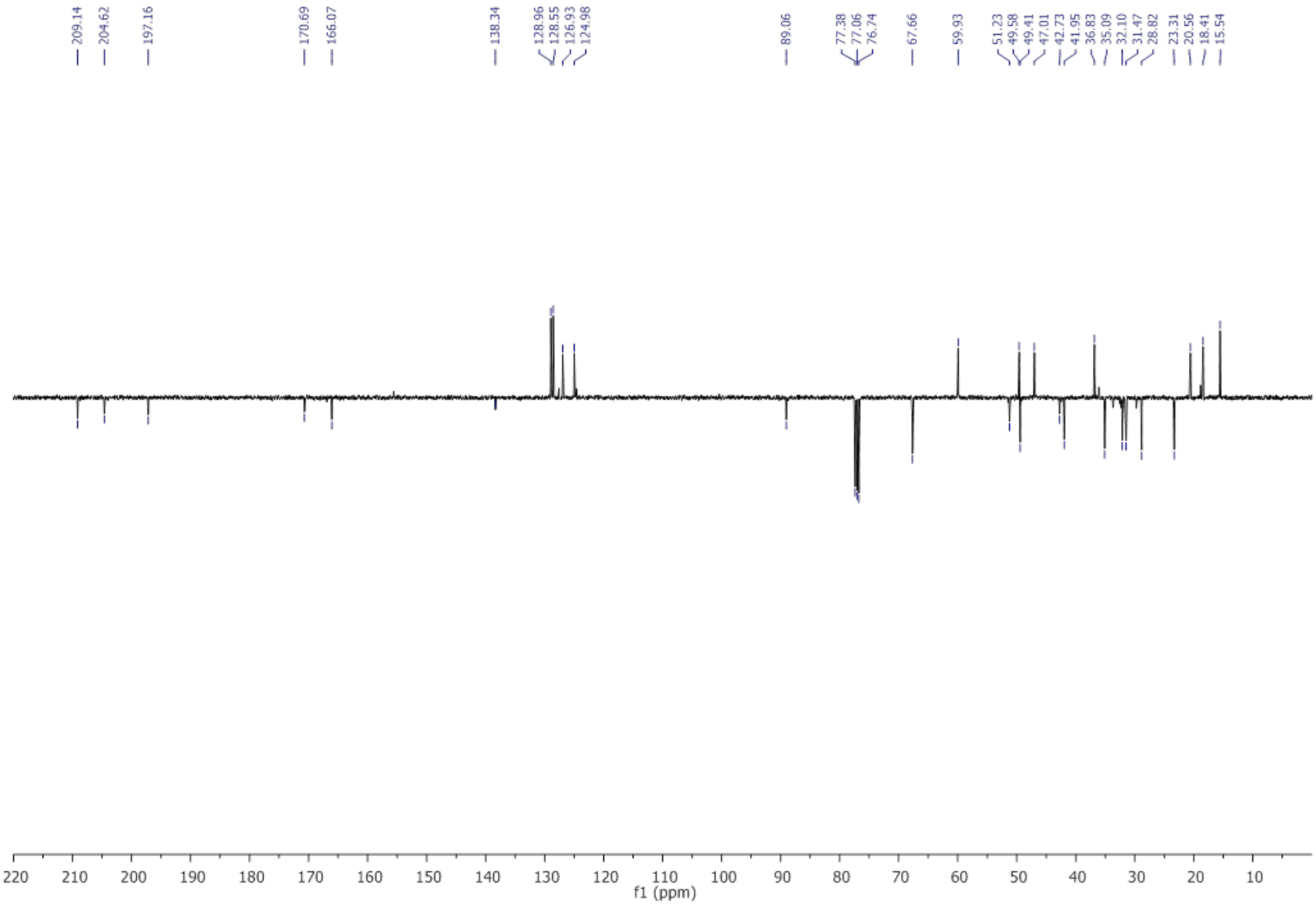

209.14
204.62
197.16
170.69
166.07
138.34
128.96
128.55
126.93
124.98
89.06
77.38
77.06
76.74
67.66
59.93
51.23
49.58
49.41
47.01
42.73
41.95
36.83
35.09
32.10
31.47
28.82
23.31
20.56
18.41
15.54
220 210 200 190 180 170 160 150 140 130 120 110 100 90 80 70 60 50 40 30 20 10
f1 (ppm)

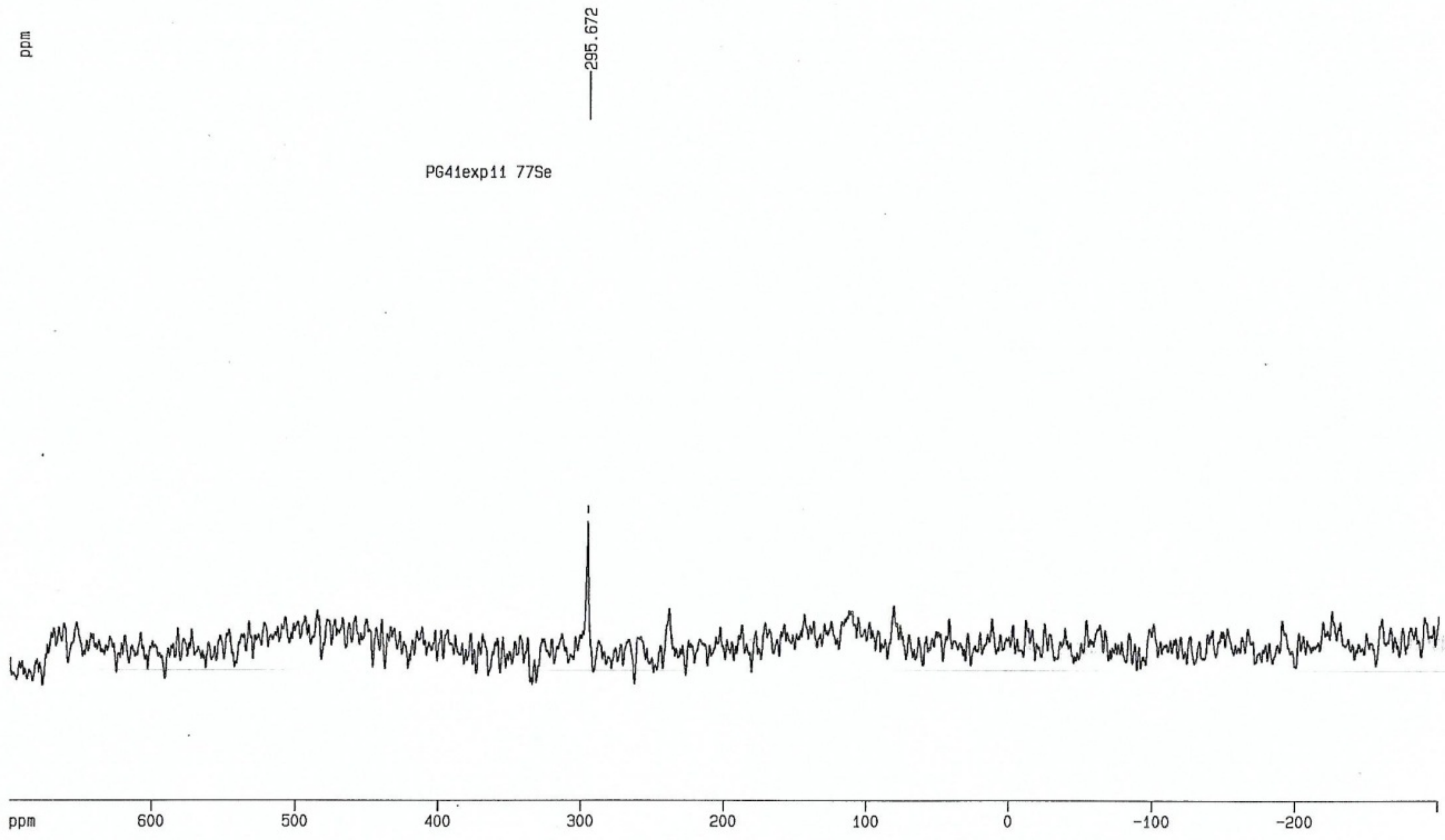
ppm
295.672
PG41exp11 77Se
ppm 600 500 400 300 200 100 0 −100 −200

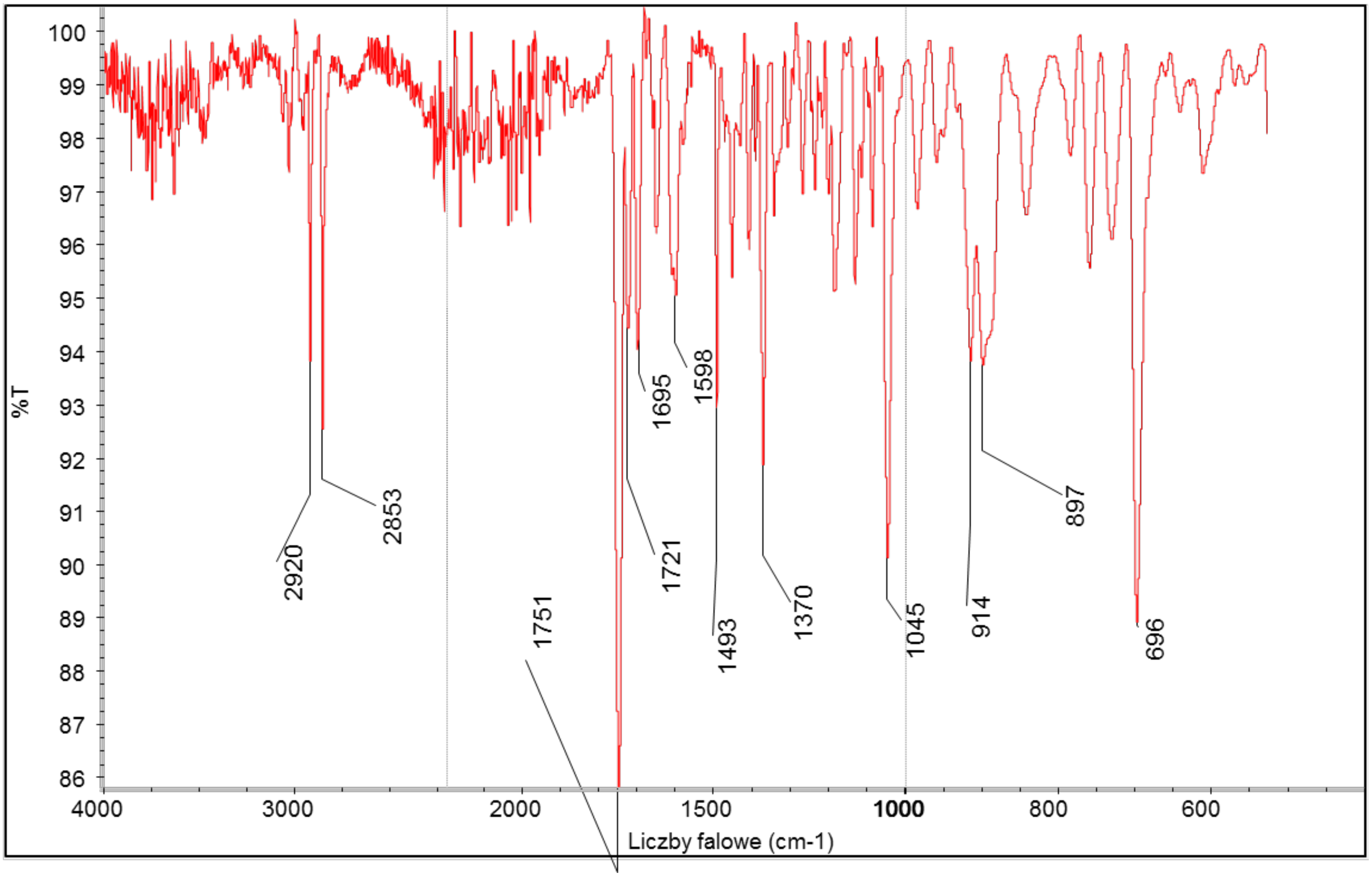

%T
100
99
98
97
96
95
94
93
92
91
90
89
88
87
86
4000
3000
2000
1500
1000
800
600
Liczby falowe (cm-1)
2920
2853
1751
1721
1695
1598
1493
1370
1045
914
897
696

**Sample Name**	PG41	**Position**	P1-B1	**Instrument Name**	Instrument 1
**User Name**		**Inj Vol**	0.1	**InjPosition**	
**Sample Type**	Sample	**IRM Calibration Status**	Success	**Data Filename**	6min_MS 2-6minMeOH.m PG41 V=0.1.d
**ACQ Method**	6min_MS 2-6minMeOH.m	**Comment**		**Acquired Time**	7/8/2019 8:03:45 PM

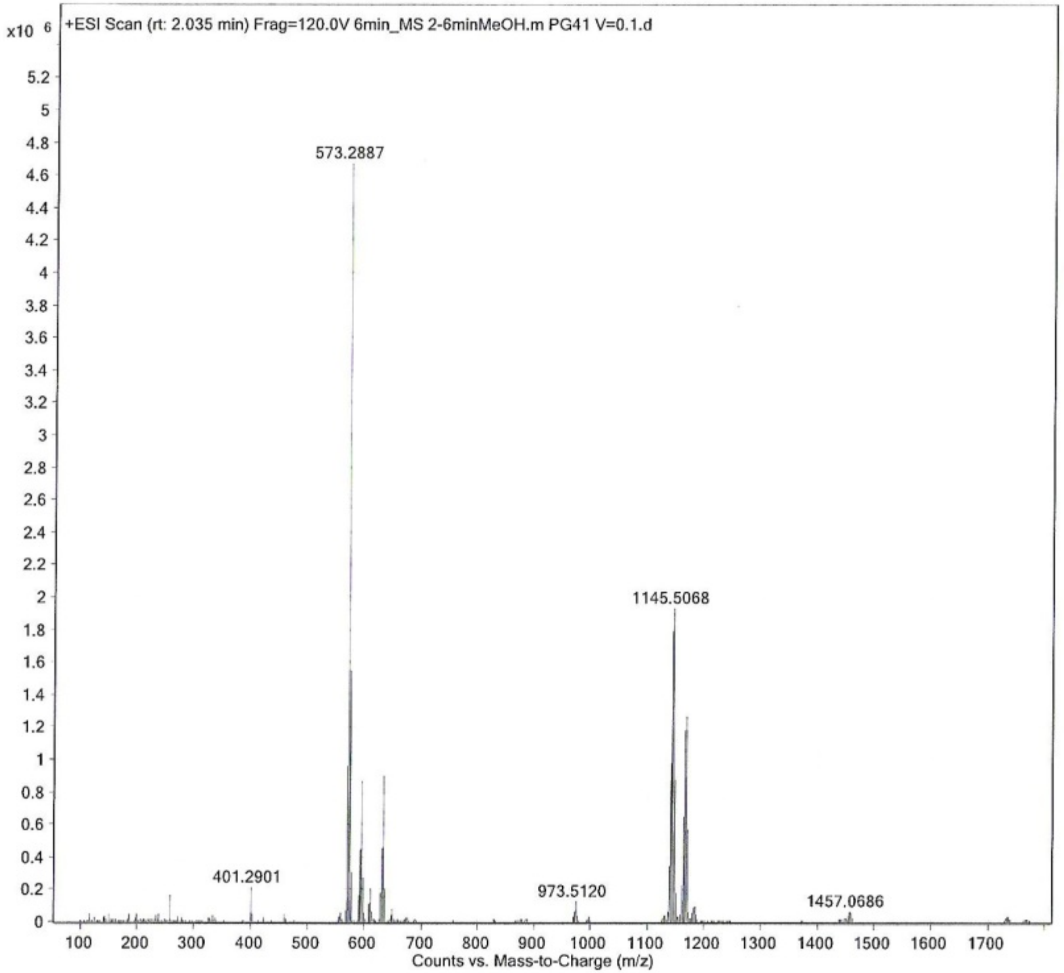

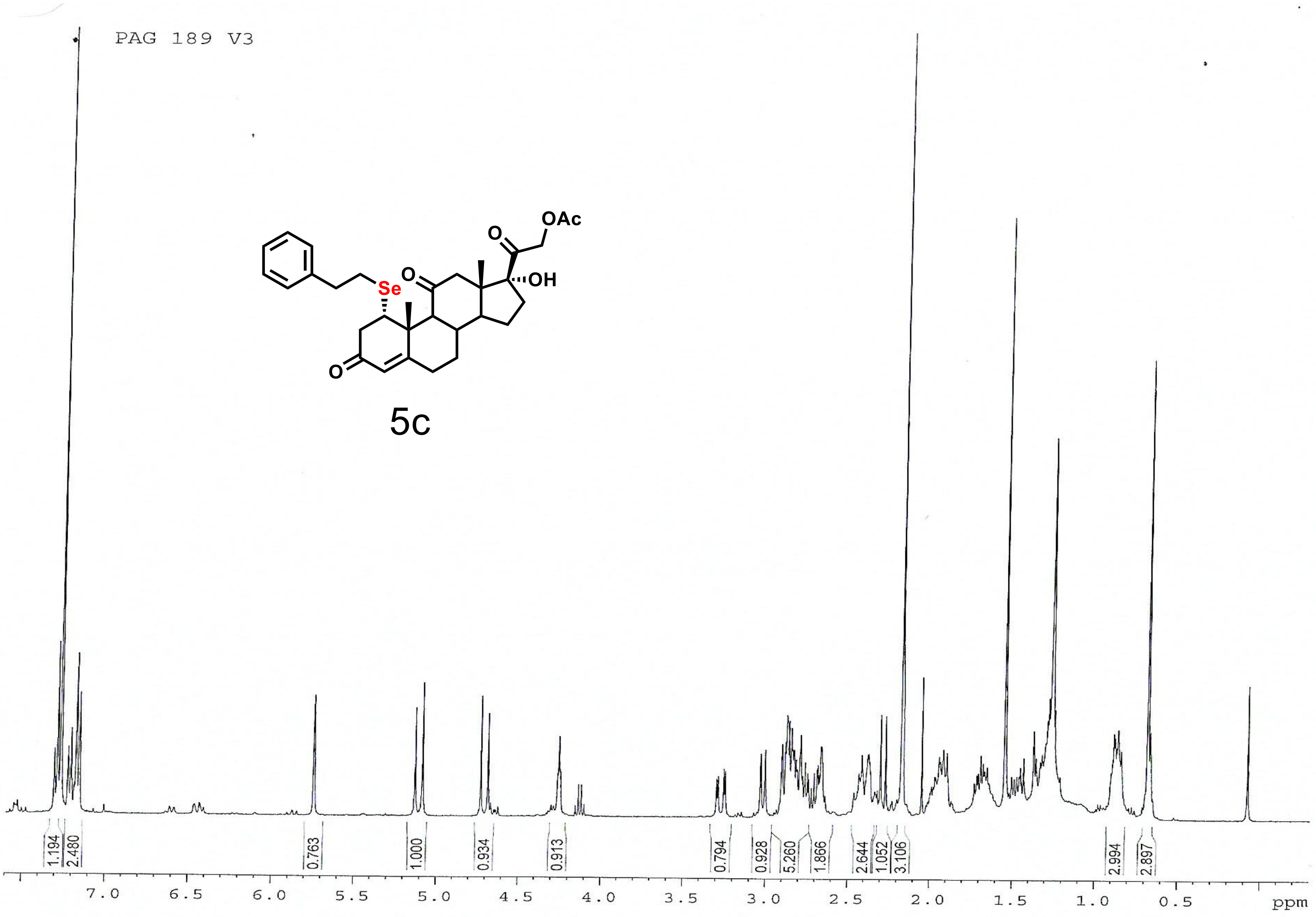

PAG 189 V3
OAc
O
OH
Se
O
O
5c
1.194
2.480
0.763
1.000
0.934
0.913
0.794
0.928
5.260
1.866
2.644
1.052
3.106
2.994
2.897
7.0
6.5
6.0
5.5
5.0
4.5
4.0
3.5
3.0
2.5
2.0
1.5
1.0
0.5
ppm

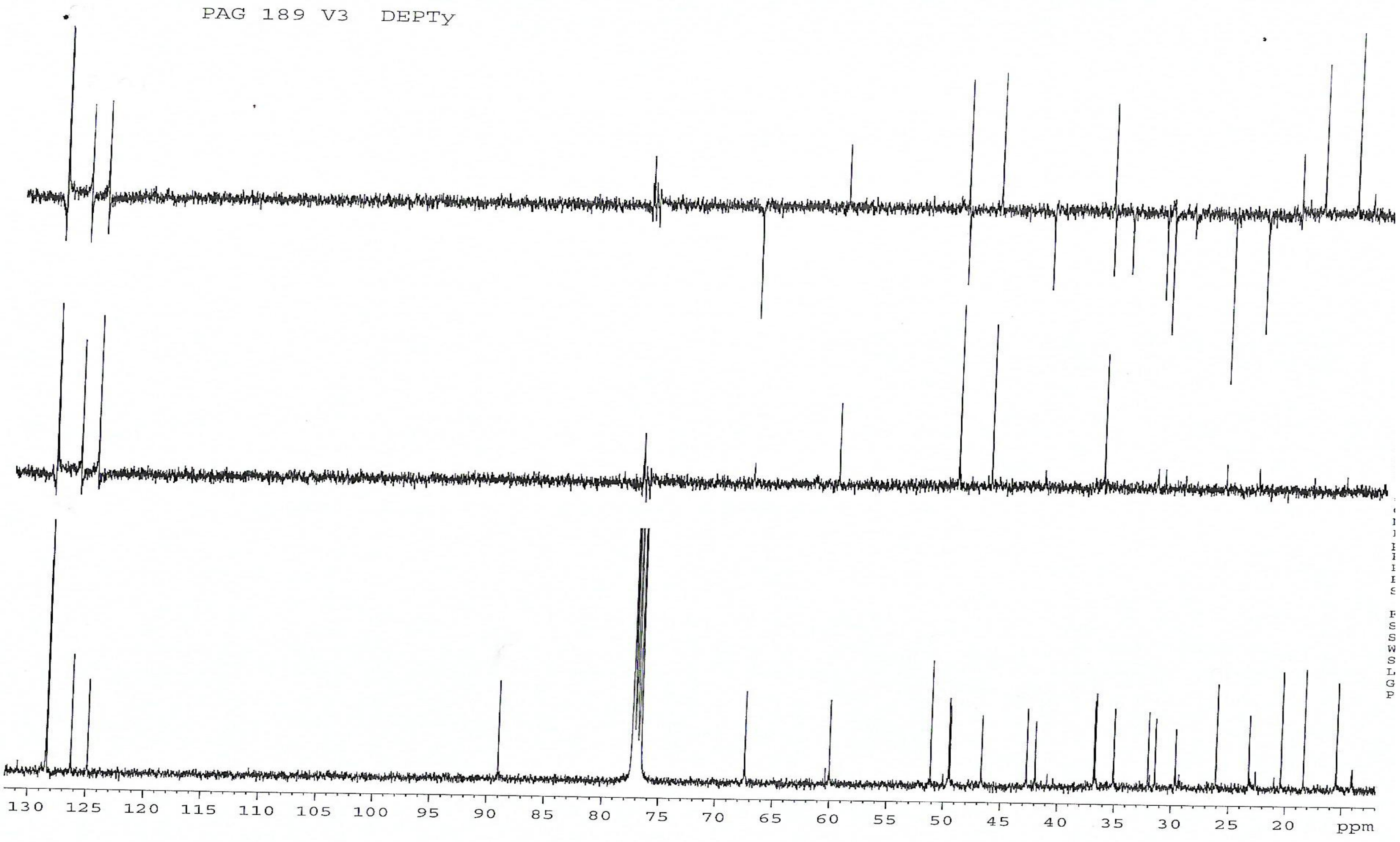

PAG 189 V3 DEPTy
130 125 120 115 110 105 100 95 90 85 80 75 70 65 60 55 50 45 40 35 30 25 20 ppm

PAG 189 V3 77Se

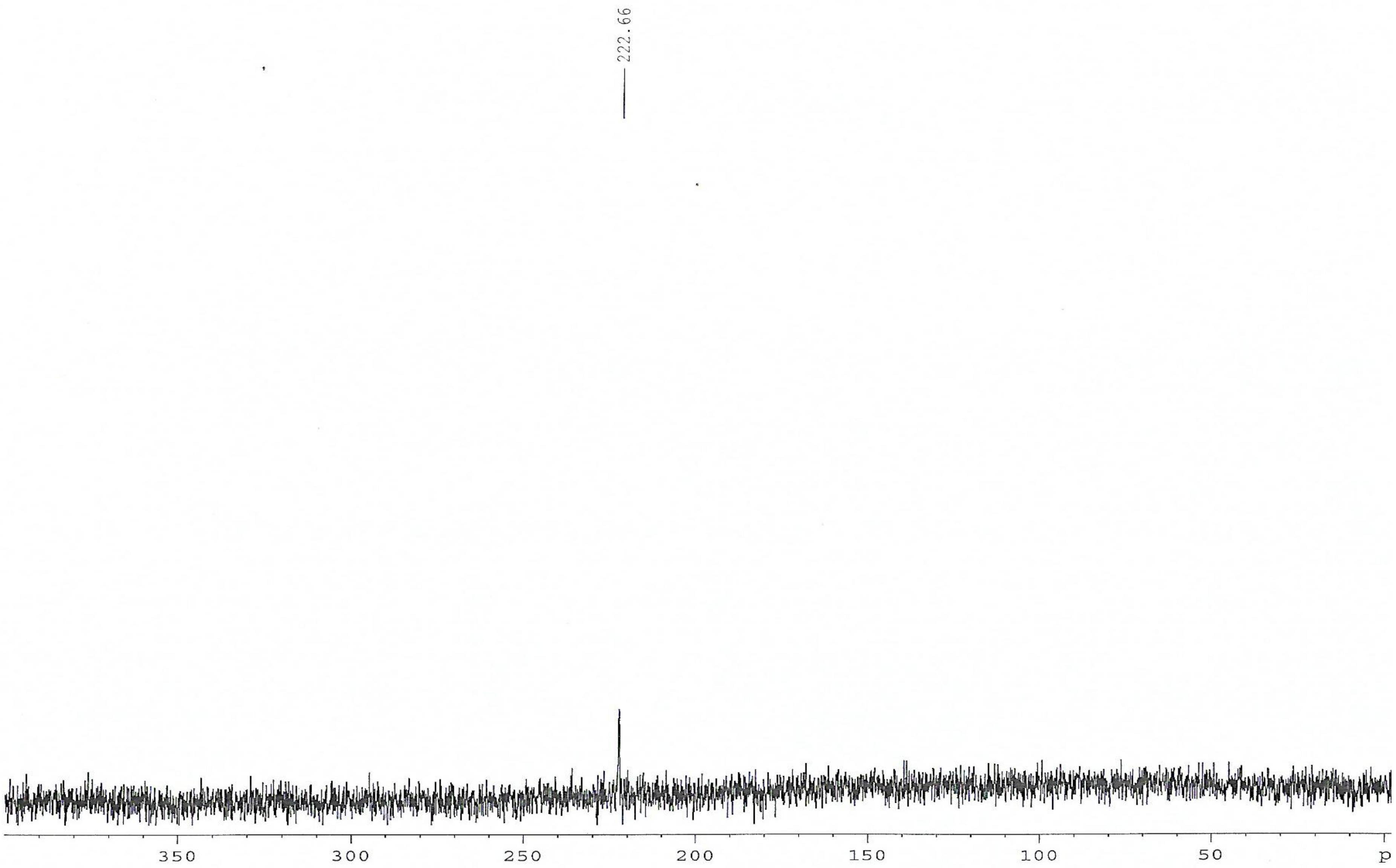

**Sample Name**	PG42	**Position**	P1-B1	**Instrument Name**	Instrument 1
**User Name**		**Inj Vol**	0.1	**InjPosition**	
**Sample Type**	Sample	**IRM Calibration Status**	Success	**Data Filename**	6min_MS 2-6minMeOH.m PG42.d
**ACQ Method**	6min_MS 2-6minMeOH.m	**Comment**		**Acquired Time**	7/16/2019 3:12:10 PM

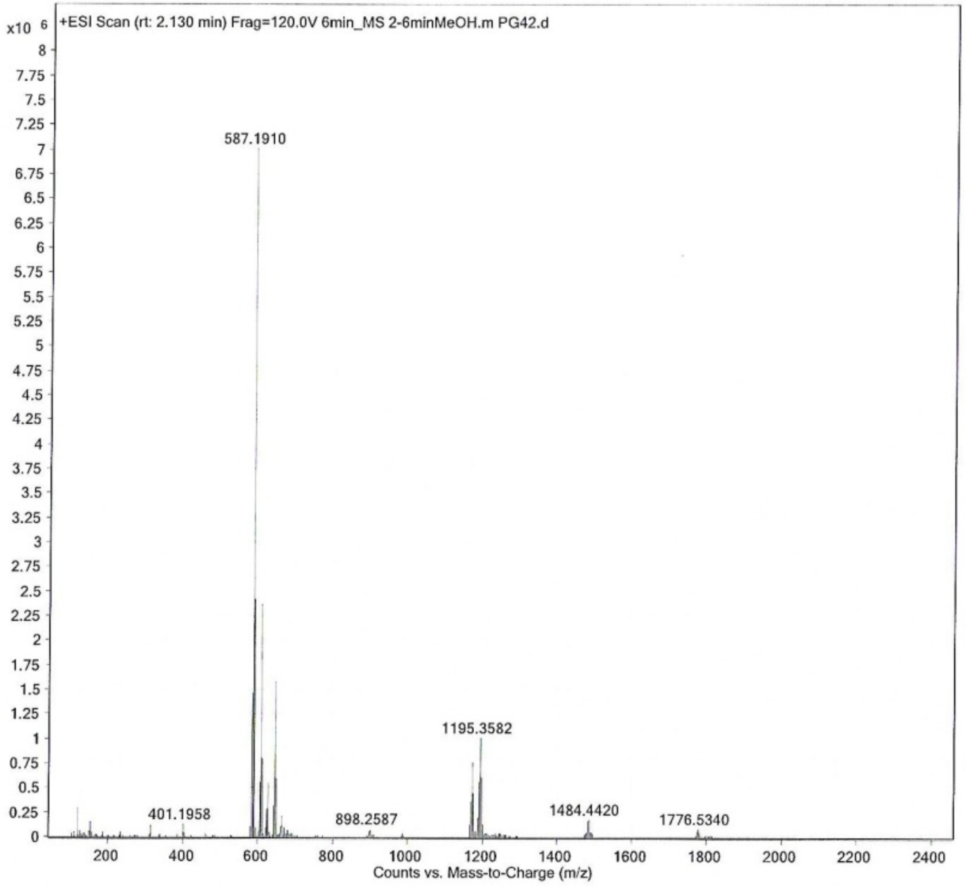

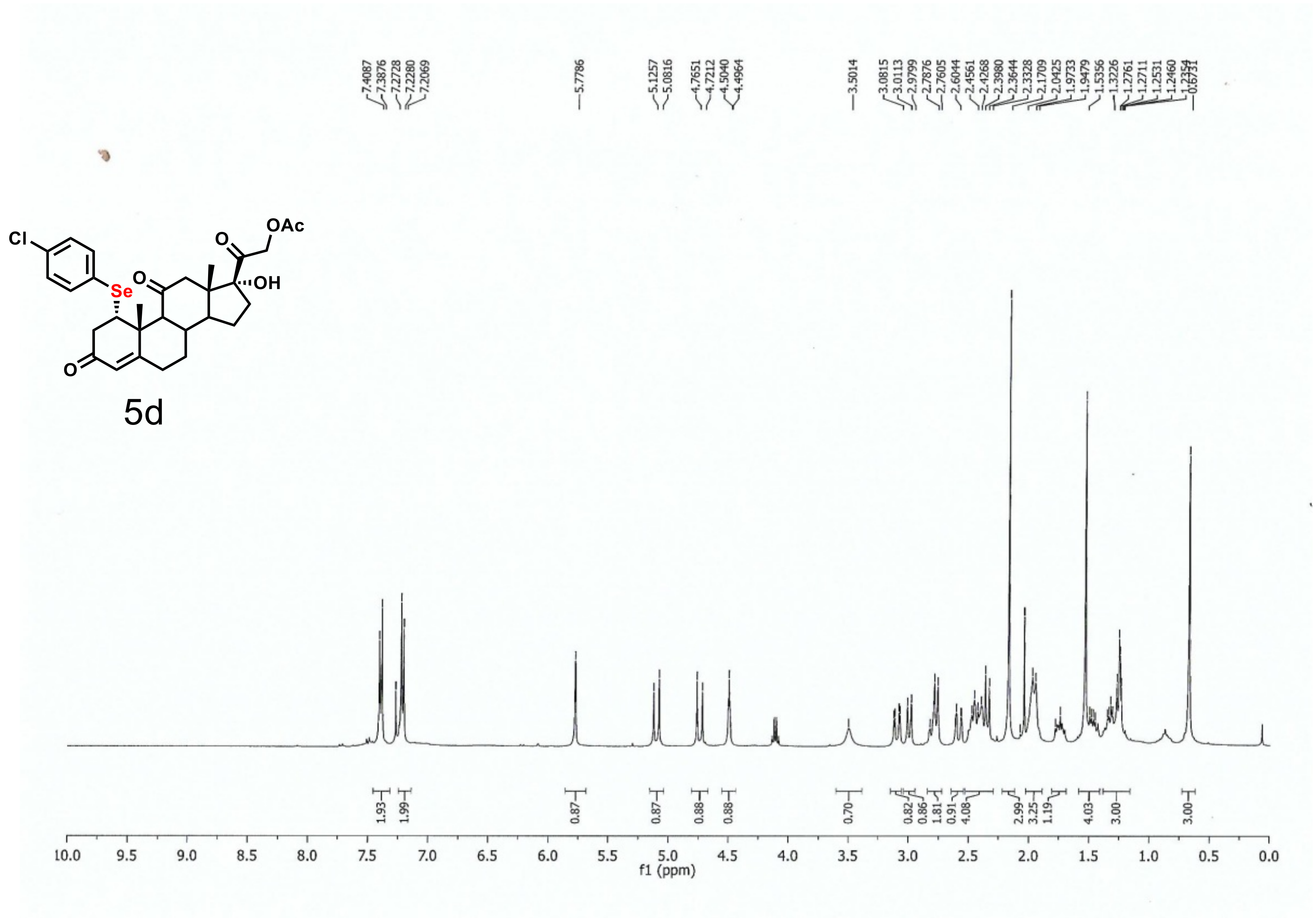

Cl
Se
O
OAc
OH
7.4087
7.3876
7.2728
7.2280
7.2069
5.7786
5.1257
5.0816
4.7651
4.7212
4.5040
4.4964
3.5014
3.0815
3.0113
2.9799
2.7876
2.7605
2.6044
2.4561
2.4268
2.3980
2.3644
2.3328
2.1709
2.0425
1.9733
1.9479
1.5356
1.3226
1.2761
1.2711
1.2531
1.2460
1.2354
0.6731
1.93
1.99
0.87
0.87
0.88
0.88
0.70
0.82
0.86
1.81
0.91
4.08
2.99
3.25
1.19
4.03
3.00
3.00
f1 (ppm)

5d

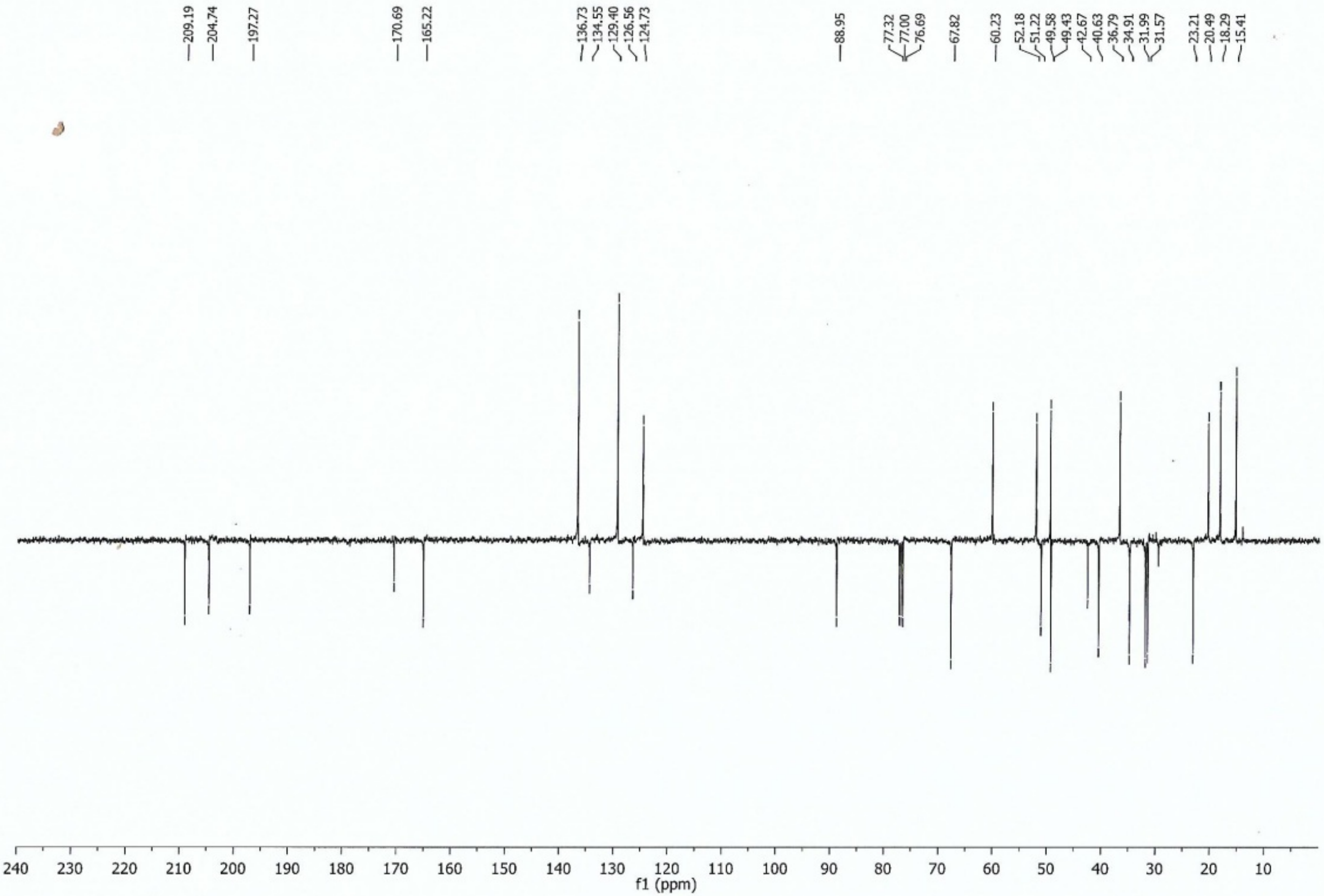

209.19
204.74
197.27
170.69
165.22
136.73
134.55
129.40
126.56
124.73
88.95
77.32
77.00
76.69
67.82
60.23
52.18
51.22
49.58
49.43
42.67
40.63
36.79
34.91
31.99
31.57
23.21
20.49
18.29
15.41
240 230 220 210 200 190 180 170 160 150 140 130 120 110 100 90 80 70 60 50 40 30 20 10
f1 (ppm)

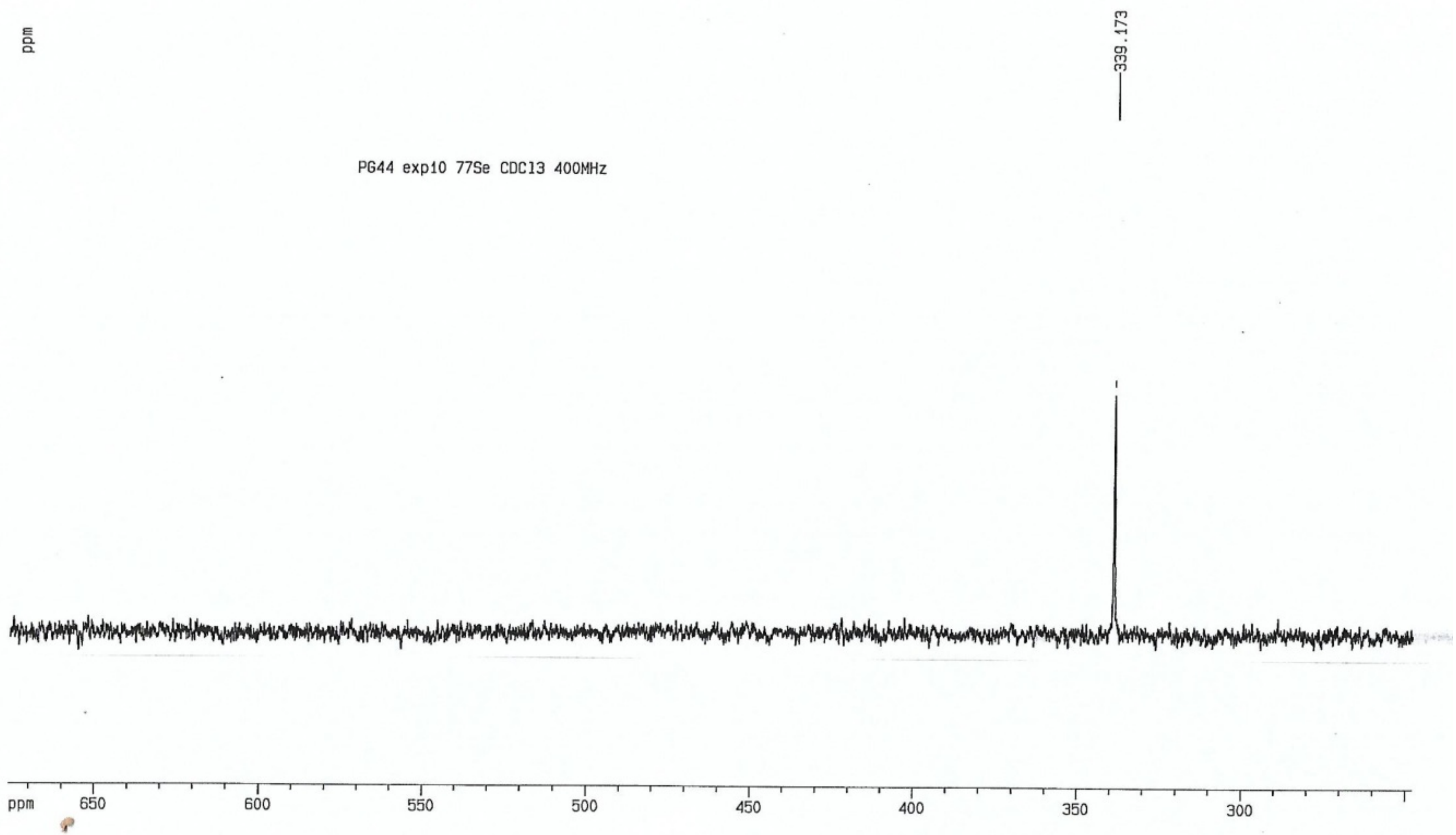
ppm
339.173
PG44 exp10 77Se CDCl3 400MHz
ppm 650 600 550 500 450 400 350 300

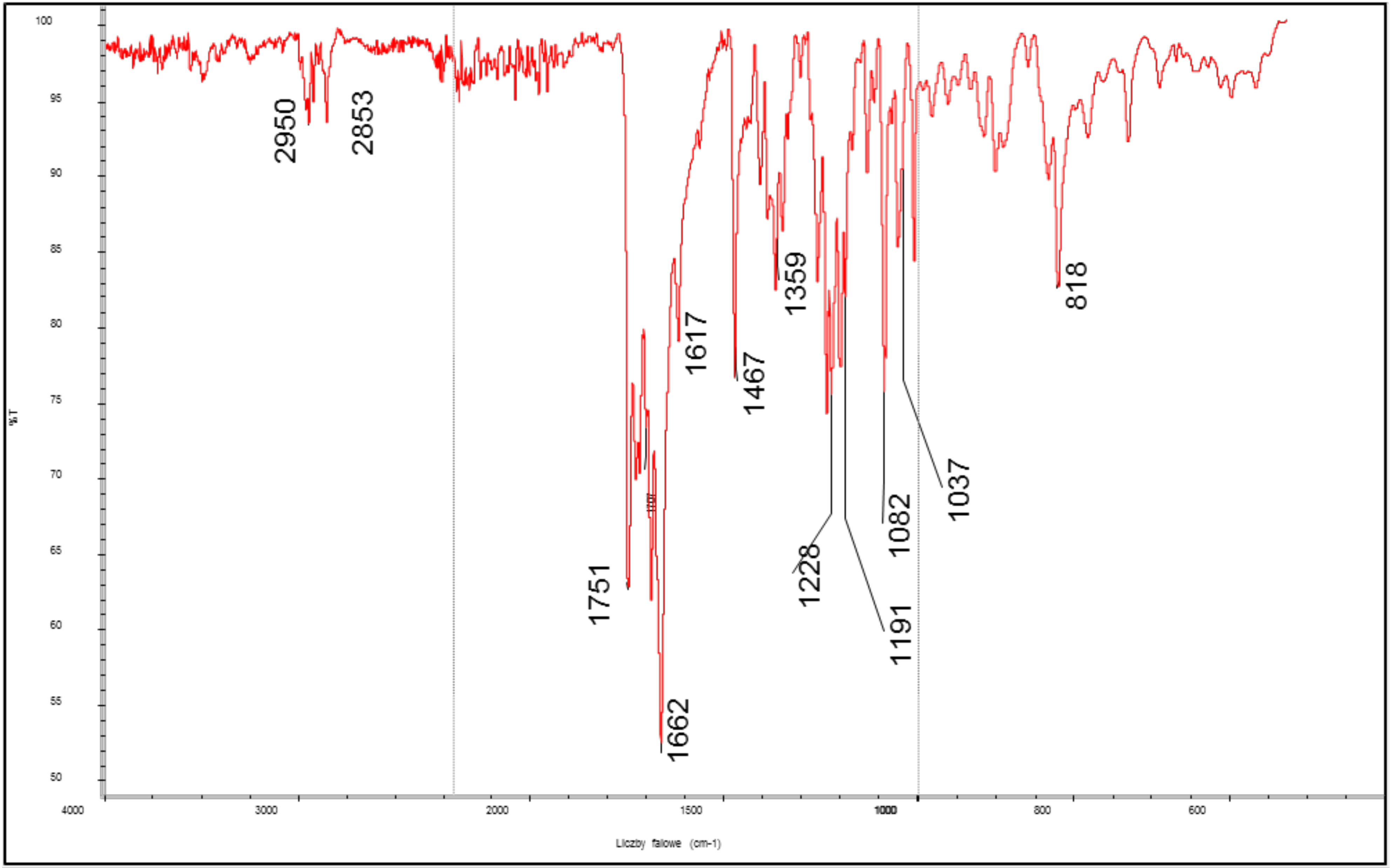

100
95
90
85
80
75
70
65
60
55
50
%T
2950
2853
1751
1662
1617
1467
1359
1228
1191
1082
1037
818
4000
3000
2000
1500
1000
800
600
Liczby falowe (cm-1)

**Sample Name**	PG44	**Position**	P1-B1	**Instrument Name**	Instrument 1
**User Name**		**Inj Vol**	0.1	**InjPosition**	
**Sample Type**	Sample	**IRM Calibration Status**	Success	**Data Filename**	6min_MS 2-6minMeOH.m PG44.d
**ACQ Method**	6min_MS 2-6minMeOH.m	**Comment**		**Acquired Time**	7/17/2019 1:31:35 PM

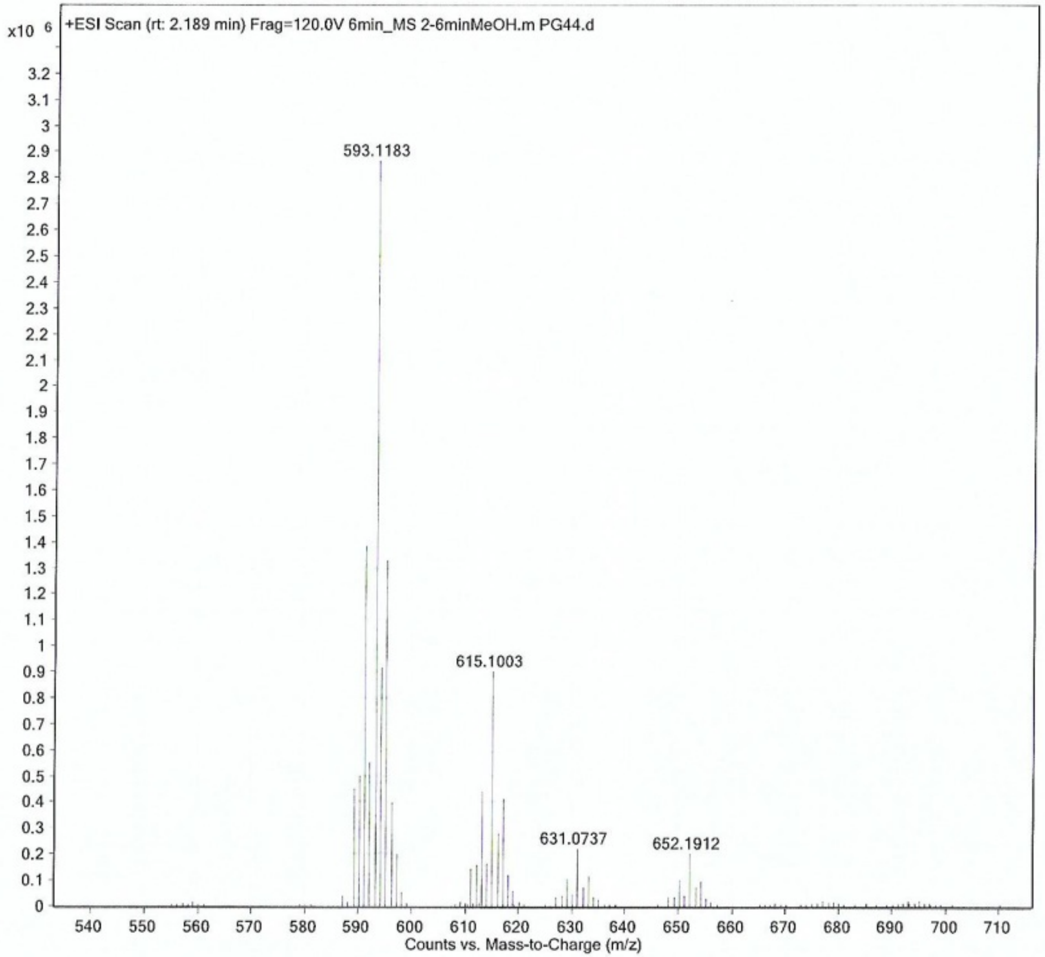

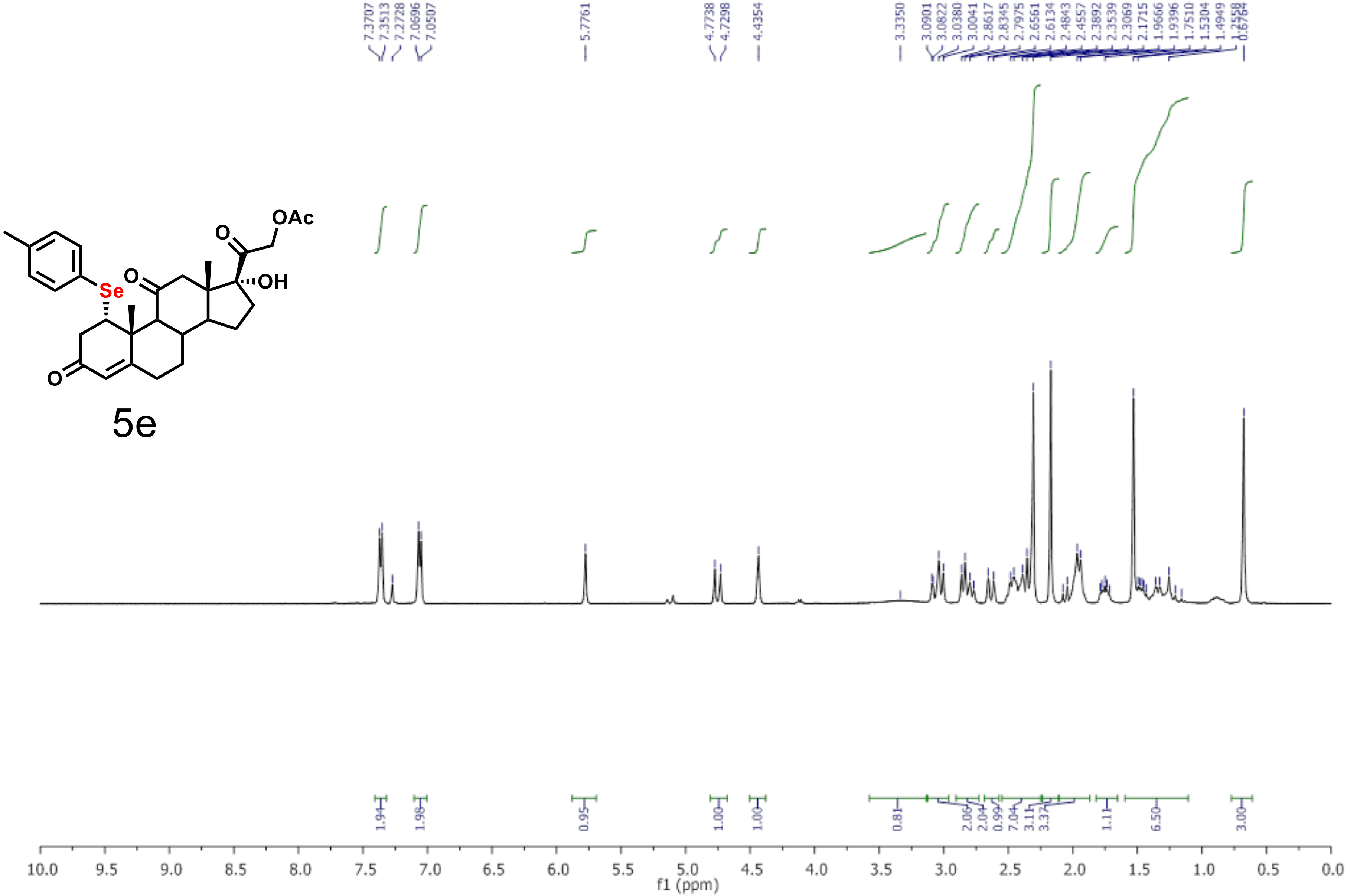
OAc
O
O
OH
Se
O
7.3707
7.3513
7.2728
7.0696
7.0507
5.7761
4.7738
4.7298
4.4354
3.3350
3.0901
3.0822
3.0380
3.0041
2.8617
2.8345
2.7975
2.6561
2.6134
2.4843
2.4557
2.3892
2.3539
2.3069
2.1715
1.9666
1.9396
1.7510
1.5304
1.4949
1.2558
0.6764
1.94
1.98
0.95
1.00
1.00
0.81
2.06
2.04
0.99
7.04
3.11
3.37
1.11
6.50
3.00
10.0
9.5
9.0
8.5
8.0
7.5
7.0
6.5
6.0
5.5
5.0
4.5
4.0
3.5
3.0
2.5
2.0
1.5
1.0
0.5
0.0
f1 (ppm)

5e

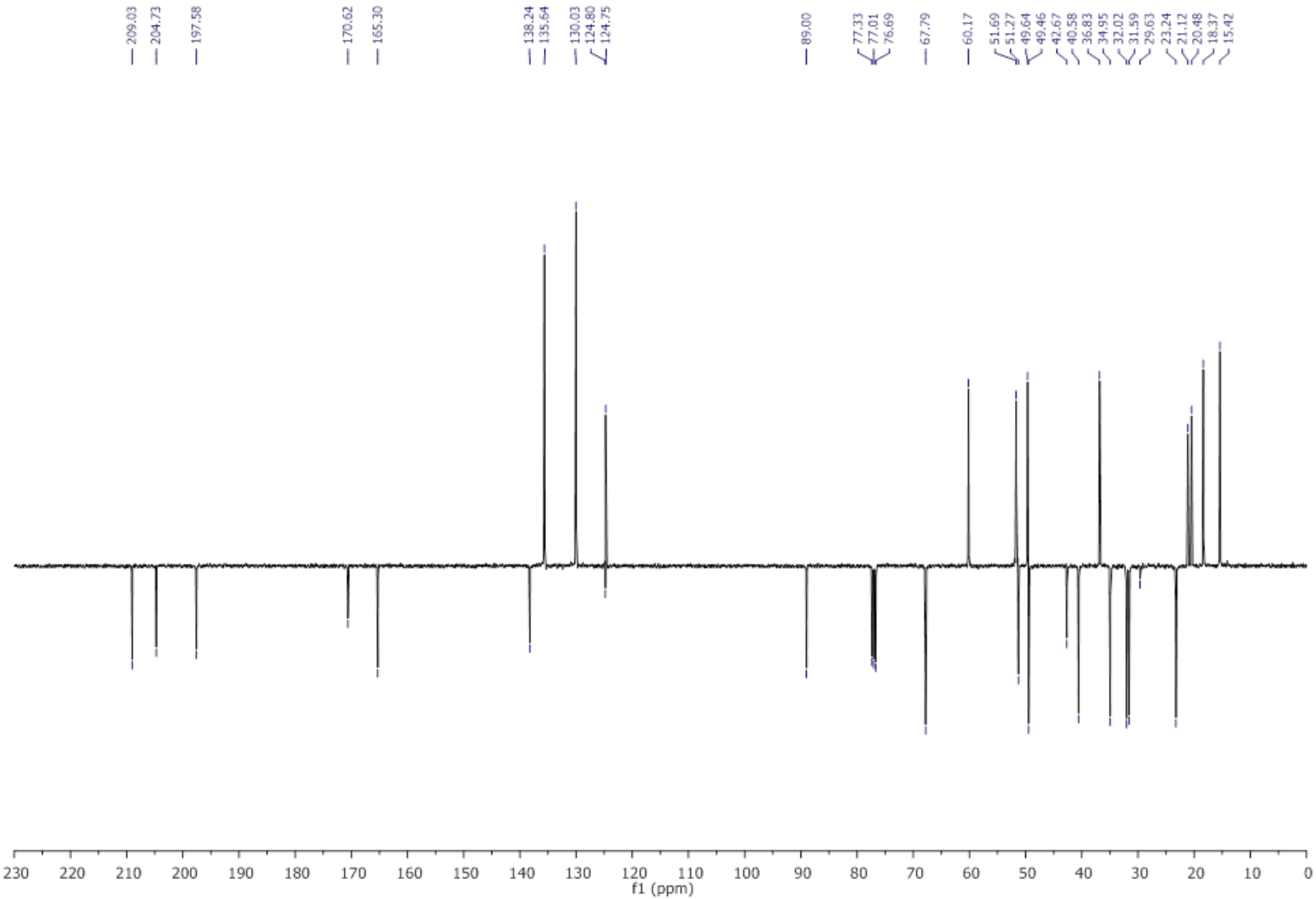
209.03
204.73
197.58
170.62
165.30
138.24
135.64
130.03
124.80
124.75
89.00
77.33
77.01
76.69
67.79
60.17
51.69
51.27
49.64
49.46
42.67
40.58
36.83
34.95
32.02
31.59
29.63
23.24
21.12
20.48
18.37
15.42
230 220 210 200 190 180 170 160 150 140 130 120 110 100 90 80 70 60 50 40 30 20 10 0
f1 (ppm)

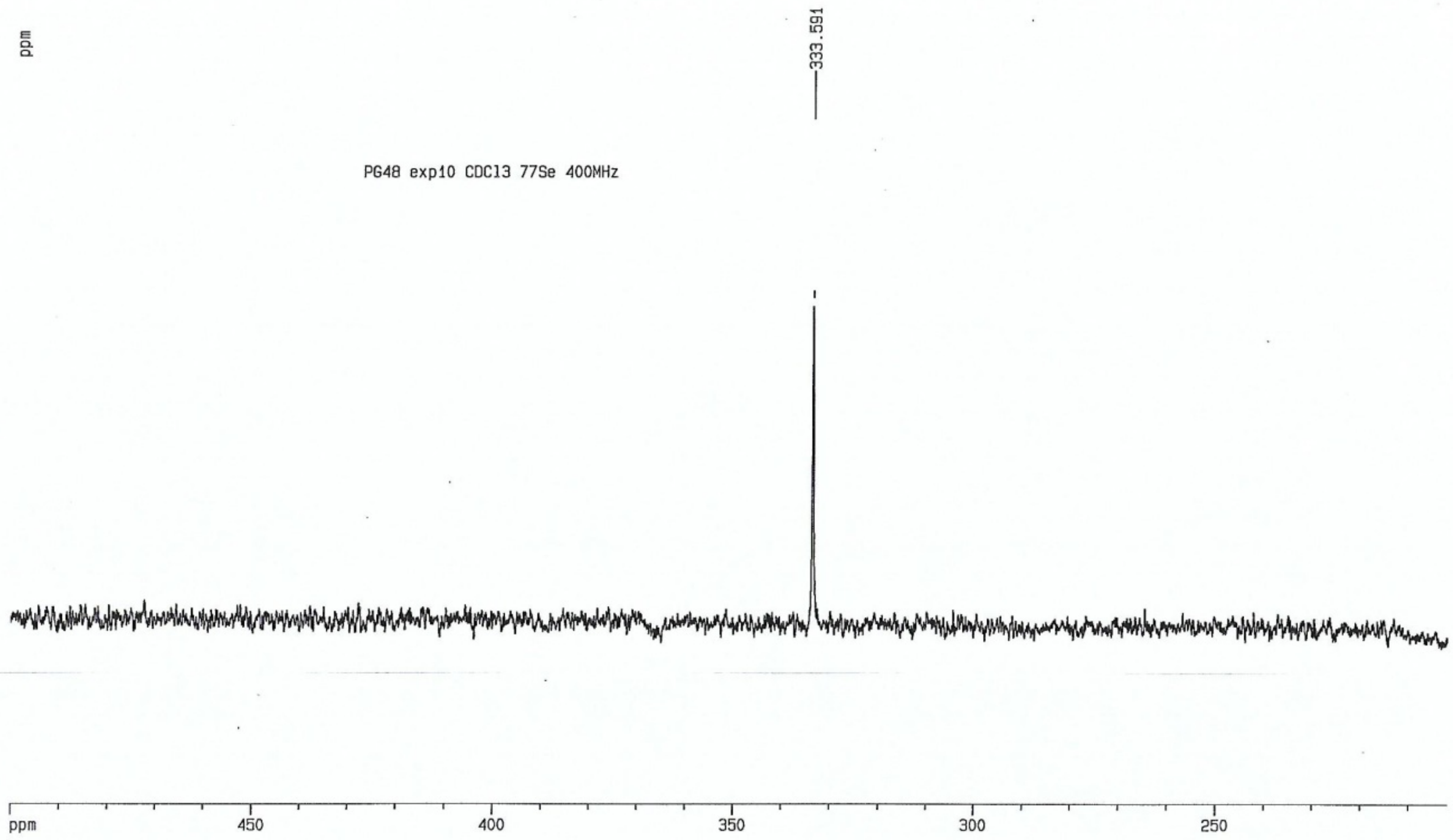
ppm
333.591
PG48 exp10 CDCl3 77Se 400MHz
ppm
450
400
350
300
250

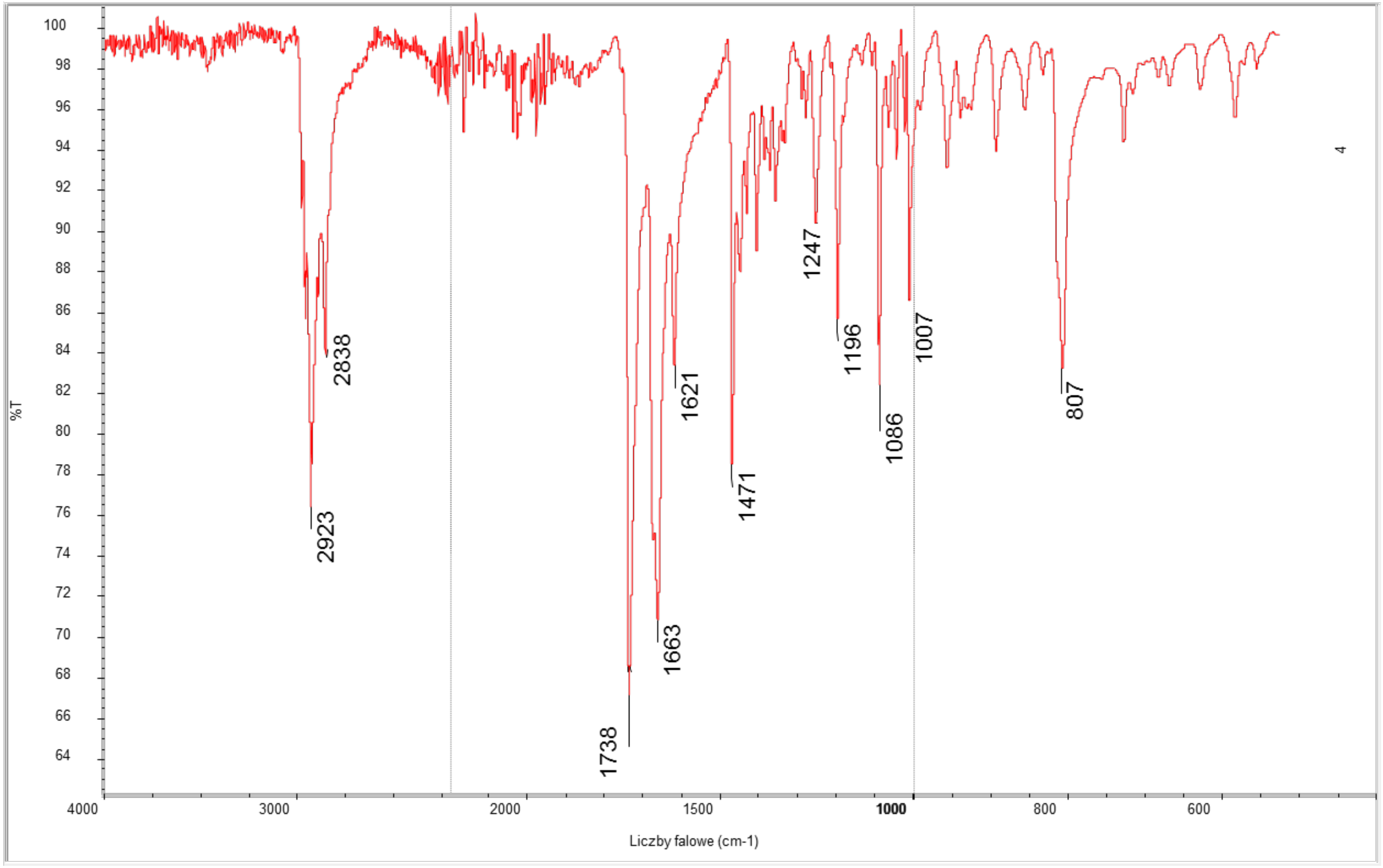

4
%T
100
98
96
94
92
90
88
86
84
82
80
78
76
74
72
70
68
66
64
2923
2838
1738
1663
1621
1471
1247
1196
1086
1007
807
4000
3000
2000
1500
1000
800
600
Liczby falowe (cm-1)

**Sample Name**	PG48	**Position**	P1-B1	**Instrument Name**	Instrument 1
**User Name**		**Inj Vol**	0.1	**InjPosition**	
**Sample Type**	Sample	**IRM Calibration Status**	Success	**Data Filename**	6min_MS 2-6minMeOH.m PG48.d
**ACQ Method**	6min_MS 2-6minMeOH.m	**Comment**		**Acquired Time**	7/17/2019 2:29:06 PM

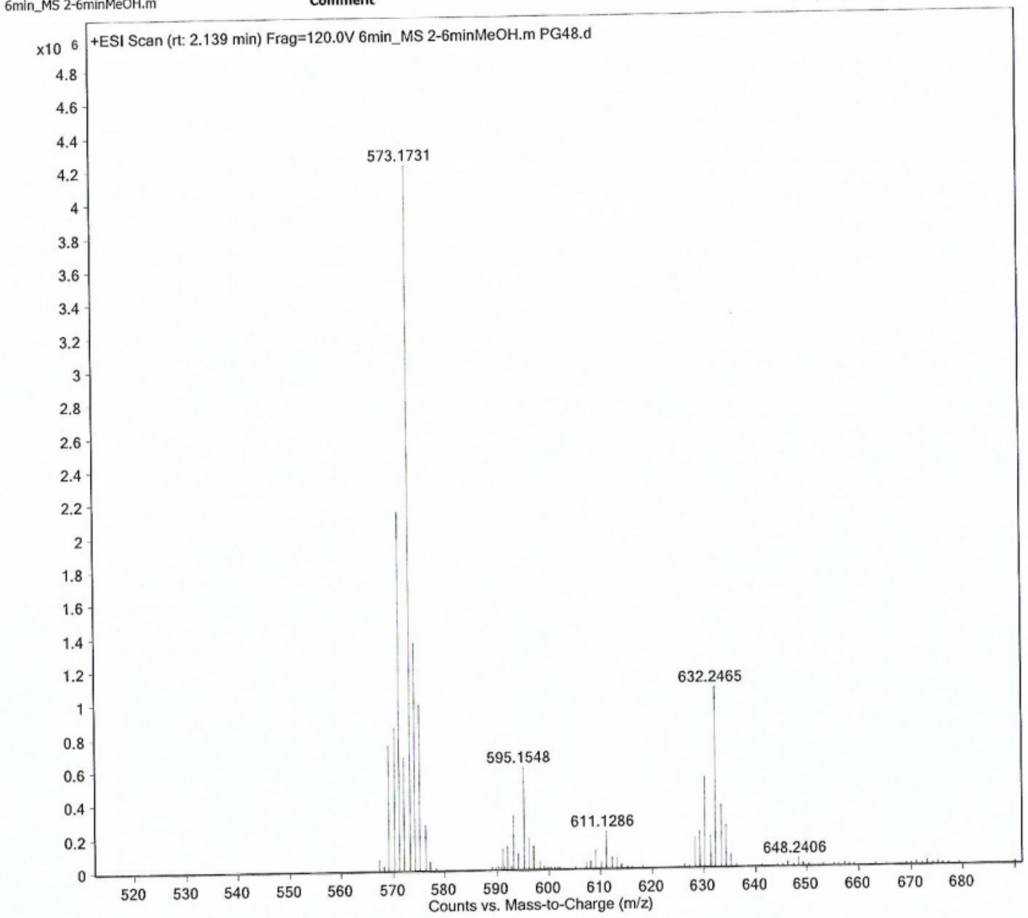

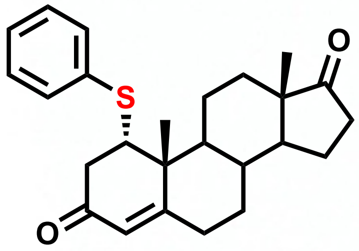

4f

BONI288 ADD + diphenyldisulfide Biphasic

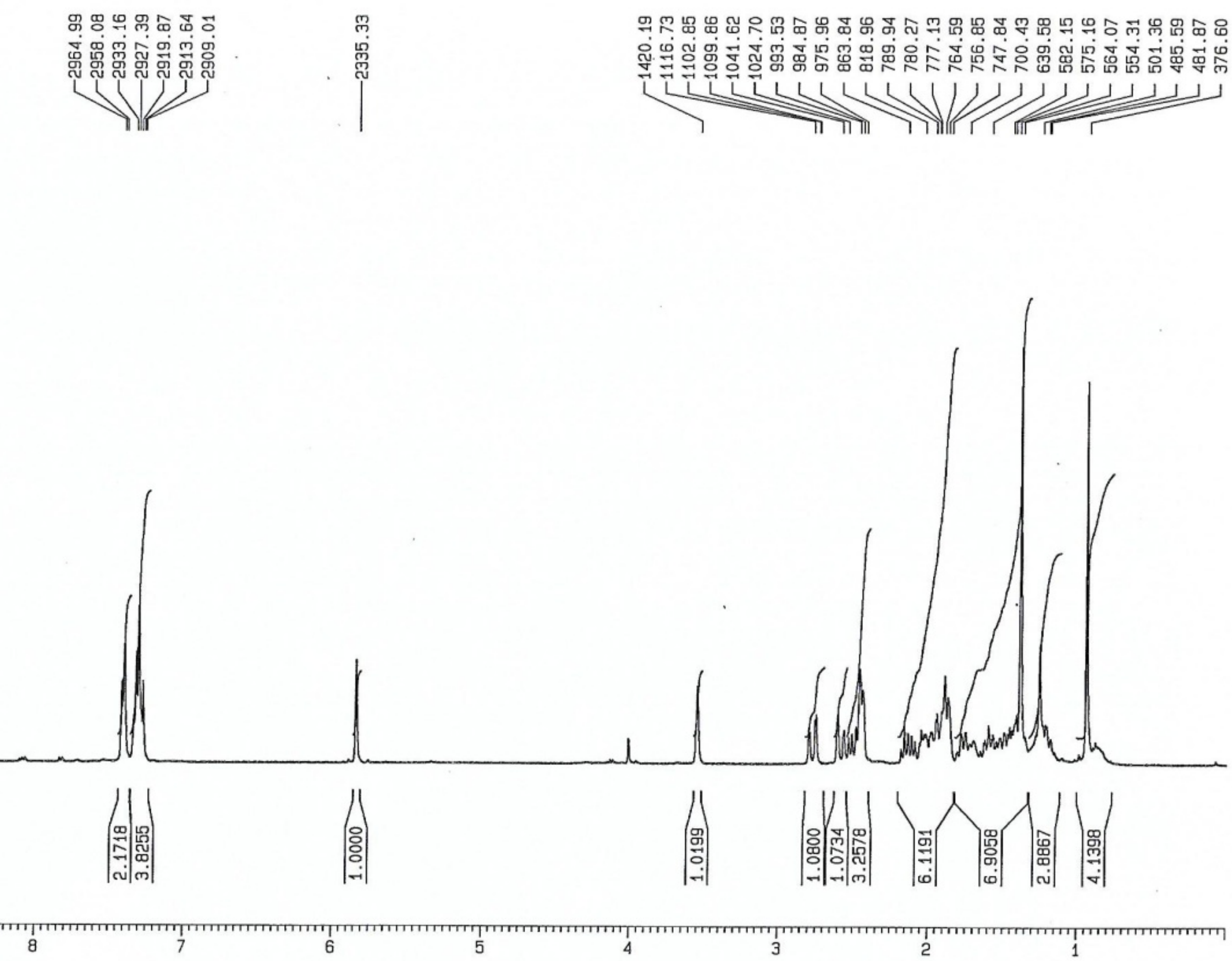

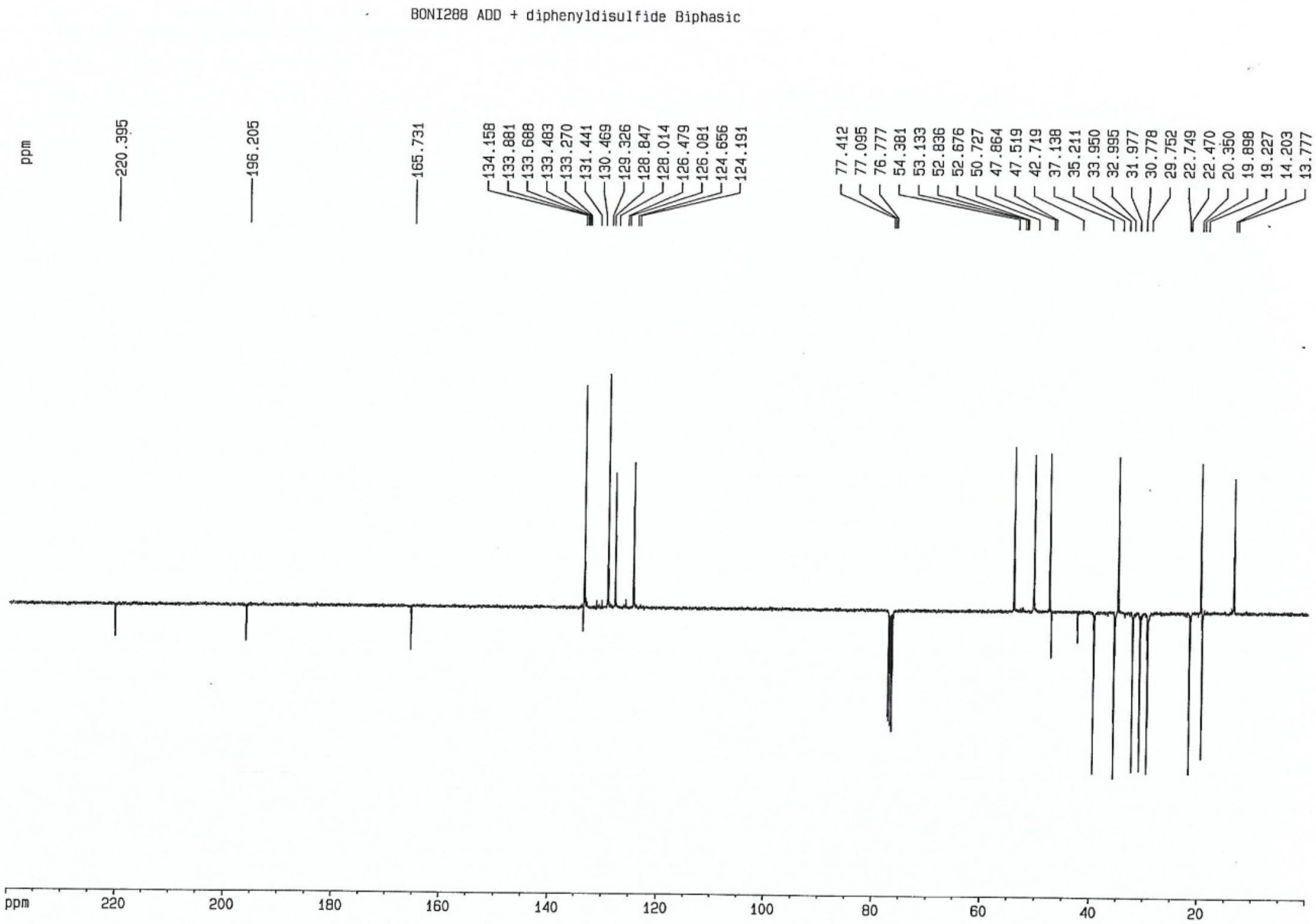
BONI288 ADD + diphenyldisulfide Biphasic
ppm
220.395
196.205
165.731
134.158
133.881
133.688
133.483
133.270
131.441
130.469
129.326
128.847
128.014
126.479
126.081
124.656
124.191
77.412
77.095
76.777
54.381
53.133
52.836
52.676
50.727
47.864
47.519
42.719
37.138
35.211
33.950
32.995
31.977
30.778
29.752
22.749
22.470
20.350
19.898
19.227
14.203
13.777
ppm
220
200
180
160
140
120
100
80
60
40
20

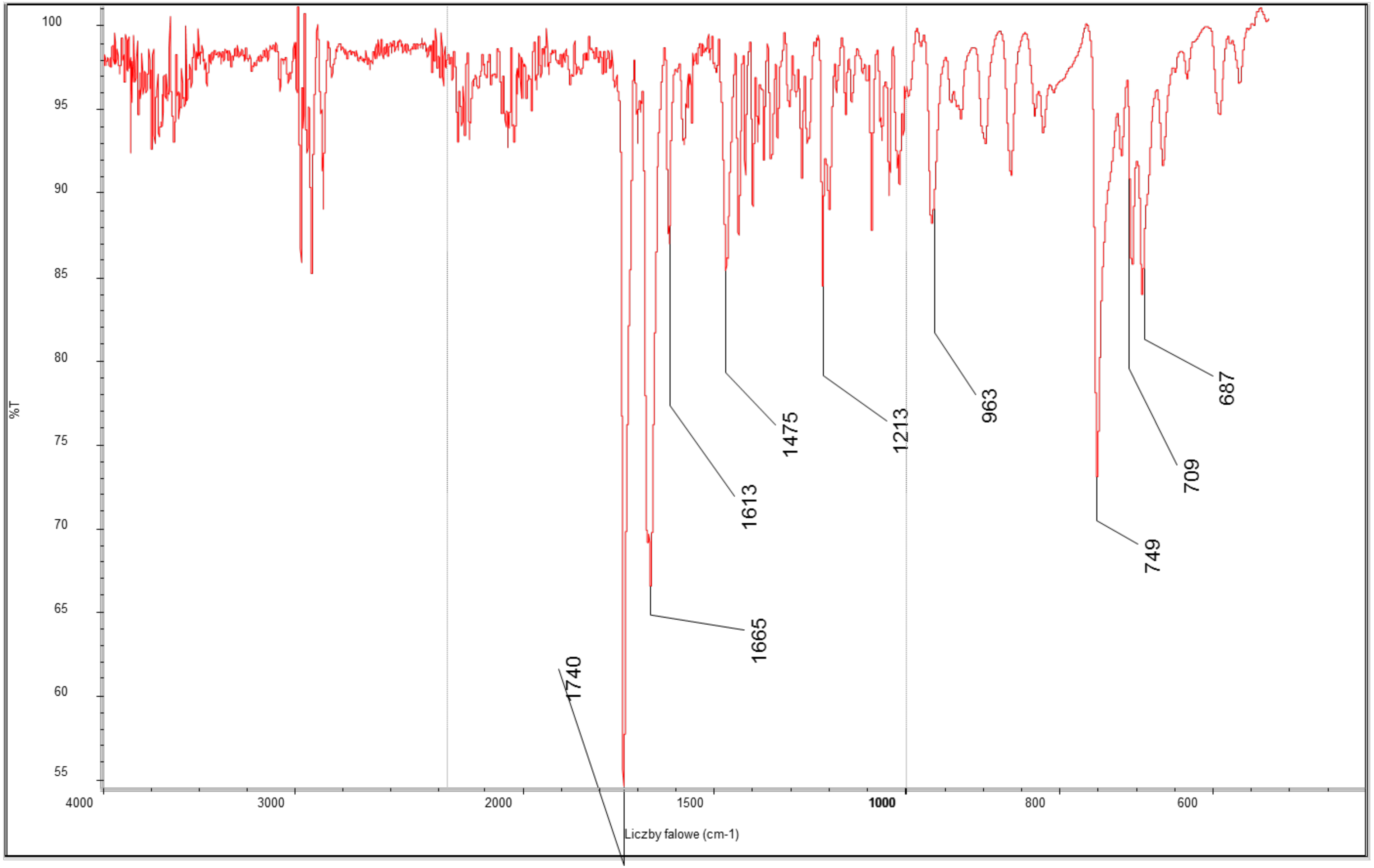

100
95
90
85
80
75
70
65
60
55
%T
4000
3000
2000
1500
1000
800
600
Liczby falowe (cm-1)
1740
1665
1613
1475
1213
963
749
709
687

**Sample Name**	Unavailable	**Position**	Unavailable	**Instrument Name**	Unavailable
**User Name**	Unavailable	**Inj Vol**	Unavailable	**InjPosition**	Unavailable
**Sample Type**	Unavailable	**IRM Calibration Status**	Success	**Data Filename**	6min_MS 2-6minMeOH.m Boni 288.d
**ACQ Method**		**Comment**	Sample information is unavailable	**Acquired Time**	Unavailable

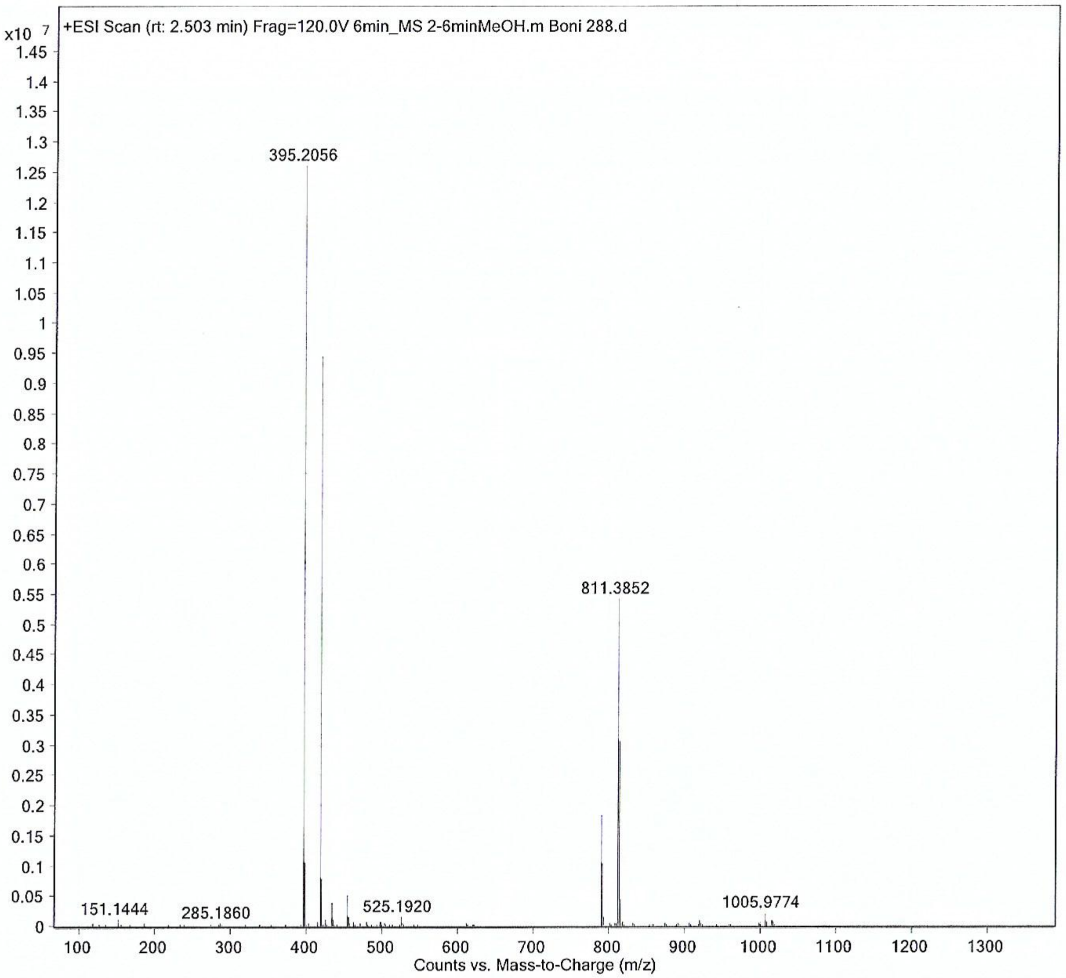

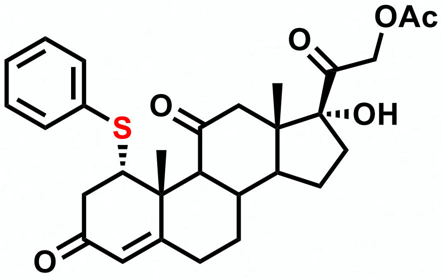

5f

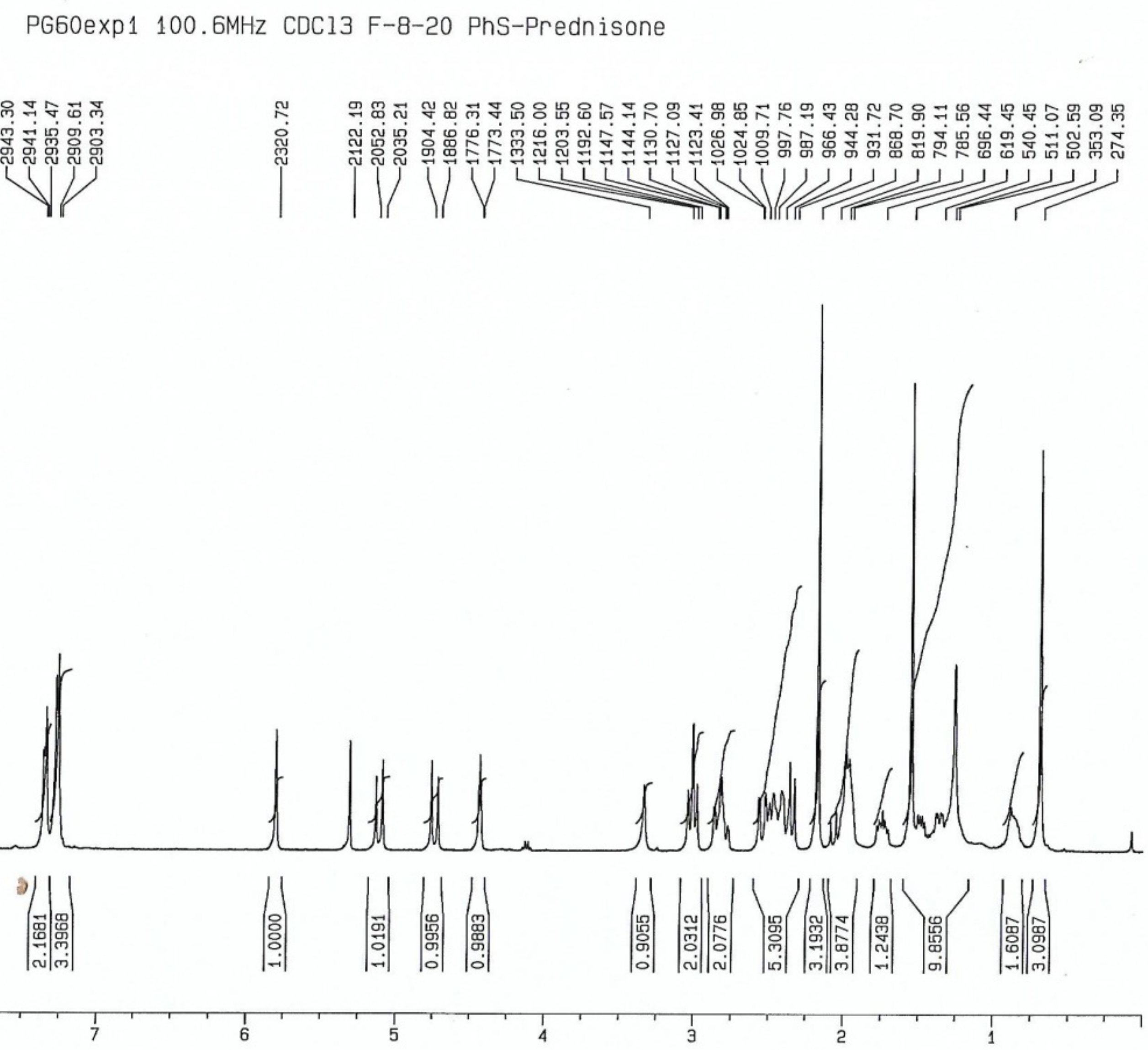

PG60exp1 100.6MHz CDCl3 F-8-20 PhS-Prednisone
Hz
2943.30 2941.14 2935.47 2909.61 2903.34 2320.72 2122.19 2052.83 2035.21 1904.42 1886.82 1776.31 1773.44 1333.50 1216.00 1203.55 1192.60 1147.57 1144.14 1130.70 1127.09 1123.41 1026.98 1024.85 1009.71 997.76 987.19 966.43 944.28 931.72 868.70 819.90 794.11 785.56 696.44 619.45 540.45 511.07 502.59 353.09 274.35
Integral
2.1681 3.3968 1.0000 1.0191 0.9956 0.9883 0.9055 2.0312 2.0776 5.3095 3.1932 3.8774 1.2438 9.8556 1.6087 3.0987
ppm
9 8 7 6 5 4 3 2 1

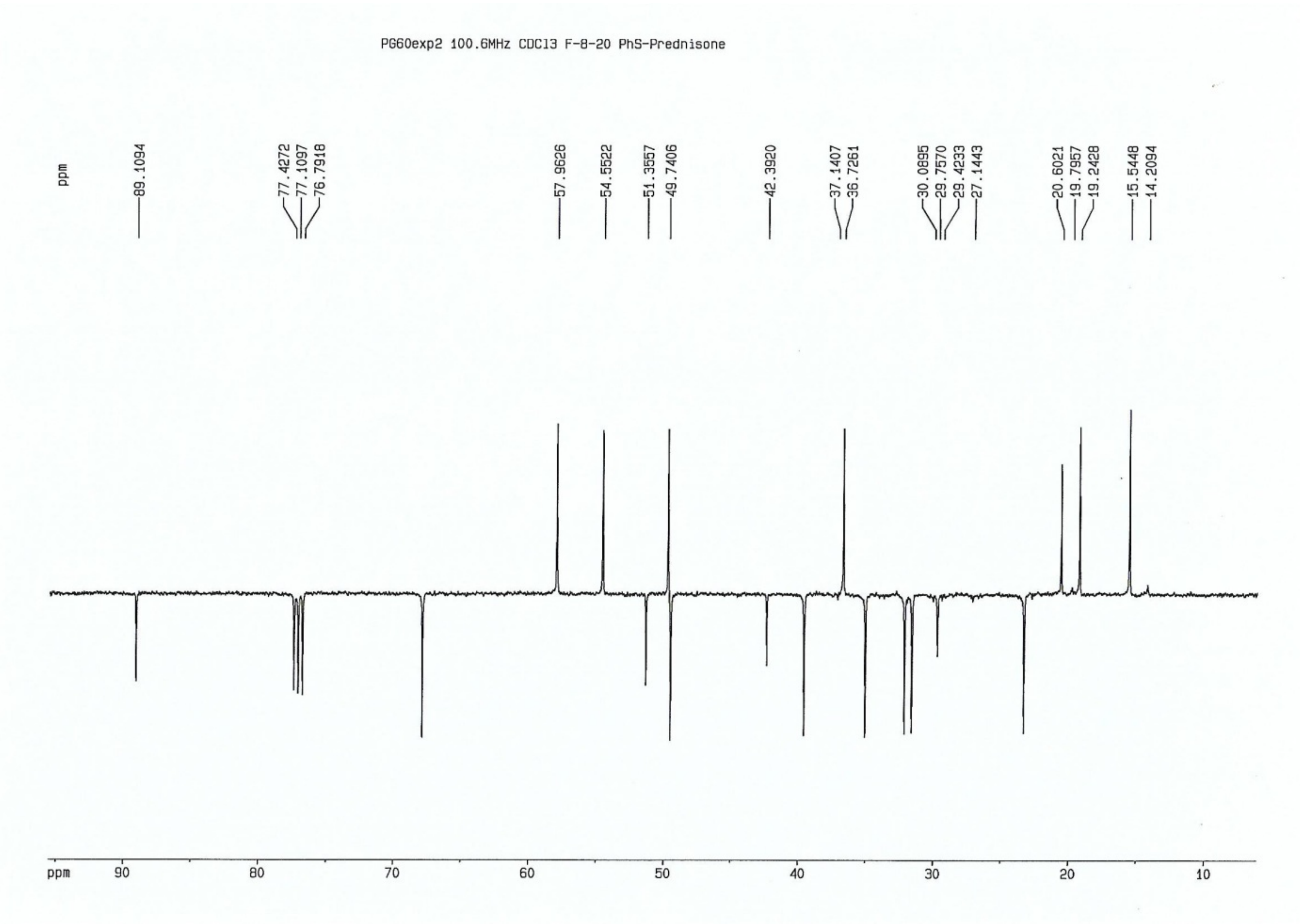
PG60exp2 100.6MHz CDCl3 F-8-20 PhS-Prednisone
ppm
89.1094
77.4272
77.1097
76.7918
57.9626
54.5522
51.3557
49.7406
42.3920
37.1407
36.7261
30.0895
29.7570
29.4233
27.1443
20.6021
19.7957
19.2428
15.5448
14.2094
ppm 90 80 70 60 50 40 30 20 10

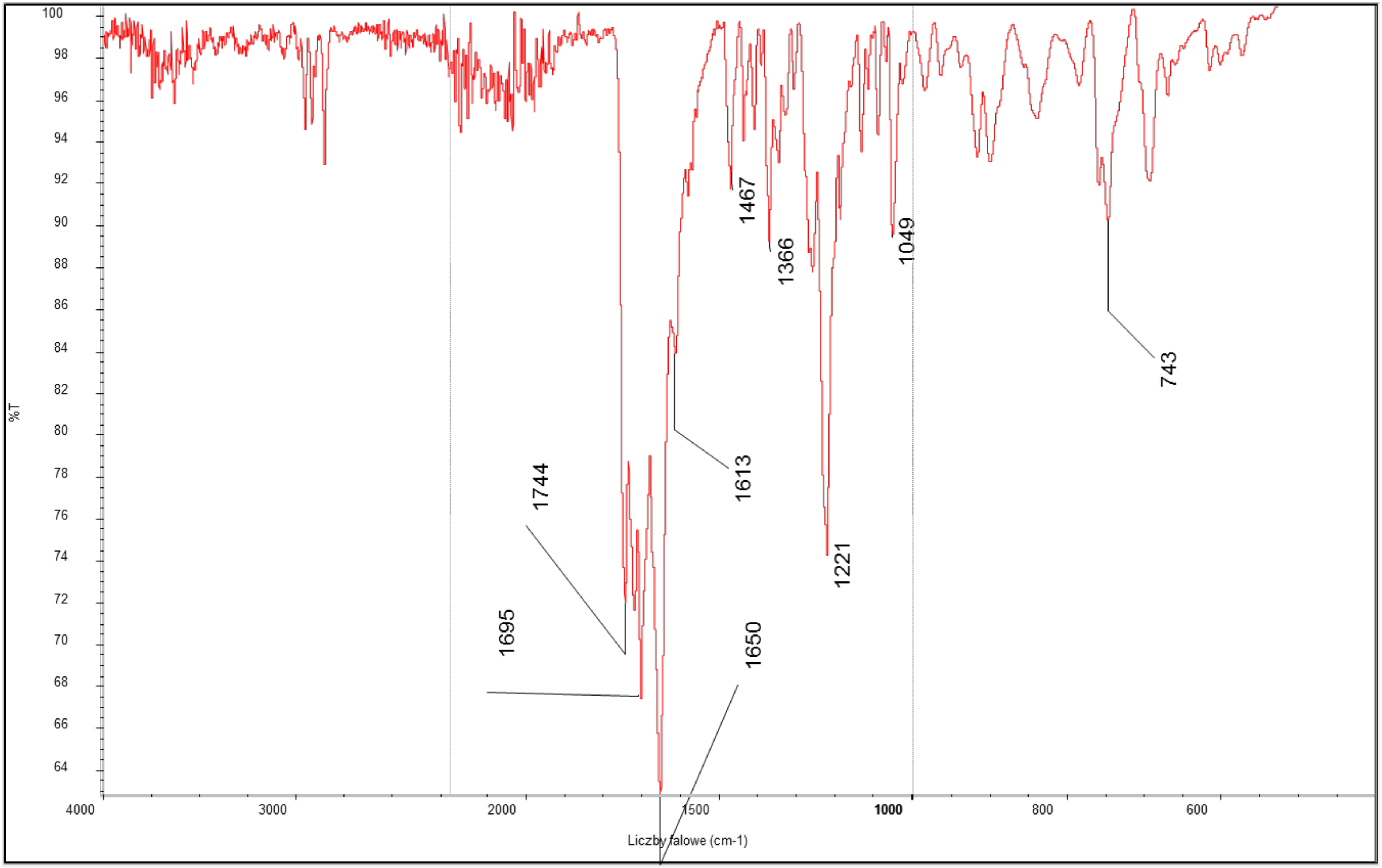
100
98
96
94
92
90
88
86
84
82
80
78
76
74
72
70
68
66
64
%T
4000
3000
2000
1500
1000
800
600
Liczby falowe (cm-1)
1744
1695
1650
1613
1467
1366
1221
1049
743

**Sample Name**	PG 60	**Position**	P1-B1	**Instrument Name**	Instrument 1
**User Name**		**Inj Vol**	0.5	**InjPosition**	
**Sample Type**	Sample	**IRM Calibration Status**	Success	**Data Filename**	6min_MS 2-6minMeOH.m PG 60.d
**ACQ Method**	6min_MS 2-6minMeOH.m	**Comment**		**Acquired Time**	10/2/2019 2:49:16 PM

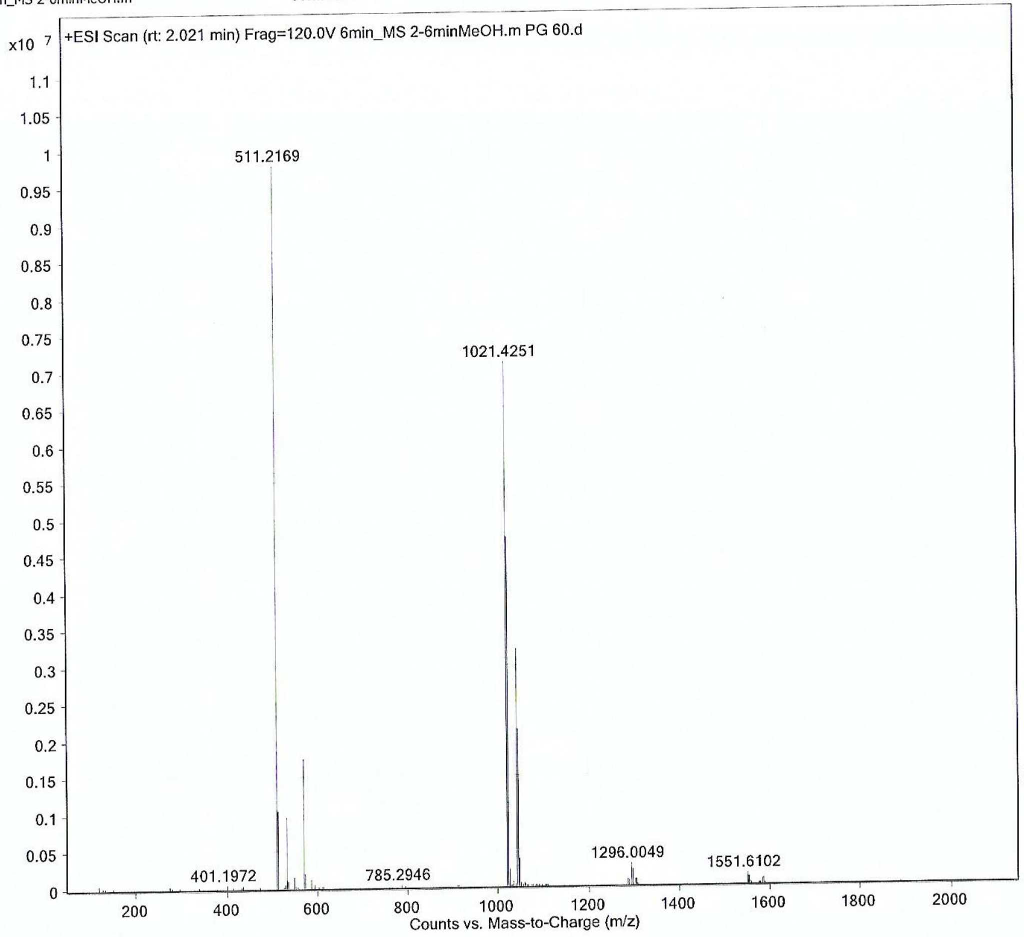

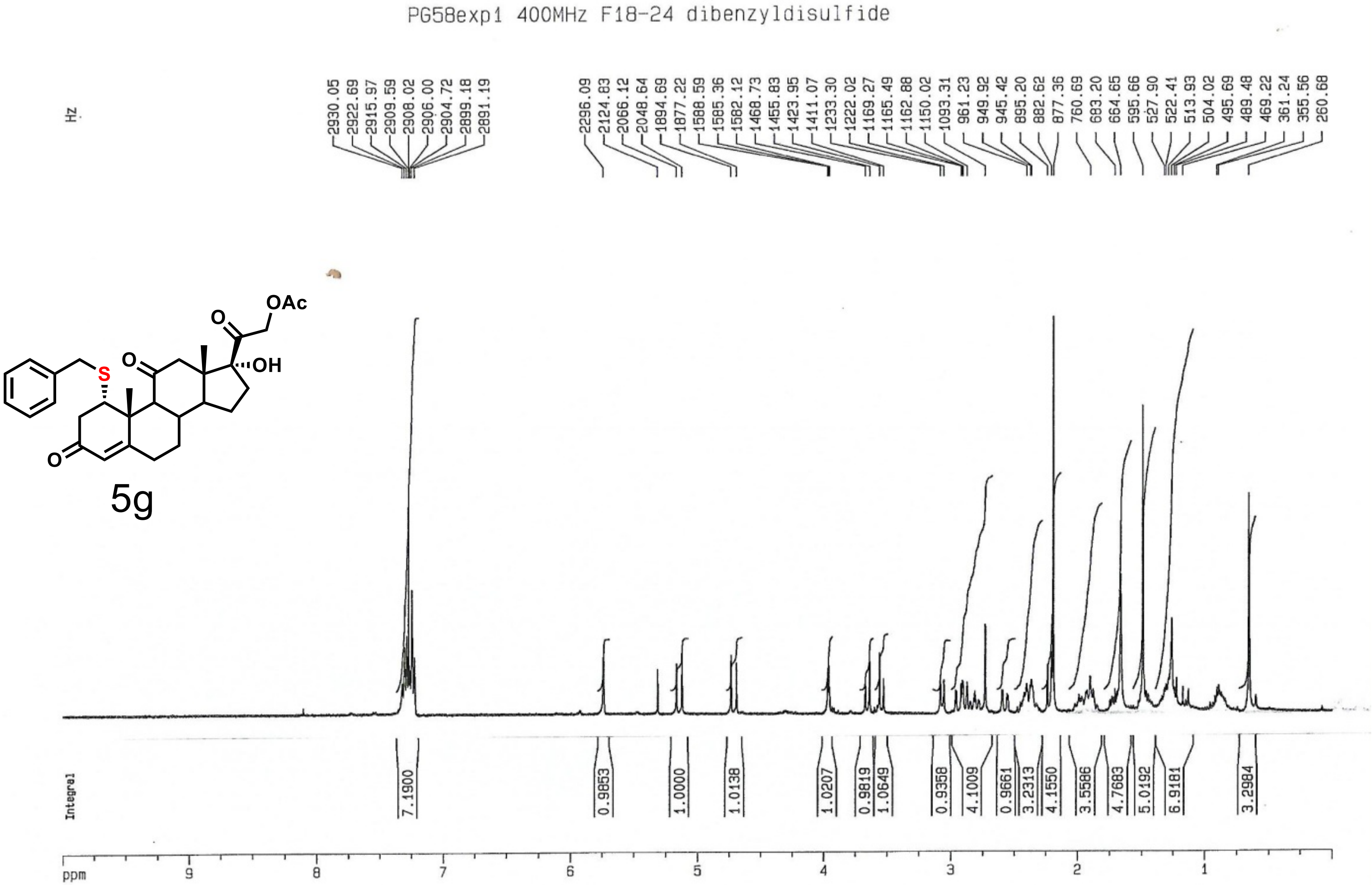

OAc
O
O
S
OH
O
PG58exp1 400MHz F18-24 dibenzyldisulfide
Hz
2930.05
2922.69
2915.97
2909.59
2908.02
2906.00
2904.72
2899.18
2891.19
2296.09
2124.83
2066.12
2048.64
1894.69
1877.22
1588.59
1585.36
1582.12
1468.73
1455.83
1423.95
1411.07
1233.30
1222.02
1169.27
1165.49
1162.88
1150.02
1093.31
961.23
949.92
945.42
895.20
882.62
877.36
760.69
683.20
664.65
595.66
527.90
522.41
513.93
504.02
495.69
489.48
469.22
361.24
355.56
260.68
Integral
7.1900
0.9853
1.0000
1.0138
1.0207
0.9819
1.0649
0.9358
4.1009
0.9661
3.2313
4.1550
3.5586
4.7683
5.0192
6.9181
3.2984
ppm
9
8
7
6
5
4
3
2
1

5g

PG58 F18-24 CDCl3 400MHz 13C JMODE

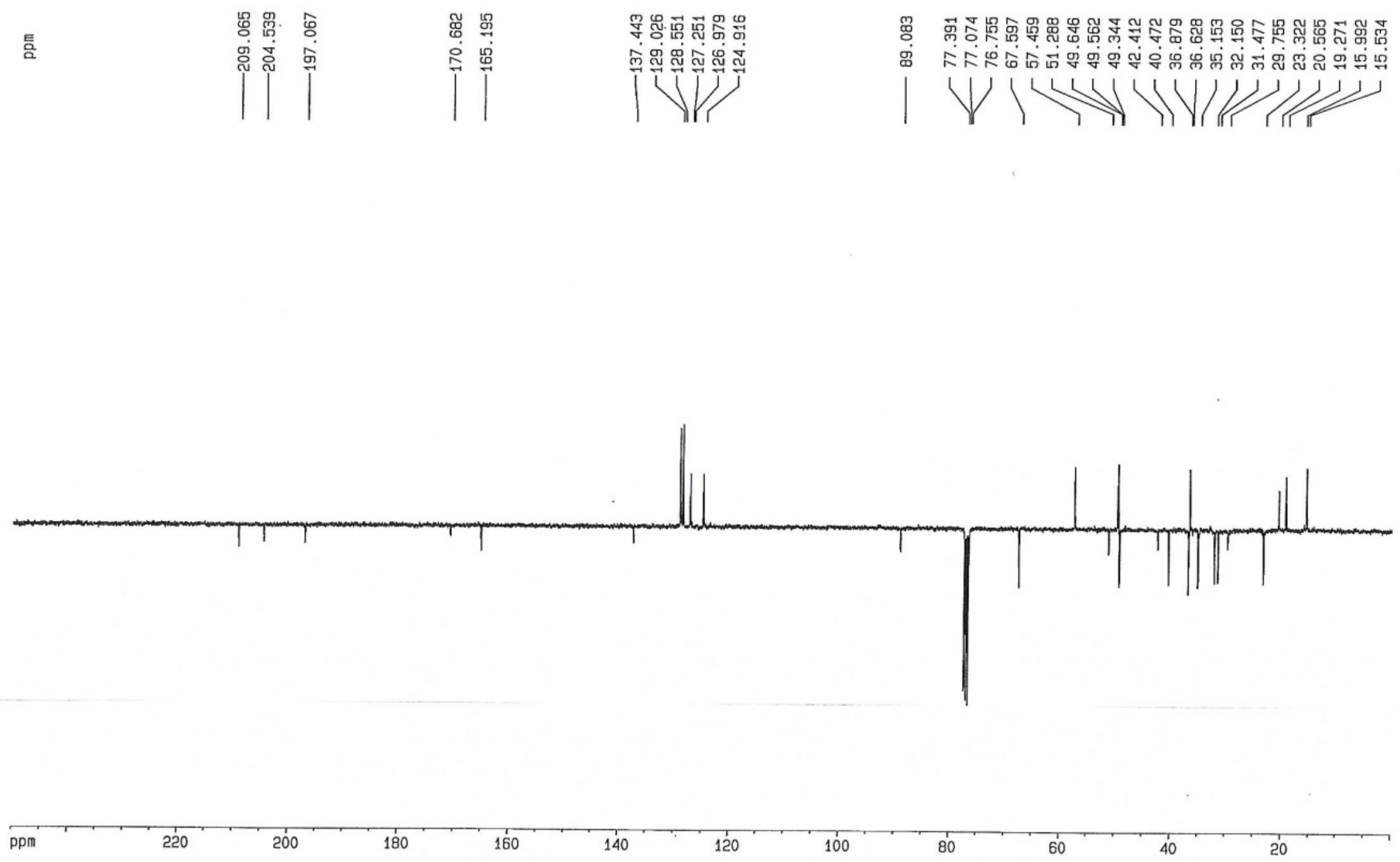

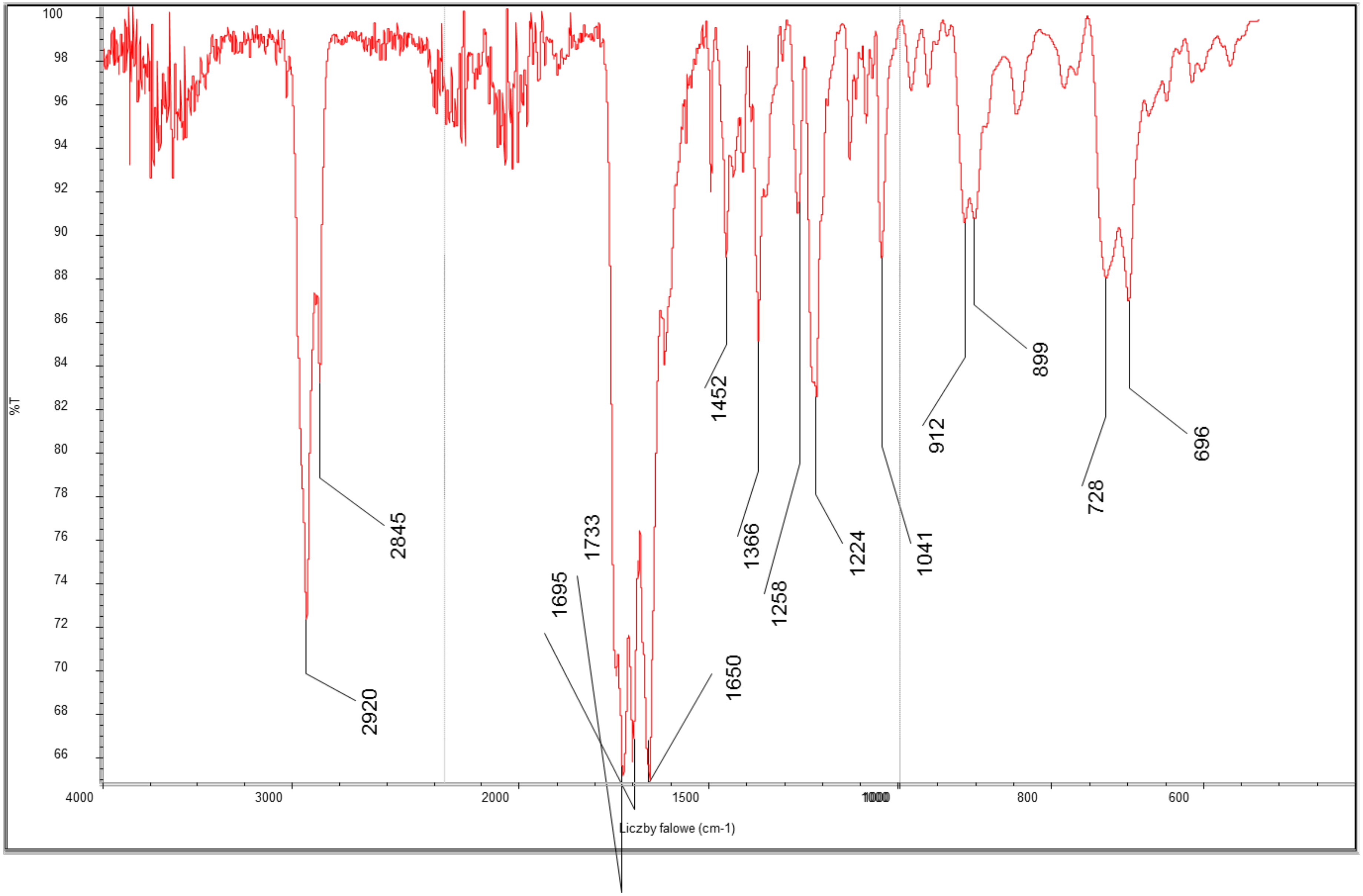

%T
100
98
96
94
92
90
88
86
84
82
80
78
76
74
72
70
68
66
4000
3000
2000
1500
1000
800
600
Liczby falowe (cm-1)
2920
2845
1733
1695
1650
1452
1366
1258
1224
1041
912
899
728
696

**Sample Name**	Unavailable	**Position**	Unavailable	**Instrument Name**	Unavailable
**User Name**	Unavailable	**Inj Vol**	Unavailable	**InjPosition**	Unavailable
**Sample Type**	Unavailable	**IRM Calibration Status**	Success	**Data Filename**	6min_MS 2-6minMeOH.m PG 58.d
**ACQ Method**		**Comment**	Sample information is unavailable	**Acquired Time**	Unavailable

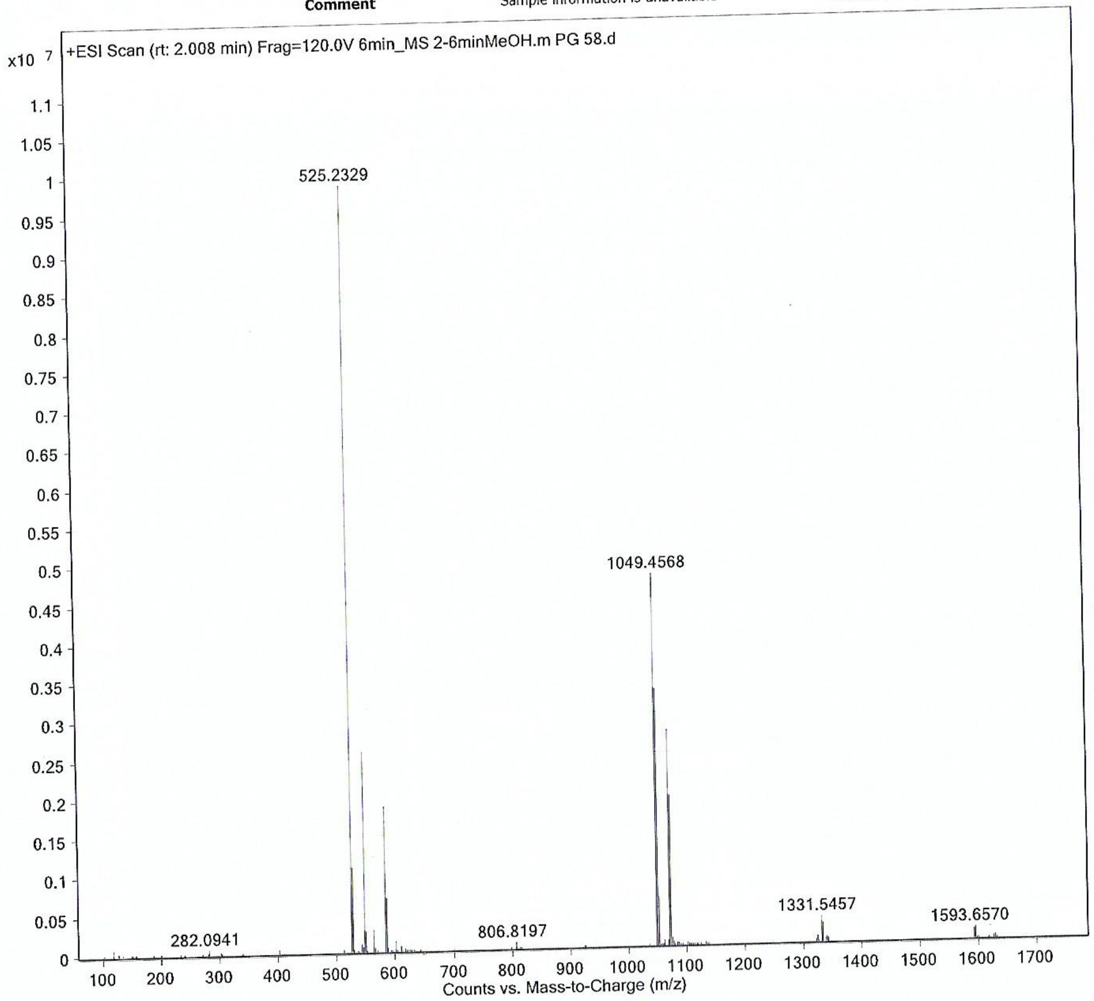

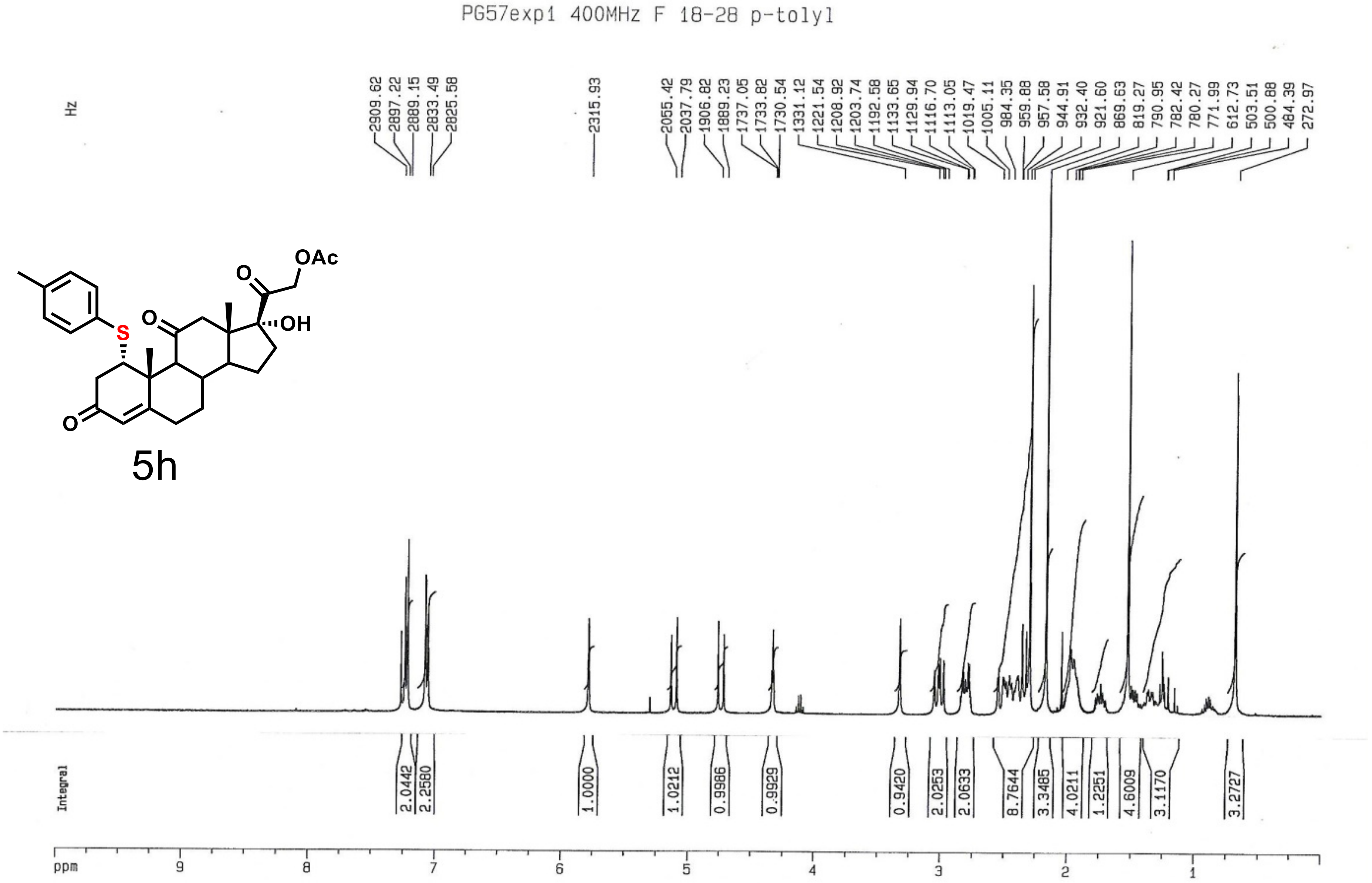
PG57exp1 400MHz F 18-28 p-tolyl
Hz
2909.62
2897.22
2889.15
2833.49
2825.58
2315.93
2055.42
2037.79
1906.82
1889.23
1737.05
1733.82
1730.54
1331.12
1221.54
1208.92
1203.74
1192.58
1133.65
1129.94
1116.70
1113.05
1019.47
1005.11
984.35
959.88
957.58
944.91
932.40
921.60
869.63
819.27
790.95
782.42
780.27
771.99
612.73
503.51
500.88
484.39
272.97
Integral
2.0442
2.2580
1.0000
1.0212
0.9986
0.9929
0.9420
2.0253
2.0633
8.7644
3.3485
4.0211
1.2251
4.6009
3.1170
3.2727
ppm
9
8
7
6
5
4
3
2
1
OAc
O
OH
O
S
O

5h

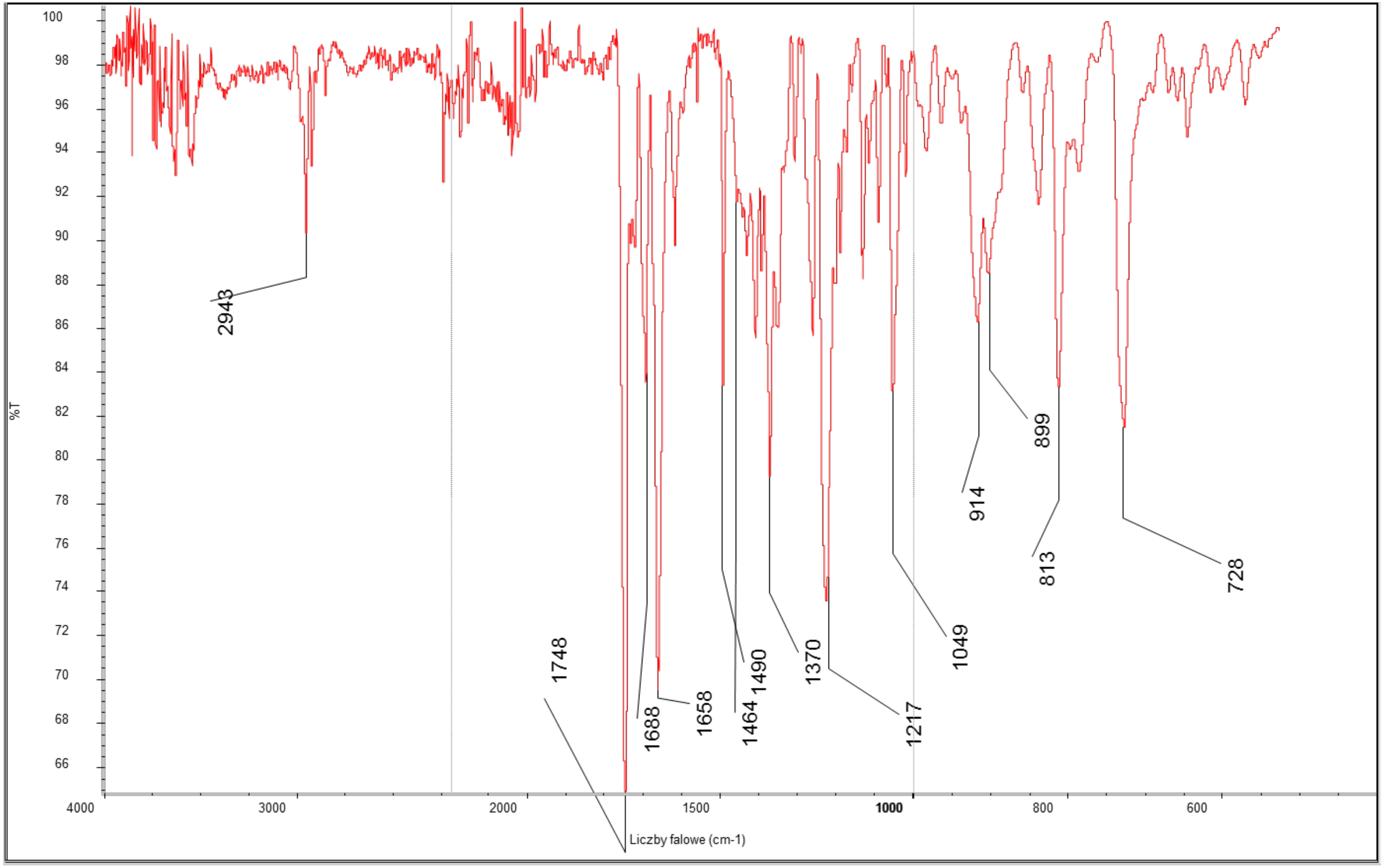

%T
100
98
96
94
92
90
88
86
84
82
80
78
76
74
72
70
68
66
4000
3000
2000
1500
1000
800
600
Liczby falowe (cm-1)
2943
1748
1688
1658
1464
1490
1370
1217
1049
914
899
813
728

**Sample Name**	PG 57	**Position**	P1-B1	**Instrument Name**	Instrument 1
**User Name**		**Inj Vol**	0.1	**InjPosition**	
**Sample Type**	Sample	**IRM Calibration Status**	Success	**Data Filename**	6min_MS 2-6minMeOH.m PG 57.d
**ACQ Method**	6min_MS 2-6minMeOH.m	**Comment**		**Acquired Time**	10/2/2019 3:27:17 PM

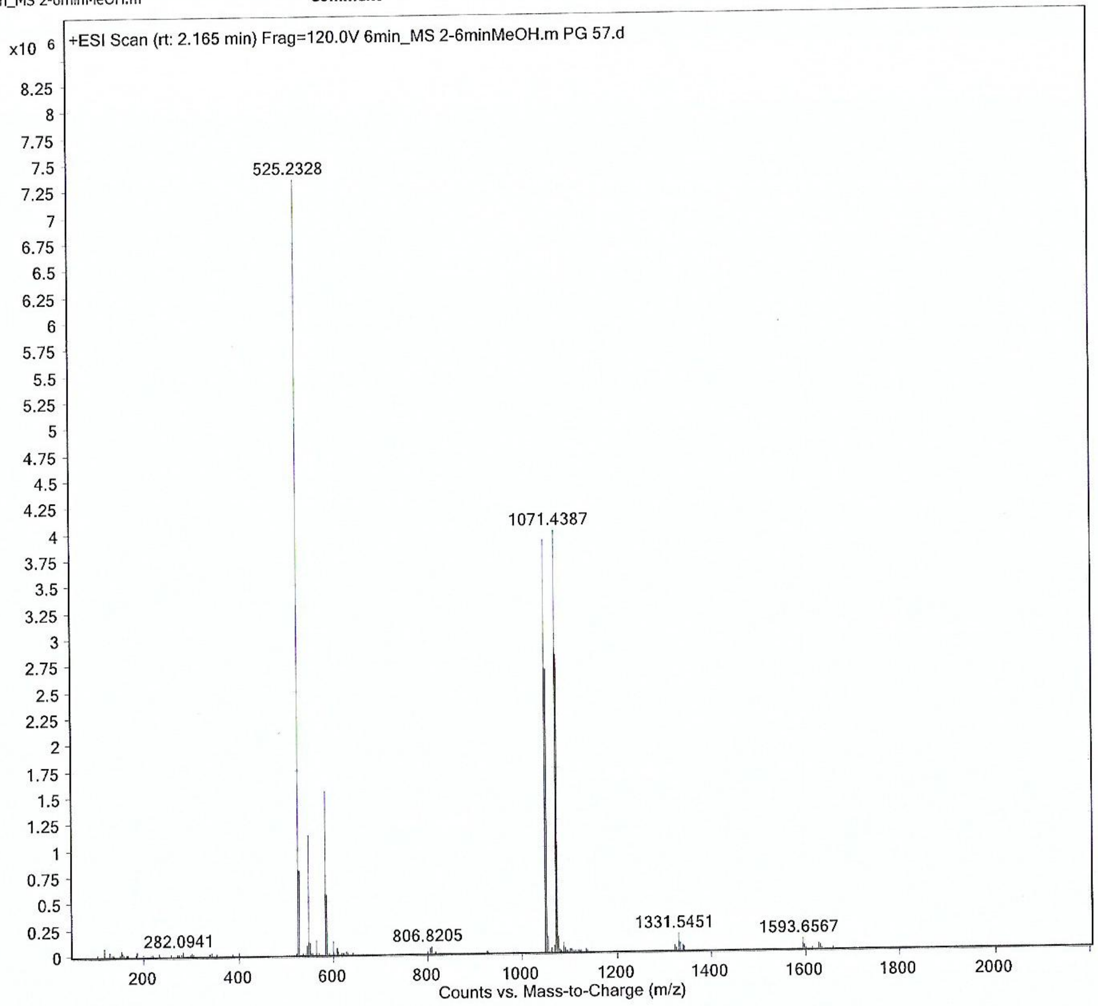

Supplement: Supplementary file 1 [file ijms-23-03022-s001.zip › ijms-1639440-supplementary.pdf]
